# Association between potential factors and dry eye disease: A systematic review and meta-analysis

**DOI:** 10.1097/MD.0000000000041019

**Published:** 2024-12-27

**Authors:** Kuiliang Yang, Shangcao Wu, Lan Ke, Han Zhang, Shanshan Wan, Mingzhi Lu, Jiewen Mao, Yuelan Gao, Yanning Yang, Yiqiao Xing, Wanju Yang

**Affiliations:** a Aier Eye Hospital of Wuhan University, Wuhan, China; b Eye Center, Renmin Hospital of Wuhan University, Wuhan, China.

**Keywords:** dry eye disease, ocular surface diseases, psychological conditions, risk factors

## Abstract

**Background:**

The 2017 TFOS DEWS II report provided an overview of the epidemiology of dry eye disease (DED) and identified several potential risk factors. This study aimed to conduct a meta-analysis on these potential risk factors.

**Methods:**

A comprehensive systematic search was conducted in PubMed, Embase, Web of Science, and Cochrane Library databases to include observational studies. Two researchers independently extracted adjusted odds ratios (AORs) and their 95% confidence intervals (CIs), and a random-effects model was used to combine the data. Results were reported using odds ratios (ORs) and their 95% CIs.

**Results:**

The meta-analysis results showed that the risk factors for DED were smoking (OR 1.18, 95% CI 1.07–1.29), alcohol consumption (OR 1.18, 95% CI 1.03–1.35), rosacea or acne (OR 1.96, 95% CI 1.56–2.45), allergic conjunctivitis (OR 4.59, 95% CI 3.38–6.23), refractive surgery (OR 1.78, 95% CI 1.05–3.00), diabetes (OR 1.14, 95% CI 1.06–1.22), thyroid disease (OR 1.57, 95% CI 1.36–1.82), viral infections (OR 1.54, 95% CI 1.33–1.78), anxiety (OR 2.39, 95% CI 1.30–4.39), depression (OR 1.59, 95% CI 1.39–1.82), post-traumatic stress disorder (OR 1.43, 95% CI 1.42–1.45), and stress (OR 1.59, 95% CI 1.24–2.05). However, there was no significant association between Hispanic ethnicity, menopause, past smoking, current smoking, multivitamin use, and DED.

**Conclusion:**

These findings provide valuable insights for further research on the prevention and treatment of dry eye disease.

## 1. Introduction

Dry eye disease (DED) is a chronic inflammatory condition of the ocular surface characterized by low tear film stability resulting from disruptions in tear quality, quantity, and dynamics.^[1]^ DED is becoming a common ophthalmic disease, especially in Asia, with reported prevalence rates for experiencing of at least one DED symptom ranging from 13.9% to 28.3%.^[2]^ This variance may be consequent to inconsistent diagnostic criteria and evaluation methods, as well as to differences between the structures and cultures of the populations being examined.^[3]^

DED is a multifactorial disease.^[4]^ Higher DED prevalence in recent years may reflect ongoing evolution in populations’ exposures to risk factors.^[5]^ Previous studies have identified several risk factors for DED, including aging, female sex, Asian race, meibomian gland dysfunction, connective tissue diseases, Sjögren Syndrome, androgen deficiency, computer use, contact lens wear, hormone replacement therapy, and hematopoietic stem cell transplantation.^[3]^ However, it has also been suggested that there may be other probable or inconsistent risk factors, such as Hispanic ethnicity,^[6]^ menopause,^[7]^ smoking,^[8]^ alcohol consumption, diabetes, thyroid disease, viral infections,^[9]^ ocular surface diseases or surgeries (rosacea or acne, allergic conjunctivitis, refractive surgery), psychological conditions, and medication use (beta-blockers, diuretics, multivitamins, contraceptives). Assessing the association between potential factors and DED can facilitate the implementation of early intervention measures.

To the best of our knowledge, an up-to-date meta-analysis of DED’s potential risk factors based on the Tear Film and Ocular Surface Society Dry Eye Workshop II (TFOS DEWS II) epidemiology report has not yet been published. In this study, we present the research findings of a systematic review based on potential risk factors published in the TFOS DEW II report. These factors include probable risk factors such as diabetes, thyroid disease, rosacea, refractive surgery, allergic conjunctivitis, psychiatric conditions, diuretics, and beta-blockers. In addition, inconsistent factors such as Hispanic ethnicity, menopause, smoking, alcohol consumption, acne, multivitamins, and oral contraceptives were investigated in published studies. Meta-analysis was conducted to examine the strength of association between these factors and DED.

## 2. Methods

This study adhered to the Declaration of Helsinki. It was registered prospectively on Prospero (ID: CRD42021283655) with its protocol accessible online and was carried out in accordance with PRISMA (Preferred Reporting Items for Systematic Reviews and Meta-Analyses) guidelines,^[10]^ including a systematic literature search, establishment of inclusion and exclusion criteria, evaluation of study quality, and prescribed methods for data extraction and analysis. The meta-analysis based on public literature is not applicable for ethical approval.

### 2.1. Search strategy

A systematic search strategy was developed in consultation with a medical statistician and a librarian. The gold-standard two-step process of title/abstract screening and full-text screening was strictly followed by 2 review authors independently. Four databases, including PubMed, Embase, Web of Science, and Cochrane Library, were searched. The electronic databases were searched from January 1, 2000, to December 31, 2022. The Mesh terms utilized included “dry eye disease,” “dry eye syndrome,” “syndrome, dry eye,” and “risk factor.” (Supplemental Digital Content 1, http://links.lww.com/MD/O218).

### 2.2. Inclusion and exclusion criteria

The following criteria were applied to determine the inclusion of published studies: The research should be primary epidemiological studies focusing on risk factors for DED, including case-control studies, cohort studies, and cross-sectional studies. The association between risk factors and DED should be reported using adjusted odds ratios, controlling for confounding factors, or reported OR through multivariable logistic regression models. The studies should be original research published in English, and complete study characteristics should be provided, including authors, country, year of publication, and participant demographics (mean age, gender, and country). Two authors independently assessed the eligibility of abstracts and titles. In cases where it was unclear whether an article met the inclusion criteria, the full text was carefully reviewed. A small number of similar studies will be combined, such as rosacea or acne, viral infections (including hepatitis B, hepatitis C, HIV). Disagreements were resolved through team discussions.

### 2.3. Risk of bias assessment

Based on the Cochrane Handbook for Systematic Reviews,^[11]^ we conducted a risk of bias assessment using the Newcastle-Ottawa Scale (NOS) for case-control and cohort studies. For cross-sectional studies, a modified version of the NOS (Supplemental Digital Content 2, http://links.lww.com/MD/O218) was employed following discussions. The assessment was independently performed by two researchers to evaluate the risk of bias in the included studies. Articles of inadequate quality, insufficient data, or suspected duplicate publication were excluded during the assessment process.

### 2.4. Statistical analysis

The random-effects model was employed to combine the odds ratios (ORs) and their 95% confidence intervals (CIs). Heterogeneity among included studies was assessed using the I2 statistic. Subgroup analysis based on study type was conducted to identify the sources of heterogeneity. Sensitivity analysis was performed by sequentially excluding studies with large sample sizes and significant result differences to test the stability of the overall effect estimate. If the conclusions remained unchanged, the stability of the included studies was considered good and the results were deemed reliable. Reasons contributing to different conclusions were analyzed. Funnel plots, Egger’s test, and Begg’s test were utilized to investigate publication bias. All analyses were conducted using R software version 4.3.0. The significance level for all pooled results was set at .05, with a two-sided test.

## 3. Results

### 3.1. Study selection

**Figure 1.**
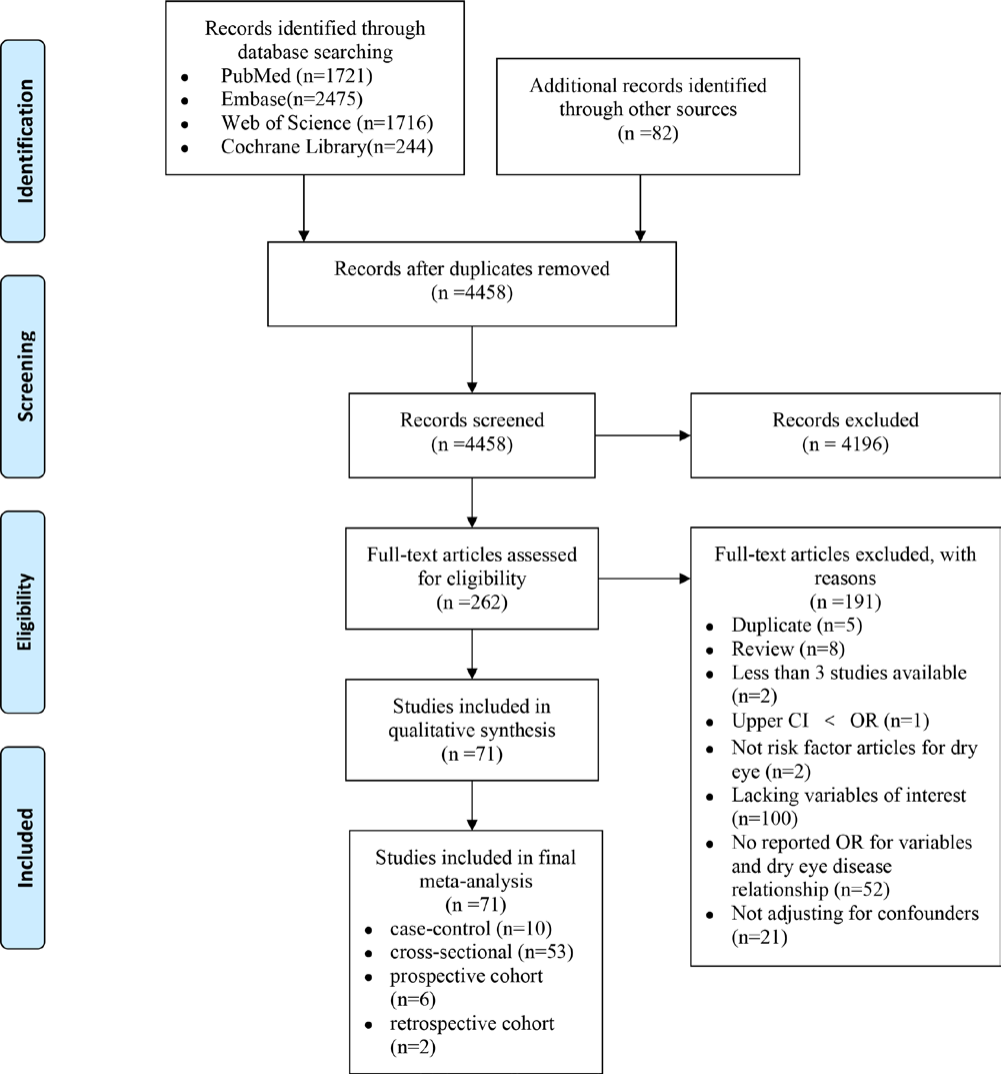
PRISMA flow diagram summarizing the systematic search process for potential risk factors of dry eye disease.

As shown in Figure 1, an extensive literature search yielded 6238 articles. After screening the titles and abstracts of these articles, 262 potentially eligible articles remained. After reviewing the full-text in depth, a total of 71 articles were included (including 8 cohort studies). 191 studies were excluded for the following reasons: duplicate (n = 5), review (n = 8), less than 3 studies available (n = 2), upper CI < OR (n = 1), not risk factor articles for DED (n = 2), lacking variables of interest (n = 100), no reported OR for variables and DED relationship (n = 52), not adjusting for confounders (n = 21).

### 3.2. Study characteristics

The characteristics of the included studies and the participants are summarized in Table 1. A total of 71 studies were included,^[5,12–81]^ comprising 53 cross-sectional studies, 10 case-control studies, 2 retrospective cohort studies, and 6 prospective cohort studies. A total of 11,732,821 individuals were included, and the sample size ranged from 91 to 4,871,504. The mean age of included individuals ranged from 7.5 to 82.2. The definition of DED based on subjective symptoms, including self-reported information and questionnaire responses, was reported in a total of 36 studies. Among these, 1 study employed objective signs, specifically at least one sign identified through ST (tear film stability), TBUT (tear film breakup time), or FSS (frequency and severity of symptoms), to define DED. In addition, a comprehensive evaluation approach incorporating both symptoms and at least one sign, as well as utilizing various criteria such as the 2007 International DED Workshop, TFOS DEWS II diagnostic criteria, and Chinese dry-eye diagnostic criteria, was adopted in 22 studies. Furthermore, nine studies defined DED through clinical diagnosis, including diagnoses made by clinicians and the utilization of the ICD-9 code. Additionally, three studies defined DED based on subjective symptoms (self-reported) as well as clinical diagnosis provided by clinicians. The quality of the studies was assessed using the NOS. Out of the included studies, 19 studies received a rating of 8 stars, 30 studies received 7 stars, 20 studies received 6 stars, and 2 studies received 5 stars. These NOS ratings indicate that the included studies have a good quality.

**Table 1:** Characteristics of studies included in the meta-analysis (include NOS)

References	Study design	Sample size	Mean age or range (years)	Dry eye disease definition	NOS
Moss et al 2000^[12]^	Prospective cohort	3722	65 ± 10	Subjective symptoms (self-reported)	8
Lee et al 2002^[13]^	Cross-sectional	1251	>21	Subjective symptoms (questionnaire)	8
Chia et al 2003^[14]^	Cross-sectional	1075	50–90	Subjective symptoms (questionnaire)	6
Schaumberg et al 2003^[15]^	Cross-sectional	39,876	49–89	Subjective symptoms (questionnaire)	6
Moss et al 2004^[16]^	Prospective cohort	2414	63 ± 10	Subjective symptoms (self-reported)	7
Uchino et al 2008^[17]^	Cross-sectional	4393	22–60	Subjective symptoms (questionnaire)	7
Moss et al 2008^[18]^	Prospective cohort	2827	48–91	Subjective symptoms (self-reported)	8
Schaumberg et al 2009^[19]^	Cross-sectional	25,444	50–99	Subjective symptoms (questionnaire)	7
Viso et al 2009^[20]^	Cross-sectional	619	40–96	Comprehensive assessment (according to the symptoms and at least one sign)	6
Guo et al 2010^[21]^	Cross-sectional	2486	>40	Comprehensive assessment (according to the symptoms and at least one sign)	7
Galor et al 2011^[22]^	Case-control	16,862	21–90	Clinical diagnosis (ICD-9 code)	6
Viso et al 2011^[23]^	Cross-sectional	654	40–96	Comprehensive assessment (according to the symptoms and at least one sign)	7
Galor et al 2012^[24]^	Case-control	2,454,458	21–100	Clinical diagnosis (ICD-9 code)	6
Wang et al 2012^[25]^	Case-control	48,028	52.4 ± 17.5	Clinical diagnosis (ICD-9 code)	7
Abokyiet al. 2012^[26]^	Retrospective cohort	1147	≥12	Comprehensive assessment (according to the symptoms and at least one sign)	7
Malet et al 2013^[27]^	Cross-sectional	963	≥73	Comprehensive assessment (according to the symptoms and at least one sign)	6
Uchino et al 2013^[28]^	Cross-sectional	561	43.3 ± 9.1	Subjective symptoms (questionnaire)	7
Fernandez et al 2013^[29]^	Cross-sectional	248	≥50	Subjective symptoms (questionnaire)	7
Ahn et al 2014^[30]^	Cross-sectional	11,666	49.9 ± 16.7	Subjective symptoms (questionnaire)	8
Vehof et al 2014^[31]^	Cross-sectional	3824	20–87	Clinical diagnosis (a diagnosis of DED made by a clinician)	5
Paulsen et al 2014^[32]^	Prospective cohort	3275	21-84	Subjective symptoms (self-reported)	7
Yang et al 2015^[33]^	Case-control	1908	≥20	Comprehensive assessment (the 2007 International Dry eye disease WorkShop)	7
Chen et al 2015^[34]^	Case-control	10,325	≥12	Clinical diagnosis (ICD-9 code)	7
Hallak et al 2015^[35]^	Case-control	91	>18	Comprehensive assessment (the 2007 International Dry eye disease WorkShop)	6
Van der Vaart et al 2015^[36]^	Case-control	460,611	>18	Clinical diagnosis (ICD-9 code)	6
Yilmaz et al 2015^[37]^	Case-control	363	≥18	Comprehensive assessment (according to the symptoms and at least one sign)	6
Bakkar et al 2016^[38]^	Cross-sectional	1039	≥18	Subjective symptoms (questionnaire)	8
Olaniyan et al 2016^[39]^	Cross-sectional	363	59.1 ± 13.1	Comprehensive assessment (according to the symptoms and at least one sign)	7
Roh et al 2016^[40]^	Cross-sectional	17,364	≥20	Clinical diagnosis (a diagnosis of DED made by a clinician)	7
Chung et al 2016^[41]^	Cross-sectional	4761	≥19	Subjective symptoms (questionnaire)	7
Yoon et al 2016^[42]^	Cross-sectional	17,542	50.88 ± 16.67	Subjective symptoms (self-reported)	6
Alshamrani et al 2017^[43]^	Cross-sectional	1858	>15	Subjective symptoms (questionnaire)	8
Asiedu et al 2017^[44]^	Cross-sectional	700	18–34	Subjective symptoms (questionnaire)	6
Farrand et al 2017^[45]^	Cross-sectional	75,000	≥18	Subjective symptoms (self-reported)and Clinical diagnosis(a diagnosis of DED made by a clinician)	6
Gong et al 2017^[46]^	Cross-sectional	1015	NA	Comprehensive assessment (according to the symptoms and at least one sign)	8
Lee et al 2017^[47]^	Cross-sectional	3,265,894	≥21	Clinical diagnosis (ICD-9 code)	6
Titiyal et al 2018^[48]^	Cross-sectional	15,625	21–40	Subjective symptoms (questionnaire)	7
Ferrero et al 2018^[49]^	Cross-sectional	1045	82.2 ± 3.8	Comprehensive assessment (according to the symptoms and at least one sign)	7
Ben-Eli et al 2019^[50]^	case-control	702	>18	Comprehensive assessment (according to the symptoms and at least one sign)	6
Zhang et al 2019^[51]^	cross-sectional	31,124	NA	Subjective symptoms (questionnaire)	8
Inomata et al 2019^[52]^	cross-sectional	5265	27.2 ± 12.4	Subjective symptoms (questionnaire)	8
Yu et al 2019^[53]^	cross-sectional	23,922	NA	Comprehensive assessment (Chinese dry-eye diagnostic criteria)	7
Kim et al 2019^[54]^	cross-sectional	4185	≥65	Subjective symptoms (self-reported) and Clinical diagnosis (a diagnosis of DED made by a clinician)	6
Hyon et al 2019^[55]^	cross-sectional	566	NA	Subjective symptoms (questionnaire)	7
Inomata et al 2020^[56]^	cross-sectional	4454	27.9 ± 12.6	Subjective symptoms (questionnaire)	8
Shanti et al 2020^[57]^	cross-sectional	769	43.61 ± 18.57	Comprehensive assessment (according to the symptoms and at least one sign)	8
Inomata et al 2020^[58]^	cross-sectional	4454	27.9 ± 12.6	Subjective symptoms (questionnaire)	8
Choi et al 2020^[59]^	cross-sectional	2272	57.5 ± 10.7	Subjective symptoms (questionnaire)	8
Inomata et al 2020^[60]^	cross-sectional	4454	27.9 ± 12.6	Subjective symptoms (questionnaire)	8
Vehof et al 2021^[61]^	cross-sectional	79,866	20–94	Subjective symptoms (questionnaire)	7
Wang et al 2021^[62]^	cross-sectional	372	39 ± 22	Comprehensive assessment (TFOS DEWS-II diagnostic criteria)	7
Hu et al 2021^[63]^	cross-sectional	486	20–59	Comprehensive assessment (according to the symptoms and at least one sign)	7
Chatterjee et al 2021^[64]^	cross-sectional	2378	≥20	Subjective symptoms (questionnaire)	7
Choi et al 2021^[65]^	cross-sectional	475	62.7 ± 8.6	Subjective symptoms (questionnaire)	6
Yang et al 2021^[66]^	cross-sectional	2140	23.4 ± 5.2	Subjective symptoms (questionnaire)	6
Wu et al 2021^[67]^	cross-sectional	1287	61.24 ± 9.54	Comprehensive assessment (according to the symptoms and at least one sign)	7
Magno et al 2021^[68]^	prospective cohort	77,145	50.6 ± 12.2	Subjective symptoms (questionnaire)	8
Wolpert et al 2021^[69]^	prospective cohort	79,606	20–97	Subjective symptoms (questionnaire)	8
Talens-Estarelles et al 2022^[70]^	case-control	851	17–51	Subjective symptoms (questionnaire)	8
Khorshed et al 2022^[71]^	cross-sectional	269	≥18	Comprehensive assessment (according to the symptoms and at least one sign)	7
Zeleke et al 2022^[72]^	cross-sectional	423	NA	Subjective symptoms (questionnaire)	6
García-Marqués et al 2022^[73]^	cross-sectional	120	47.0 ± 22.8	Comprehensive assessment (TFOS DEWS-II diagnostic criteria)	5
An et al 2022^[74]^	cross-sectional	16,471	≥20	Subjective symptoms (self-reported) and Clinical diagnosis (a diagnosis of DED made by a clinician)	8
Dossari et al 2022^[75]^	cross-sectional	1381	≥18	Subjective symptoms (questionnaire)	8
Ma et al 2022^[76]^	cross-sectional	2694	7.49 ± 0.50	Comprehensive assessment (according to the symptoms and at least one sign)	7
Garg et al 2022^[77]^	cross-sectional	820	> 18	Comprehensive assessment (according to the symptoms and at least one sign)	6
Bikbov et al 2022^[78]^	cross-sectional	5153	58.5 ± 10.5	Objective sign (at least one sign)	6
Cartes et al 2022^[79]^	cross-sectional	1450	21.1 ± 2.7	Subjective symptoms (questionnaire and self-reported)	7
He et al 2022^[80]^	retrospective cohort	4,871,504	15–45	Clinical diagnosis (ICD-9 code)	7
Garcia-Queiruga et al 2023^[5]^	cross-sectional	400	24.3 ± 10.6	Comprehensive assessment (TFOS DEWS-II diagnostic criteria)	7
Alkhaldi et al 2023^[81]^	cross-sectional	4066	≥18	Subjective symptoms (questionnaire)	7

ICD-9 = International Classification of Diseases 9th Revision, NA = not available, NOS = Newcastle-Ottawa Scale, TFOS DEWS-II = Tear Film and Ocular Surface Society Dry Eye Workshop II.

### 3.3. Risk factors for DED

**Figure 2.**
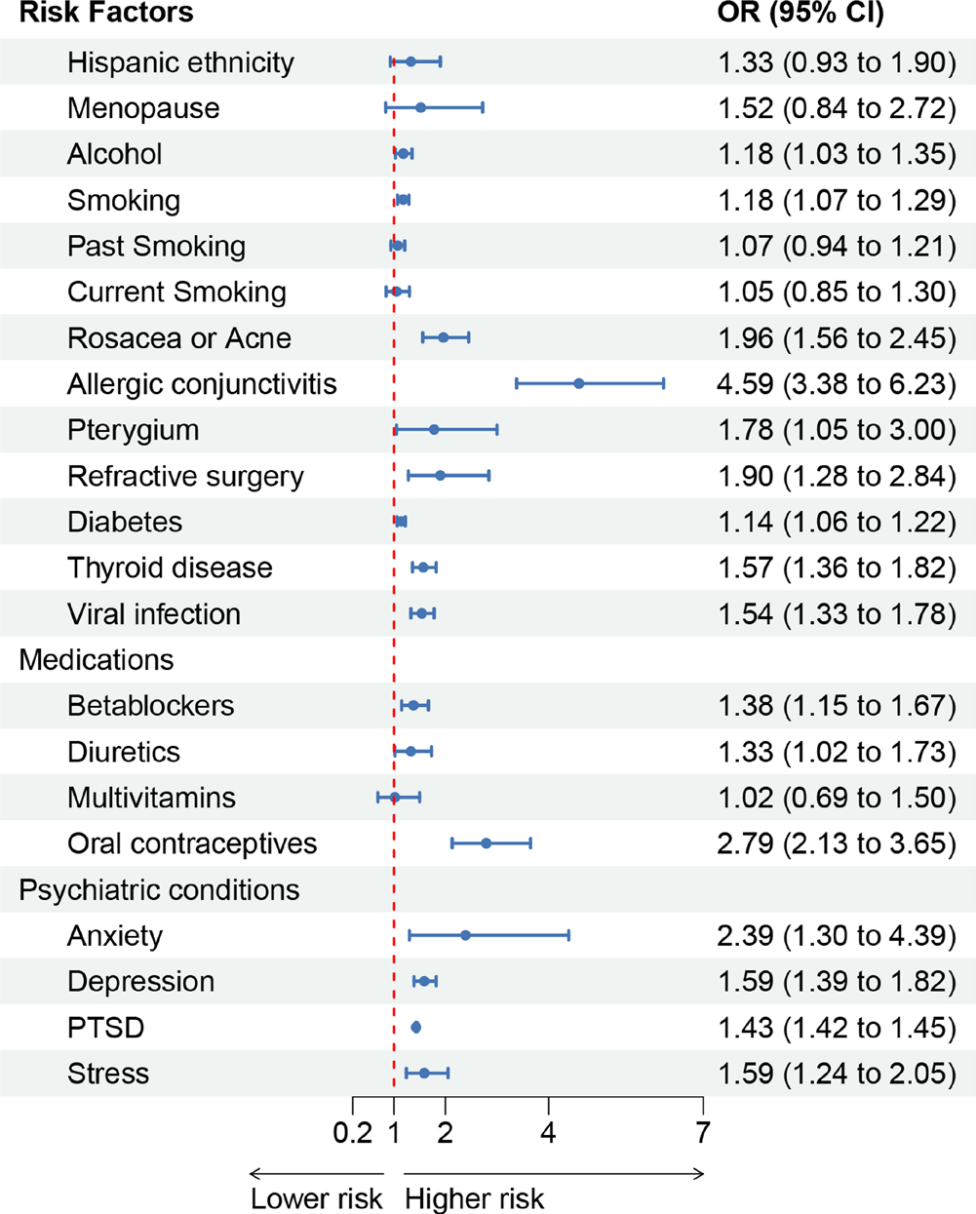
Summary results of potential risk factors for dry eye disease. Reporting the degree of potential risk factors for dry eye disease using the pooled Odds Ratio (OR) and a 95% Confidence Interval (CI).

We observed a significant association between various factors and an increased risk of DED. Alcohol consumption (OR 1.18, 95% CI: 1.03–1.35), smoking (OR 1.18, 95% CI: 1.07–1.29), rosacea or acne (OR 1.96, 95% CI: 1.56–2.45), allergic conjunctivitis (OR 4.59, 95% CI: 3.38–6.23), pterygium (OR 1.78, 95% CI: 1.05–3), refractive surgery (OR 1.9, 95% CI: 1.28–2.84), beta blockers (OR 1.38, 95% CI: 1.15–1.67), diuretics (OR 1.33, 95% CI: 1.02–1.73), oral contraceptives (OR 2.79, 95% CI: 2.13–3.65), diabetes (OR 1.14, 95% CI: 1.06–1.22), thyroid disease (OR 1.57, 95% CI: 1.36–1.82), viral infection (OR 1.54, 95% CI: 1.33–1.78), anxiety (OR 2.39, 95% CI: 1.3–4.39), depression (OR 1.59, 95% CI: 1.39–1.82), PTSD (OR 1.43, 95% CI: 1.42–1.45), and stress (OR 1.59, 95% CI: 1.24–2.05) were all associated with an increased risk of DED (Supplemental Digital Content 3–23, http://links.lww.com/MD/O218 or Figure 2). The number of studies reporting an association with DED risk are as follows: alcohol (n = 17), smoking (n = 20), rosacea or Acne (n = 7), allergic conjunctivitis (n = 3), pterygium (n = 4), refractive surgery (n = 4), beta blockers (n = 5), diuretics (n = 9), oral contraceptives (n = 4), diabetes (n = 24), thyroid disease (n = 21), viral infection (n = 5), anxiety (n = 4), depression (n = 19), PTSD (n = 4), and stress (n = 6). With the exception of potential publication bias in the association between diabetes (*P* value for Egger = .0358 < 0.5), stress (*P* value for Egger = .0408 < 0.5), and DED, no evidence of publication bias was found in other factors (Supplemental Digital Content 24–44, http://links.lww.com/MD/O218). Sensitivity analysis, conducted by sequentially excluding individual studies, demonstrated the robustness of the overall conclusions regarding the association between the remaining risk factors and the risk of DED, except for the study focusing on pterygium (Supplemental Digital Content 45–65, http://links.lww.com/MD/O218).

However, no significant increase in the risk of DED was observed in the studies involving Hispanic ethnicity, menopause, current smoking, past smoking, and use of multivitamins. The stability of these results was confirmed by sensitivity analysis (Supplemental Digital Content 45–65, http://links.lww.com/MD/O218). However, there may be publication bias in the association between current smoking (*P* value for Egger = .0081 < .5; *P* value for Begg = .0381 < .5), menopause (*P* value for Egger = .0481 < .5, *P* value for Begg = .0415 < .5), and DED (Supplemental Digital Content 24–44, http://links.lww.com/MD/O218).

Among the variables of interest included in the analysis, Menopause (*I*^2^ = 44%), past smoking (*I*^2^ = 24%), oral contraceptives (*I*^2^ = 0%), and PTSD (*I*^2^ = 0%) exhibit low levels of heterogeneity. Conversely, Hispanic ethnicity (*I*^2^ = 100%), alcohol (*I*^2^ = 92%), smoking (*I*^2^ = 84%), current smoking (*I*^2^ = 66%), rosacea or acne (*I*^2^ = 85%), allergic conjunctivitis (*I*^2^ = 61%), pterygium (*I*^2^ = 85%), refractive surgery (*I*^2^ = 98%), beta blockers (*I*^2^ = 78%), diuretics (*I*^2^ = 97%), multivitamins (*I*^2^ = 82%), thyroid disease (*I*^2^ = 97%), viral infection (*I*^2^ = 72%), anxiety (*I*^2^ = 97%), depression (*I*^2^ = 98%), and stress (*I*^2^ = 74%) demonstrate higher levels of heterogeneity (Supplemental Digital Content 3–23, http://links.lww.com/MD/O218). Subgroup analysis suggests that the heterogeneity observed in Hispanic ethnicity, smoking, current smoking, allergic conjunctivitis, pterygium, refractive surgery, beta blockers, thyroid disease, anxiety, and stress may originate from cross-sectional studies. The heterogeneity in alcohol, rosacea or acne, viral infection, and depression may stem from case-control studies, while the heterogeneity in diuretics may arise from cohort studies.

## 4. Discussion

We conducted a meta-analysis study on risk factors for DED. This meta-analysis provides an updated systematic review of published studies on DED-associated factors and validates previously inconsistent while potential risk factors. Understanding the relative strength of risk factors for DED may aid in the prevention and treatment of DED.

Previous population-based studies have conducted comprehensive investigations into the risk factors associated with DED and have reported inconsistent associations between DED and comorbidities across multiple organ systems, indicative of a multifactorial etiology.^[82]^ The current study substantiate previous findings emphasizing the significance of psychological disorders, including anxiety and depression, as substantial risk factors for DED. Psychological factors can impact pain perception and contribute to the production of inflammatory cytokines, thereby heightening the susceptibility to DED.^[83]^

DED is closely associated with the ocular surface.^[84]^ Our study provides evidence supporting the association between DED and ocular surface inflammation,^[85]^ specifically allergic conjunctivitis, as well as disruptions in ocular surface integrity, such as pterygium, and corneal nerve changes resulting from refractive surgery.^[86]^ These factors collectively contribute to the development of DED. The findings of our study underscore the crucial role of ocular surface health and protection in the prevention and treatment of DED, highlighting the significance of addressing ocular surface diseases and the careful consideration of surgical interventions.

The relationship between beta-blockers, diuretics, oral contraceptives, and DED may encompass diverse underlying mechanisms.^[87]^ Oral contraceptives could potentially influence hormone levels or modify tear composition,^[88,89]^ thereby impacting ocular surface health. Furthermore, the findings from the meta-analysis reveal no substantial correlation between the usage of multivitamin and DED. These results imply that vitamin intake may not directly influence the risk of developing DED, or there might be other regulatory factors that necessitate consideration.

Unhealthy lifestyle habits, including smoking and alcohol consumption, may contribute to the development of DED, underscoring the importance of interventions targeting these harmful behaviors.^[90,91]^ Our findings revealed a weak correlation between smoking and the risk of DED, with an odds ratio (OR) of 1.18. However, no statistically significant relationship was observed between past or current smoking and DED. The distinction between past smoking and smoking usually implies that smoking has been quit. These findings suggest that quitting smoking could be beneficial in alleviating DED.

Our meta-analysis had several limitations. First, the sample sizes of the included studies are highly variable, which may amplify the impact of specific studies on our results. Second, only English-language publications were included although research on DED risk factors might have been published in other languages. Third, some of the subgroups in our subgroup analyses had small sample sizes. Additionally, the quality of this meta-analysis might have been affected by limitations at the review (e.g., reporting bias) and outcome (e.g., risk of bias) levels.

In conclusion, despite the aforementioned limitations, this study holds significant implications for clinical practice. The screening of diseases associated with the risk of DED, such as rosacea or acne, diabetes, thyroid disease, viral infections, and psychological conditions like anxiety and depression, can provide valuable insights for patient diagnosis. Additionally, it is essential to consider the potential effects of medication use, including beta-blockers, diuretics, and contraceptives, on the development and management of DED.

## Author contributions

**Conceptualization:** Shangcao Wu, Yanning Yang, Yiqiao Xing, Wanju Yang.

**Data curation:** Kuiliang Yang, Shangcao Wu, Lan Ke, Han Zhang, Shanshan Wan, Mingzhi Lu, Jiewen Mao, Yuelan Gao, Yanning Yang, Yiqiao Xing, Wanju Yang.

**Formal analysis:** Kuiliang Yang, Shangcao Wu, Lan Ke, Han Zhang, Shanshan Wan, Mingzhi Lu, Jiewen Mao, Yuelan Gao, Yanning Yang, Yiqiao Xing, Wanju Yang.

**Funding acquisition:** Lan Ke, Wanju Yang.

**Investigation:** Kuiliang Yang, Shangcao Wu, Lan Ke, Han Zhang, Shanshan Wan, Mingzhi Lu, Jiewen Mao, Yuelan Gao, Yanning Yang, Yiqiao Xing, Wanju Yang.

**Methodology:** Kuiliang Yang, Shangcao Wu, Lan Ke, Han Zhang, Shanshan Wan, Mingzhi Lu, Jiewen Mao, Yuelan Gao, Yanning Yang, Yiqiao Xing, Wanju Yang.

**Project administration:** Kuiliang Yang, Shangcao Wu, Lan Ke, Han Zhang, Shanshan Wan, Mingzhi Lu, Jiewen Mao, Yuelan Gao, Yanning Yang, Yiqiao Xing, Wanju Yang.

**Resources:** Kuiliang Yang, Shangcao Wu, Lan Ke, Han Zhang, Shanshan Wan, Mingzhi Lu, Jiewen Mao, Yuelan Gao, Yanning Yang, Yiqiao Xing, Wanju Yang.

**Software:** Kuiliang Yang, Shangcao Wu, Lan Ke, Han Zhang, Shanshan Wan, Mingzhi Lu, Jiewen Mao, Yuelan Gao, Yanning Yang, Yiqiao Xing, Wanju Yang.

**Supervision:** Kuiliang Yang, Shangcao Wu, Lan Ke, Han Zhang, Shanshan Wan, Mingzhi Lu, Jiewen Mao, Yuelan Gao, Yanning Yang, Yiqiao Xing, Wanju Yang.

**Validation:** Kuiliang Yang, Shangcao Wu, Lan Ke, Han Zhang, Shanshan Wan, Mingzhi Lu, Jiewen Mao, Yuelan Gao, Yanning Yang, Yiqiao Xing, Wanju Yang.

**Visualization:** Kuiliang Yang, Shangcao Wu, Lan Ke, Han Zhang, Shanshan Wan, Mingzhi Lu, Jiewen Mao, Yuelan Gao, Yanning Yang, Yiqiao Xing, Wanju Yang.

**Writing – original draft:** Kuiliang Yang.

**Writing – review & editing:** Kuiliang Yang, Shangcao Wu, Lan Ke, Han Zhang, Shanshan Wan, Mingzhi Lu, Jiewen Mao, Yuelan Gao, Yanning Yang, Yiqiao Xing, Wanju Yang.

## Supplementary Material


