# Supplementary material for: Association between potential factors and dry eye disease: A systematic review and meta-analysis
Source: Medicine (Baltimore). 2024 Dec 27;103(52):e41019. doi: 10.1097/MD.0000000000041019 (PMC11688006; doi:10.1097/MD.0000000000041019)

Search strategy

PubMed	#1	"Dry Eye Syndromes"[Mesh]
	#2	(((((((((Dry Eye Syndrome[Title/Abstract]) OR (Dry Eye Disease[Title/Abstract])) OR (Dry Eye Diseases[Title/Abstract])) OR (Dry Eye[Title/Abstract])) OR (Dry Eyes[Title/Abstract])) OR (Evaporative Dry Eye Disease[Title/Abstract])) OR (Evaporative Dry Eye Syndrome[Title/Abstract])) OR (Evaporative Dry Eye[Title/Abstract])) OR (Dry Eye, Evaporative[Title/Abstract])) OR (Evaporative Dry Eyes[Title/Abstract]))
	#3	#1 OR #2
	#4	"Risk Factors"[Mesh]
	#5	((((((((((((((((Factor, Risk) OR (Risk Factor)) OR (Social Risk Factors)) OR (Factor, Social Risk)) OR (Factors, Social Risk)) OR (Risk Factor, Social)) OR (Risk Factors, Social)) OR (Social Risk Factor)) OR (Health Correlates)) OR (Correlates, Health)) OR (Population at Risk)) OR (Populations at Risk)) OR (Risk Scores)) OR (Risk Score)) OR (Score, Risk)) OR (Risk Factor Scores)) OR (Risk Factor Score)) OR (Score, Risk Factor)
	#6	#4 OR #5
	#7	#3 AND #6
Embase	#1	'dry eye syndromes'/exp OR 'dry eye syndromes'
	#2	'dry eye syndrome':ab,ti OR 'dry eye disease':ab,ti OR 'dry eye diseases':ab,ti OR 'dry eye':ab,ti OR 'dry eyes':ab,ti OR 'evaporative dry eye disease':ab,ti OR 'evaporative dry eye syndrome':ab,ti OR 'evaporative dry eye':ab,ti OR 'dry eye, evaporative':ab,ti OR 'evaporative dry eyes':ab,ti
	#3	#1 OR #2
	#4	'risk factors'/exp OR 'risk factors'
	#5	'factor, risk':ab,ti OR 'risk factor':ab,ti OR 'social risk factors':ab,ti OR 'factor, social risk':ab,ti OR 'factors, social risk':ab,ti OR 'risk factor, social':ab,ti OR 'risk factors, social':ab,ti OR 'social risk factor':ab,ti OR 'health correlates':ab,ti OR 'correlates, health':ab,ti OR 'population at risk':ab,ti OR 'populations at risk':ab,ti OR 'risk scores':ab,ti OR 'risk score':ab,ti OR 'score, risk':ab,ti OR 'risk factor scores':ab,ti OR 'risk factor score':ab,ti OR 'score,

		risk factor':ab,ti
	#6	#4 OR #5
	#7	#3 AND #6
Web of science	#1	TS=(Dry Eye Syndromes OR Dry Eye Syndrome OR Dry Eye Disease OR Dry Eye Diseases OR Dry Eye OR Dry Eyes OR Evaporative Dry Eye Disease OR Evaporative Dry Eye Syndrome OR Evaporative Dry Eye OR Dry Eye, Evaporative OR Evaporative Dry Eyes)
	#2	TS=(Risk Factors OR Factor, Risk OR Risk Factor OR Social Risk Factors OR Factor, Social Risk OR Factors, Social Risk OR Risk Factor, Social OR Risk Factors, Social OR Social Risk Factor OR Health Correlates OR Correlates, Health OR Population at Risk OR Populations at Risk OR Risk Scores OR Risk Score OR Score, Risk OR Risk Factor Scores OR Risk Factor Score OR Score, Risk Factor)
Cochrane library	#1	Dry Eye Syndromes
	#2	(Dry Eye Syndromes):ab,ti,kw OR (Dry Eye Syndrome):ab,ti,kw OR (Dry Eye Disease):ab,ti,kw OR (Dry Eye Diseases):ab,ti,kw OR (Dry Eye):ab,ti,kw OR (Dry Eyes):ab,ti,kw OR (Evaporative Dry Eye Disease):ab,ti,kw OR (Evaporative Dry Eye Syndrome):ab,ti,kw OR (Evaporative Dry Eye):ab,ti,kw OR (Dry Eye, Evaporative):ab,ti,kw OR (Evaporative Dry Eyes):ab,ti,kw
	#3	#1 OR #2
	#4	Risk Factors
	#5	(Factor, Risk):ab,ti,kw OR (Risk Factor):ab,ti,kw OR (Social Risk Factors):ab,ti,kw OR (Factor, Social Risk):ab,ti,kw OR (Factors, Social Risk):ab,ti,kw OR (Risk Factor, Social):ab,ti,kw OR (Risk Factors, Social):ab,ti,kw OR (Social Risk Factor):ab,ti,kw OR (Health Correlates):ab,ti,kw OR (Correlates, Health):ab,ti,kw OR (Population at Risk):ab,ti,kw OR (Populations at Risk):ab,ti,kw OR (Risk Scores):ab,ti,kw OR (Risk Score):ab,ti,kw OR (Score, Risk):ab,ti,kw OR (Risk Factor Scores):ab,ti,kw OR (Risk Factor Score):ab,ti,kw OR (Score, Risk Factor):ab,ti,kw
	#6	#4 OR #5
	#7	#3 AND #6

The search covered studies published between January 2000 and December 2022.

SUPPLEMENTAL DIGITAL CONTENT 1

SUPPLEMENTAL DIGITAL CONTENT 2
Newcastle-Ottawa Scale adapted for cross-sectional studies

Selection: (Maximum 5 stars)
1) Representativeness of the sample:
a) Truly representative of the average in the target population. * (all subjects or random sampling)
b) Somewhat representative of the average in the target population. * (non-random sampling)
c) Selected group of users.
d) No description of the sampling strategy.

2) Sample size:
a) Justified and satisfactory. *
b) Not justified.

3) Non-respondents:
a) Comparability between respondents and non-respondents characteristics is established, and the response rate is satisfactory. *
b) The response rate is unsatisfactory, or the comparability between respondents and non-respondents is unsatisfactory.
c) No description of the response rate or the characteristics of the responders and the non-responders.

4) Ascertainment of the exposure (risk factor):
a) Validated measurement tool. **
b) Non-validated measurement tool, but the tool is available or described.*
c) No description of the measurement tool.

Comparability: (Maximum 2 stars)
1) The subjects in different outcome groups are comparable, based on the study design or analysis. Confounding factors are controlled.

a) The study controls for the most important factor (select one). *
b) The study control for any additional factor. *

Outcome: (Maximum 3 stars)
1) Assessment of the outcome:
a) Independent blind assessment. **
b) Record linkage. **
c) Self report. *
d) No description.

2) Statistical test:
a) The statistical test used to analyze the data is clearly described and appropriate, and the measurement of the association is presented, including confidence intervals and the probability level (p value). *
b) The statistical test is not appropriate, not described or incomplete.

SUPPLEMENTAL DIGITAL CONTENT 3

Forest Plot of Hispanic Ethnicity

Study or Subgroup	OR	95% CI	Weight
case-control			
Galor et al (2012)	1.66	[1.60, 1.72]	19.2%
Test for overall effect: $Z = 27.47$ ($P < .01$)			
cross-sectional			
Schaumberg et al (2003)	1.81	[1.18, 2.79]	15.1%
Schaumberg et al (2009)	1.25	[0.93, 1.68]	17.1%
Fernandez et al(2013)	1.25	[0.59, 2.63]	10.5%
Farrand et al (2017)	1.34	[1.19, 1.51]	18.9%
Lee et al(2017)	0.90	[0.89, 0.91]	19.3%
Total (95% CI)	1.24	[0.93, 1.65]	80.8%
Heterogeneity: $Tau^2 = 0.079$; $Chi^2 = 57.94$, $df = 4$ ($P < .01$); $I^2 = 93\%$			
Test for overall effect: $Z = 1.47$ ($P = .14$)			
**Total (95% CI)**	**1.33**	**[0.93, 1.90]**	**100.0%**
Heterogeneity: $Tau^2 = 0.174$; $Chi^2 = 1050.43$, $df = 5$ ($P < .01$); $I^2 = 100\%$			
Test for overall effect: $Z = 1.55$ ($P = .12$)			
Test for subgroup differences: $Chi^2 = 3.94$, $df = 1$ ($P = .05$)			

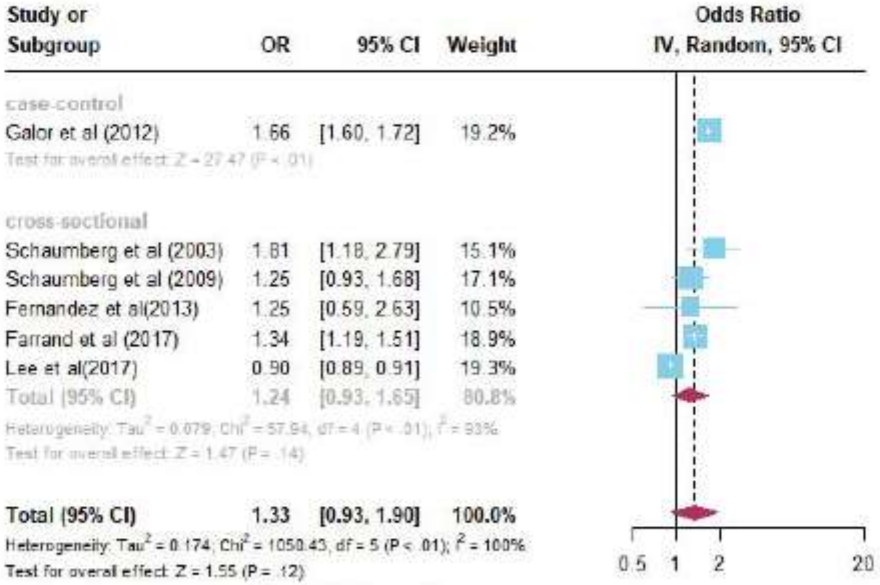

SUPPLEMENTAL DIGITAL CONTENT 4

Forest Plot of Menopause.

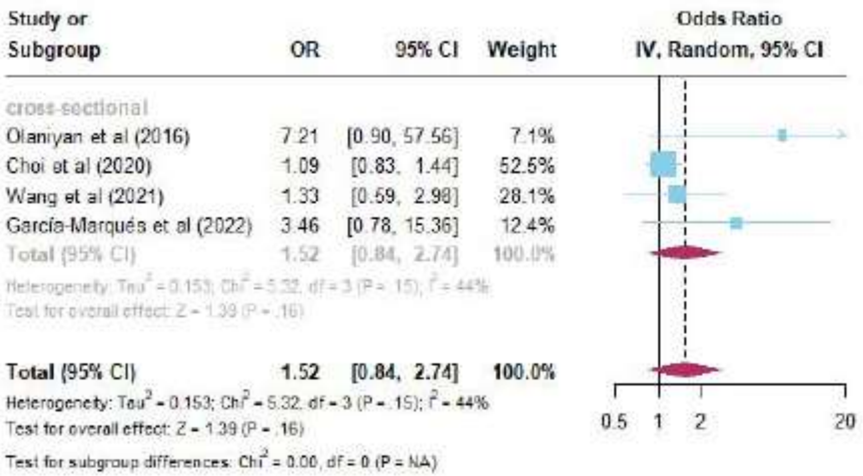

Forest Plot of Alcohol

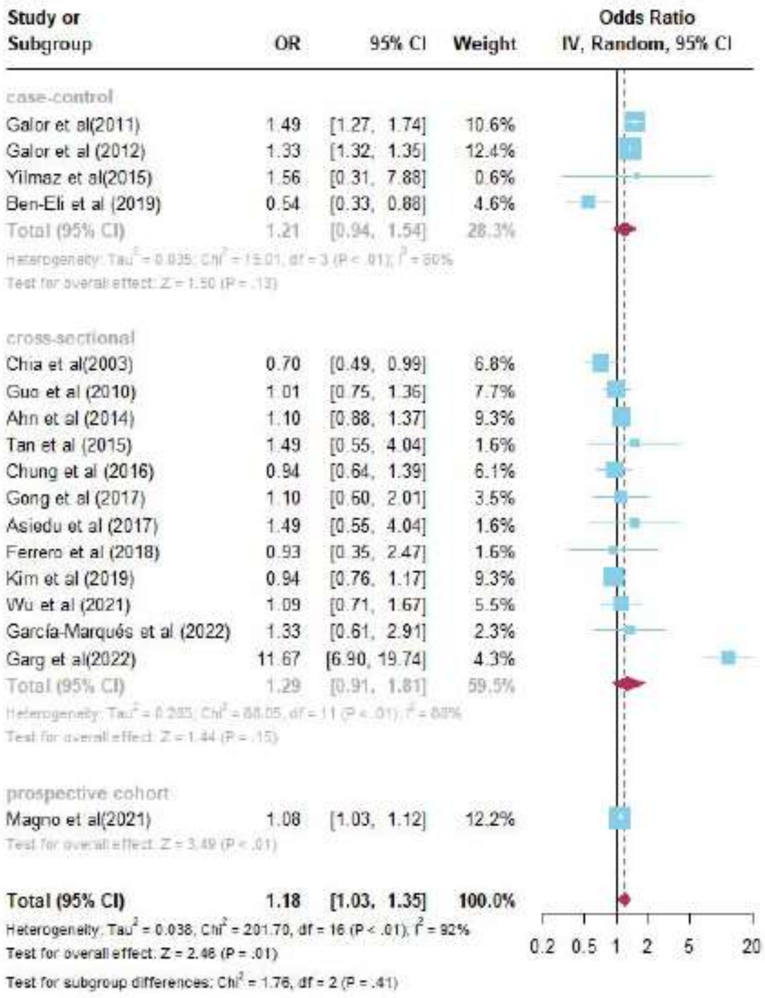

SUPPLEMENTAL DIGITAL CONTENT 5

Forest Plot of Smoking

Study or Subgroup	OR	95% CI	Weight
case-control			
Ben-Eli et al (2019)	0.90	[0.55, 1.47]	2.5%
Test for overall effect: Z = -0.42 (P = .87)			
cross-sectional			
Uchino et al (2008)	0.77	[0.53, 1.12]	3.7%
Guo et al (2010)	1.06	[0.81, 1.39]	5.3%
Ahn et al (2014)	0.90	[0.72, 1.13]	6.2%
Bakkar et al (2016)	1.20	[0.90, 1.60]	5.0%
Gong et al (2017)	0.80	[0.43, 1.50]	1.7%
Lee et al(2017)	1.08	[1.08, 1.08]	10.2%
Titiyal et al (2018)	2.14	[1.65, 2.78]	5.5%
Ferrero et al (2018)	0.73	[0.46, 1.16]	2.8%
Inomata et al (2019)	1.53	[1.31, 1.79]	7.8%
Inomata et al (2020)	2.07	[1.49, 2.88]	4.3%
Shanti et al (2020)	0.96	[0.65, 1.43]	3.5%
Tandon et al (2020)	1.20	[1.05, 1.37]	8.4%
Vehof et al (2021)	0.87	[0.80, 0.94]	9.4%
Chatterjee et al (2021)	1.09	[1.02, 1.16]	9.7%
Yang et al (2021)	1.26	[0.82, 1.93]	3.1%
Khorshed et al (2022)	2.98	[1.58, 5.63]	1.7%
García-Marqués et al (2022)	2.20	[0.93, 5.23]	1.0%
Dossari et al(2022)	1.39	[0.97, 1.99]	3.9%
Cartes et al(2022)	1.32	[0.94, 1.85]	4.2%
Total (95% CI)	1.18	[1.08, 1.30]	97.5%
Heterogeneity: $Tau^2 = 0.021$; $Chi^2 = 116.83$, df = 18 ($P < .01$); $I^2 = 85\%$			
Test for overall effect: Z = 3.61 ($P < .01$)			
**Total (95% CI)**	**1.18**	**[1.07, 1.29]**	**100.0%**

Heterogeneity: $Tau^2 = 0.021$; $Chi^2 = 117.36$, df = 19 ($P < .01$); $I^2 = 84\%$

Test for overall effect: Z = 3.52 ($P < .01$)

Test for subgroup differences: $Chi^2 = 1.16$, df = 1 ($P = .28$)

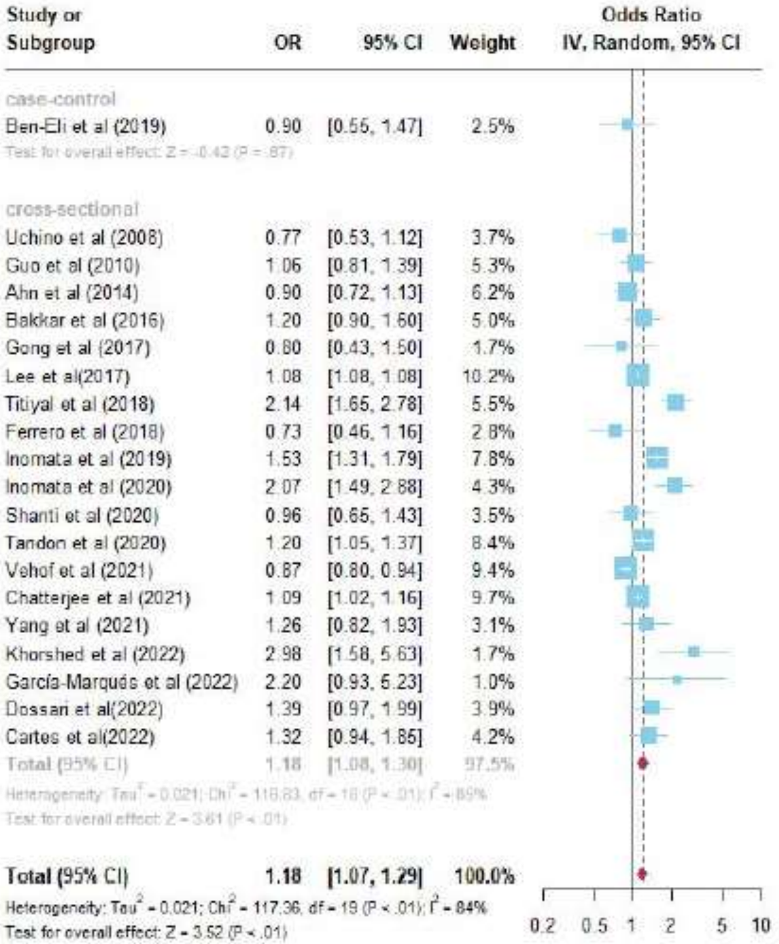

SUPPLEMENTAL DIGITAL CONTENT 6

SUPPLEMTENTAL DIGITAL CONTENT 7

Forest Plot of Past Smoking

Study or Subgroup	OR	95% CI	Weight
**cross-sectional**			
Lee et al (2002)	1.20	[0.60, 2.40]	3.0%
Malet et al (2013)	0.82	[0.54, 1.24]	7.7%
Fernandez et al(2013)	1.74	[0.67, 4.54]	1.6%
Kim et al (2019)	0.80	[0.57, 1.14]	10.3%
Vehof et al (2021)	1.09	[1.03, 1.15]	56.9%
Total (95% CI)	1.01	[0.85, 1.21]	79.5%
Heterogeneity: $Tau^2 = 0.012$; $Chi^2 = 5.54$, df = 4 (P = .24); $I^2 = 28\%$			
Test for overall effect: Z = 0.16 (P = .88)			
**prospective cohort**			
Moss et al (2000)	1.22	[0.97, 1.53]	20.5%
Test for overall effect: Z = 1.74 (P = .08)			
**Total (95% CI)**	**1.07**	**[0.94, 1.21]**	**100.0%**
Heterogeneity: $Tau^2 = 0.006$; $Chi^2 = 6.62$, df = 5 (P = .25); $I^2 = 24\%$			
Test for overall effect: Z = 1.06 (P = .29)			
Test for subgroup differences: $Chi^2 = 1.64$, df = 1 (P = .20)			

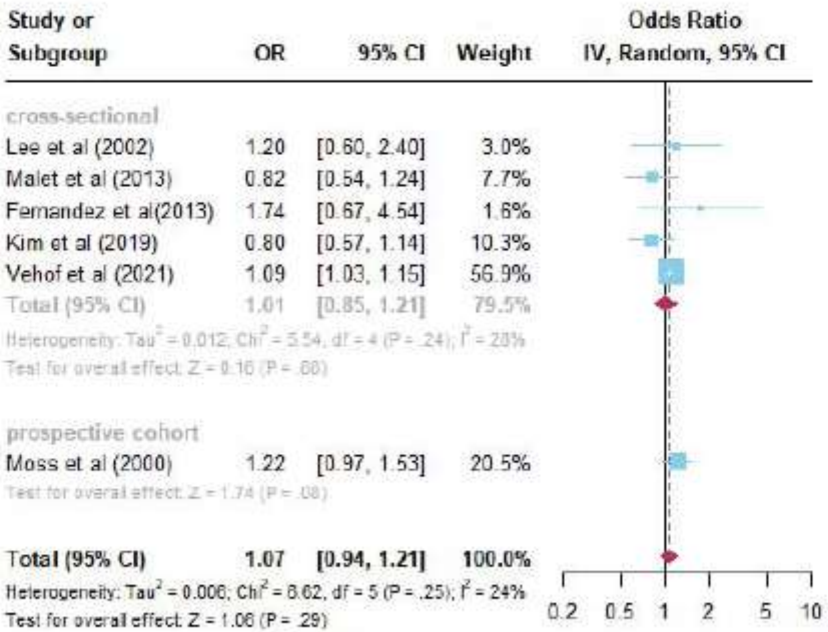

Forest Plot of Current Smoking

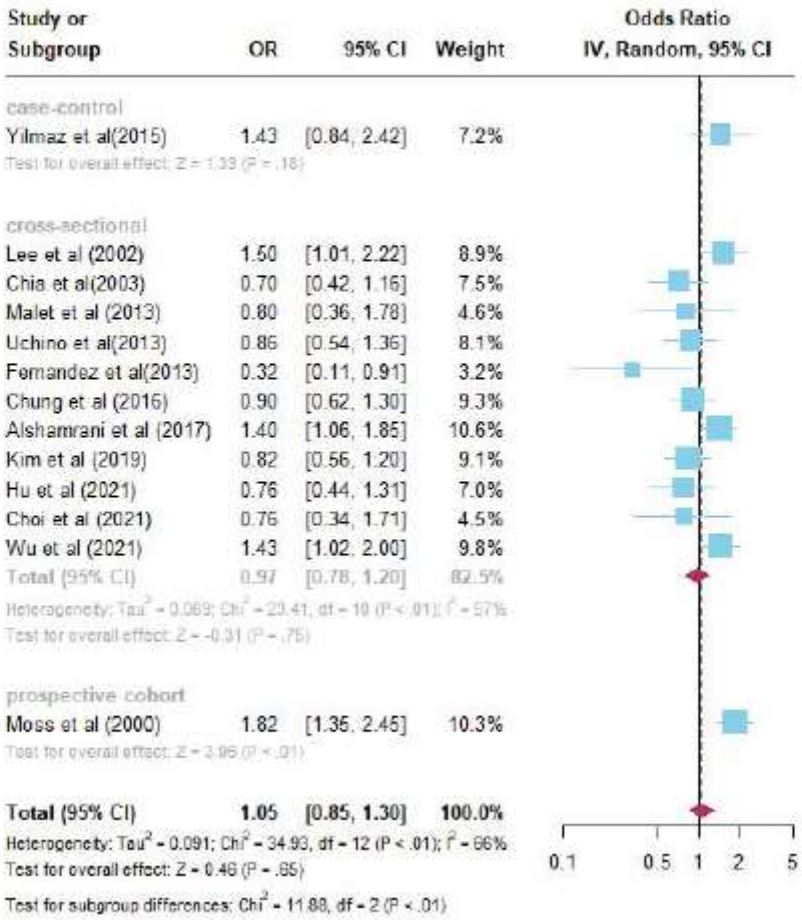

SUPPLEMENTAL DIGITAL CONTENT 8

SUPPLEMENTAL DIGITAL CONTENT 9

Forest Plot of Rosacea or Acne

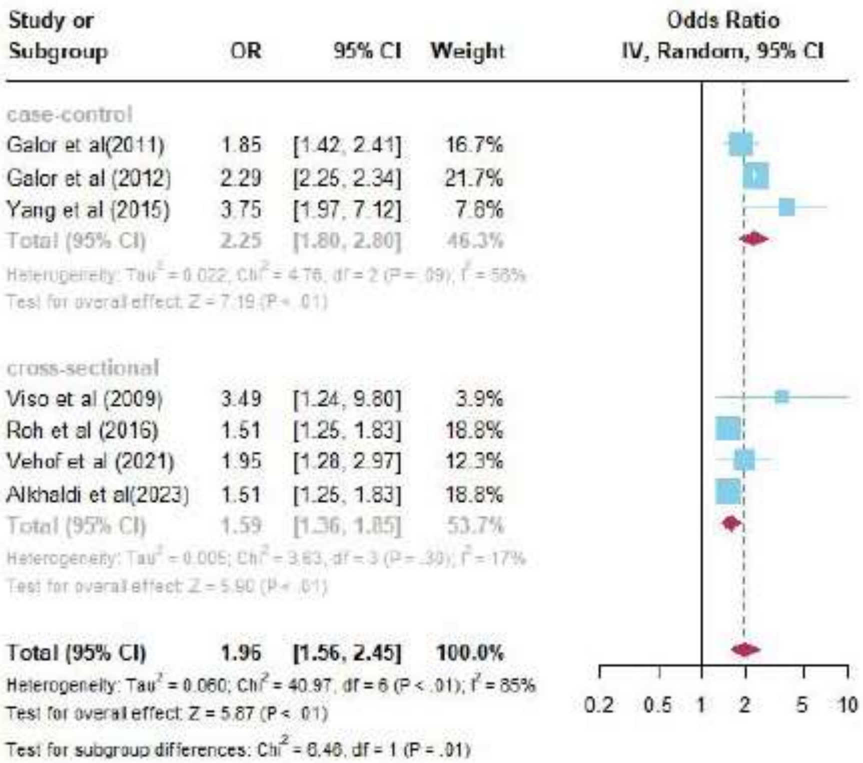

SUPPLEMENTAL DIGITAL CONTENT 10

Forest Plot of Allergic Conjunctivitis

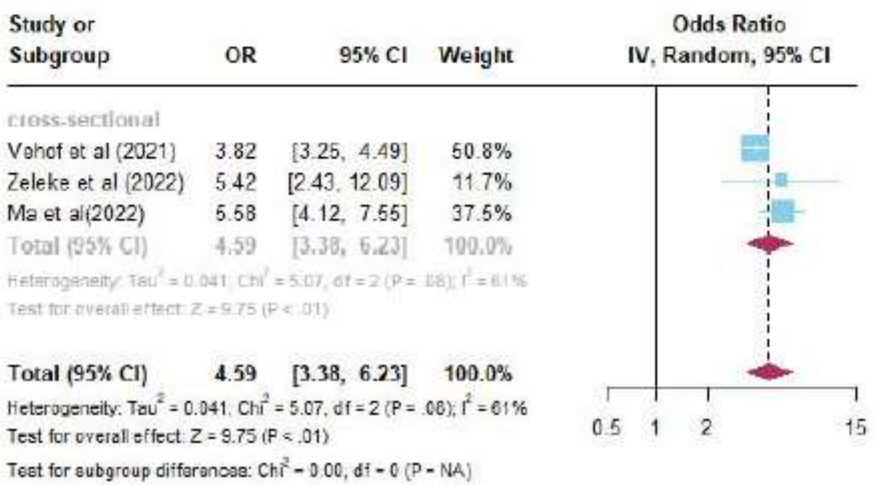

Forest Plot of Pterygium

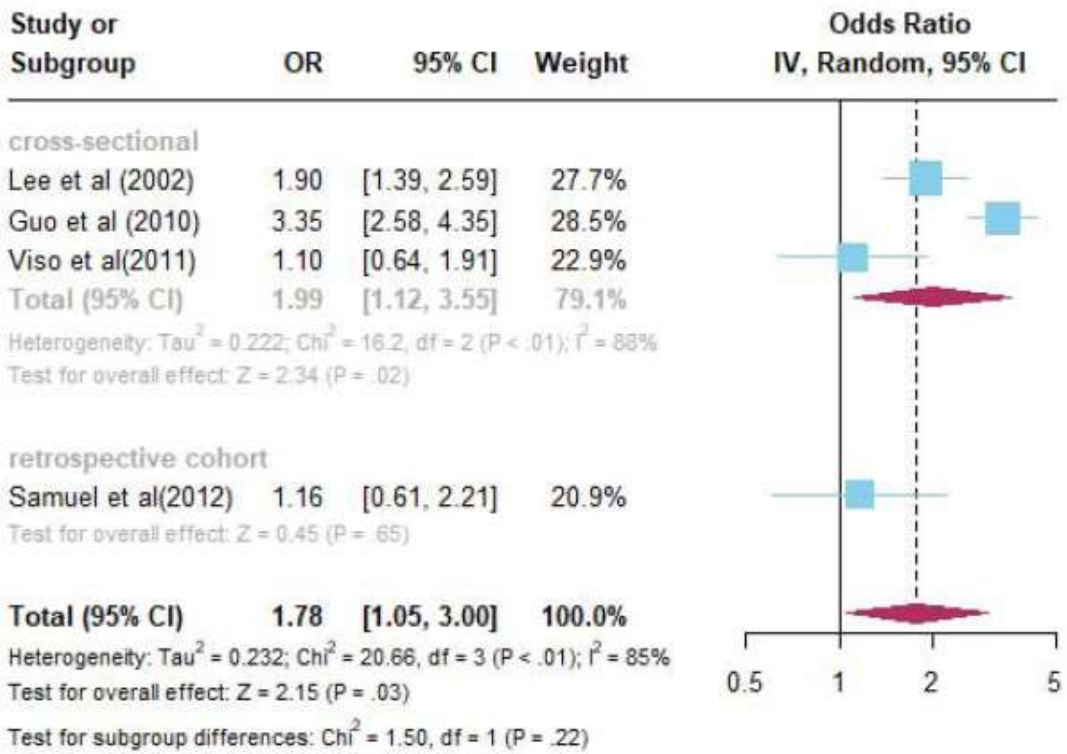

SUPPLEMENTAL DIGITAL CONTENT 11

SUPPLEMENTAL DIGITAL CONTENT 12

Forest Plot of Refractive Surgery

Study or Subgroup	OR	95% CI	Weight
cross-sectional			
Zhang et al (2019)	1.23	[1.15, 1.31]	25.9%
Yu et al (2019)	1.72	[1.59, 1.87]	25.8%
Vehof et al (2021)	3.51	[2.99, 4.12]	25.0%
Alkhaldi et al(2023)	1.79	[1.36, 2.35]	23.2%
Total (95% CI)	1.90	[1.28, 2.84]	100.0%
Heterogeneity: $Tau^2 = 0.158$; $Chi^2 = 157.06$, df = 3 ($P < .01$); $I^2 = 98\%$			
Test for overall effect: $Z = 3.17$ ($P < .01$)			
**Total (95% CI)**	**1.90**	**[1.28, 2.84]**	**100.0%**
Heterogeneity: $Tau^2 = 0.158$; $Chi^2 = 157.06$, df = 3 ($P < .01$); $I^2 = 98\%$			
Test for overall effect: $Z = 3.17$ ($P < .01$)			
Test for subgroup differences: $Chi^2 = 0.00$, df = 0 (P = NA)			

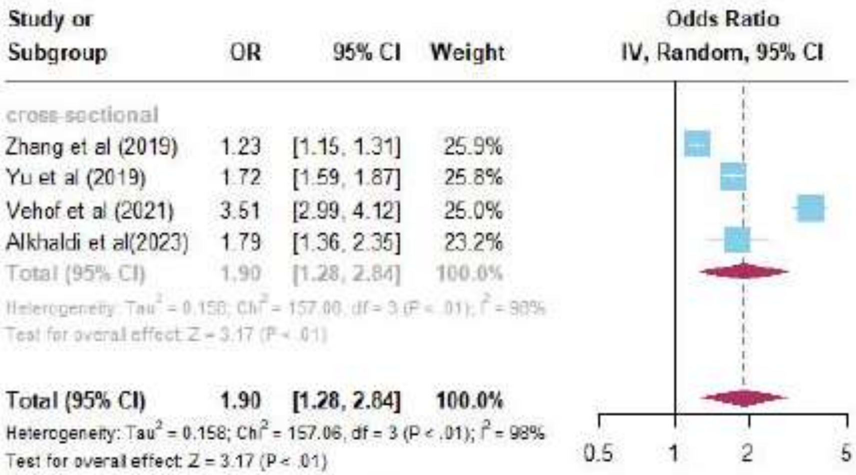

Forest Plot of Diabetes

Study or Subgroup	OR	95% CI	Weight
case-control			
Galor et al (2012)	1.06	[1.06, 1.06]	16.8%
Yang et al (2015)	1.41	[1.03, 1.92]	3.8%
Total (95% CI)	1.17	[0.90, 1.52]	20.6%
Heterogeneity: Tau^2 = 0.028; Chi^2 = 3.18, df = 1 (P = .07); I^2 = 69%			
Test for overall effect: Z = 1.15 (P = .25)			
cross-sectional			
Chia et al(2003)	1.10	[0.62, 1.96]	1.3%
Schaumberg et al (2009)	0.97	[0.75, 1.26]	5.0%
Viso et al (2009)	1.22	[0.61, 2.44]	0.9%
Vehof et al(2014)	1.53	[0.99, 2.37]	2.2%
Yoon et al(2016)	1.00	[0.86, 1.15]	9.6%
Alshamrani et al (2017)	1.51	[1.16, 1.96]	5.0%
Gong et al (2017)	1.10	[0.69, 1.76]	1.9%
Ferrero et al (2018)	0.97	[0.49, 1.91]	1.0%
Zhang et al (2019)	0.99	[0.91, 1.08]	13.3%
Inomata et al (2019)	1.39	[0.82, 2.36]	1.6%
Kim et al (2019)	1.08	[0.82, 1.41]	4.7%
Shanti et al (2020)	1.05	[0.67, 1.64]	2.1%
Tandon et al (2020)	1.20	[0.98, 1.47]	6.9%
Inomata et al(2020)	0.82	[0.42, 1.62]	1.0%
Vehof et al (2021)	1.29	[1.14, 1.45]	11.2%
Choi et al (2021)	1.05	[0.59, 1.86]	1.3%
Yang et al (2021)	0.96	[0.30, 3.12]	0.3%
Wu et al (2021)	0.86	[0.59, 1.24]	2.9%
Khorshed et al (2022)	3.70	[1.42, 9.65]	0.5%
Dossari et al(2022)	1.35	[0.79, 2.30]	1.5%
Garg et al(2022)	1.93	[0.99, 3.77]	1.0%
Total (95% CI)	1.14	[1.04, 1.26]	75.3%
Heterogeneity: Tau^2 = 0.015; Chi^2 = 38.95, df = 20 (P = .01); I^2 = 40%			
Test for overall effect: Z = 2.77 (P < .01)			
prospective cohort			
Moss et al (2000)	1.38	[1.03, 1.85]	4.1%
Test for overall effect: Z = 2.14 (P = .03)			
**Total (95% CI)**	**1.14**	**[1.06, 1.22]**	**100.0%**
Heterogeneity: Tau^2 = 0.007; Chi^2 = 45.02, df = 23 (P < .01); I^2 = 49%			
Test for overall effect: Z = 3.68 (P < .01)			
Test for subgroup differences: Chi^2 = 1.43, df = 2 (P = .49)			

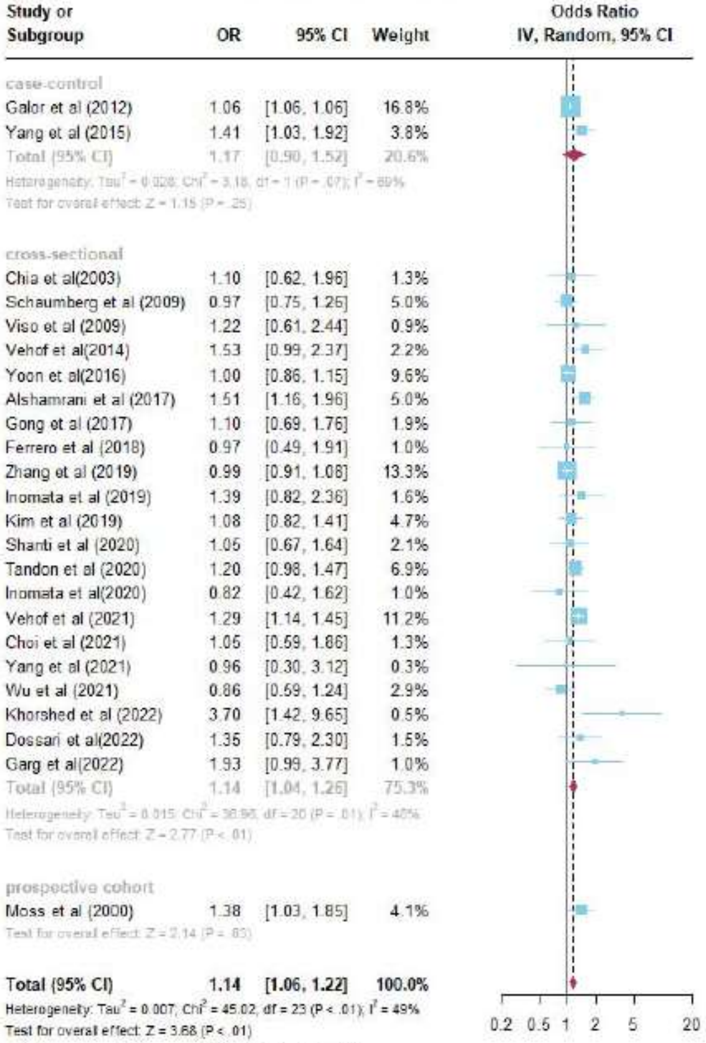

SUPPLEMENTAL DIGITAL CONTENT 13

Forest Plot of Thyroid Disease

Study or Subgroup	OR	95% CI	Weight
case-control			
Galor et al(2011)	1.48	[0.74, 2.96]	2.7%
Galor et al (2012)	1.81	[1.79, 1.84]	6.8%
Total (95% CI)	1.81	[1.78, 1.84]	9.5%
Heterogeneity: $Tau^2 = 0$; $Chi^2 = 0.32$, df = 1 (P = .57); $I^2 = 0\%$			
Test for overall effect: Z = 33.96 (P = 0)			
cross-sectional			
Wang et al (2012)	1.94	[1.79, 2.10]	6.6%
Malet et al (2013)	1.18	[0.61, 2.28]	2.9%
Vehof et al(2014)	1.61	[1.22, 2.12]	5.5%
Paulsen et al (2014)	1.62	[1.20, 2.18]	5.3%
Ahn et al (2014)	1.81	[1.45, 2.25]	5.9%
Yoon et al(2016)	1.05	[1.00, 1.09]	6.7%
Roh et al (2016)	1.79	[1.41, 2.28]	5.7%
Roh et al(2016)	1.79	[1.41, 2.28]	5.7%
Alshamrani et al (2017)	1.41	[1.09, 1.83]	5.6%
Zhang et al (2019)	1.41	[1.27, 1.56]	6.5%
Yu et al (2019)	1.28	[1.14, 1.45]	6.5%
Kim et al (2019)	1.44	[0.96, 2.14]	4.5%
Wang et al (2021)	5.15	[1.69, 15.72]	1.4%
Bikbov et al(2022)	1.63	[1.19, 2.24]	5.1%
Garcia-Marqués et al (2022)	0.35	[0.06, 2.15]	0.6%
Dossari et al(2022)	4.53	[1.04, 19.73]	0.9%
Garcia-Queiruga et al(2023)	1.50	[1.11, 2.02]	5.3%
Alkhaldi et al(2023)	1.42	[0.84, 2.41]	3.6%
Total (95% CI)	1.55	[1.32, 1.82]	84.2%
Heterogeneity: $Tau^2 = 0.086$; $Chi^2 = 231.65$, df = 17 (P < .01); $I^2 = 93\%$			
Test for overall effect: Z = 5.26 (P < .01)			
prospective cohort			
Moss et al (2008)	1.73	[1.50, 2.00]	6.3%
Test for overall effect: Z = 7.47 (P < .01)			
**Total (95% CI)**	**1.57**	**[1.36, 1.82]**	**100.0%**

Heterogeneity: $Tau^2 = 0.083$; $Chi^2 = 594.32$, df = 20 (P < .01); $I^2 = 97\%$

Test for overall effect: Z = 6.05 (P < .01)

Test for subgroup differences: $Chi^2 = 3.89$, df = 2 (P = .14)

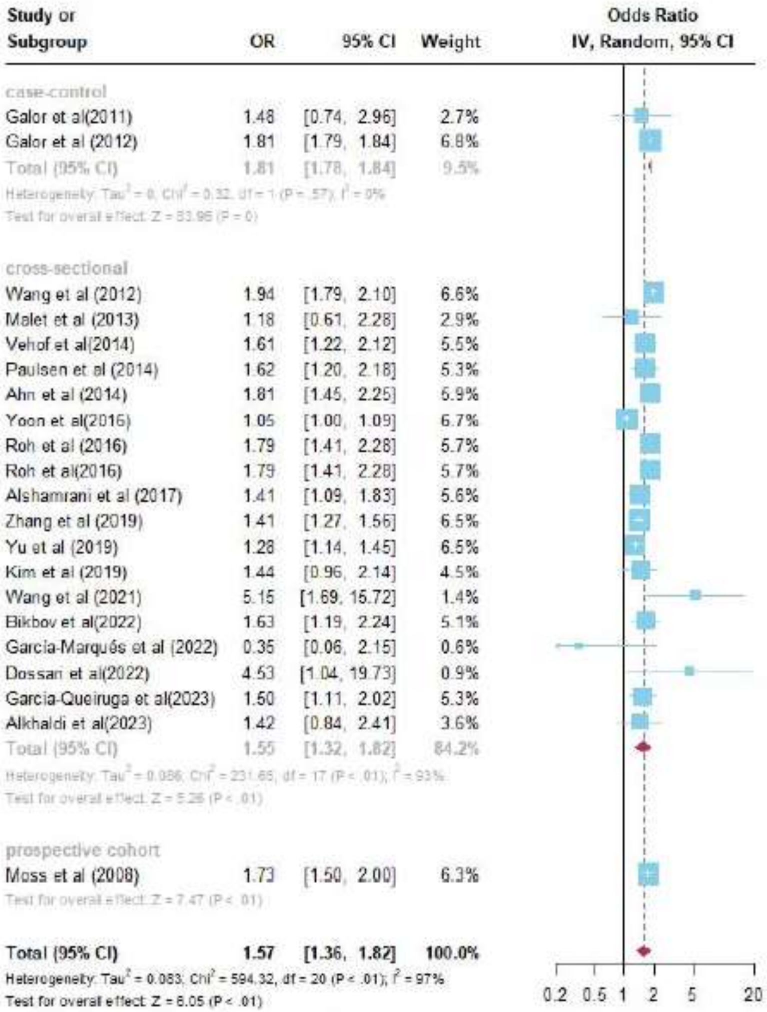

SUPPLEMENTAL DIGITAL CONTENT 14

SUPPLEMENTAL DIGITAL CONTENT 15

Forest Plot of Viral Infection

Study or Subgroup	OR	95% CI	Weight
case-control			
Galor et al(2011)	1.52	[1.15, 2.00]	16.6%
Galor et al (2012)	1.41	[1.35, 1.47]	41.1%
Yang et al (2015)	3.33	[1.63, 6.78]	3.8%
Total (95% CI)	1.59	[1.22, 2.08]	61.5%
Heterogeneity: $Tau^2 = 0.034$; $Chi^2 = 5.82$, df = 2 (P = .05); $I^2 = 66\%$			
Test for overall effect: Z = 3.42 (P < .01)			
cross-sectional			
Wang et al (2012)	1.64	[1.49, 1.81]	35.8%
Vehof et al (2021)	0.96	[0.41, 2.25]	2.7%
Total (95% CI)	1.49	[1.00, 2.23]	38.5%
Heterogeneity: $Tau^2 = 0.048$; $Chi^2 = 1.5$, df = 1 (P = .22); $I^2 = 33\%$			
Test for overall effect: Z = 1.97 (P = .05)			
**Total (95% CI)**	**1.54**	**[1.33, 1.78]**	**100.0%**
Heterogeneity: $Tau^2 = 0.013$; $Chi^2 = 14.05$, df = 4 (P < .01); $I^2 = 72\%$			
Test for overall effect: Z = 5.86 (P < .01)			
Test for subgroup differences: $Chi^2 = 0.07$, df = 1 (P = .79)			

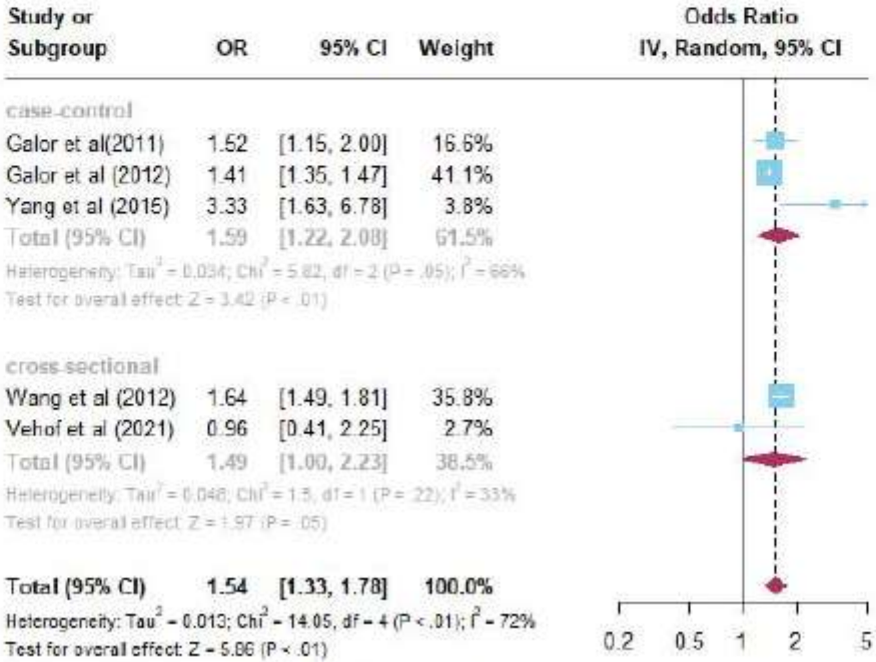

SUPPLEMTENTAL DIGITAL CONTENT 16

Forest Plot of Betablockers

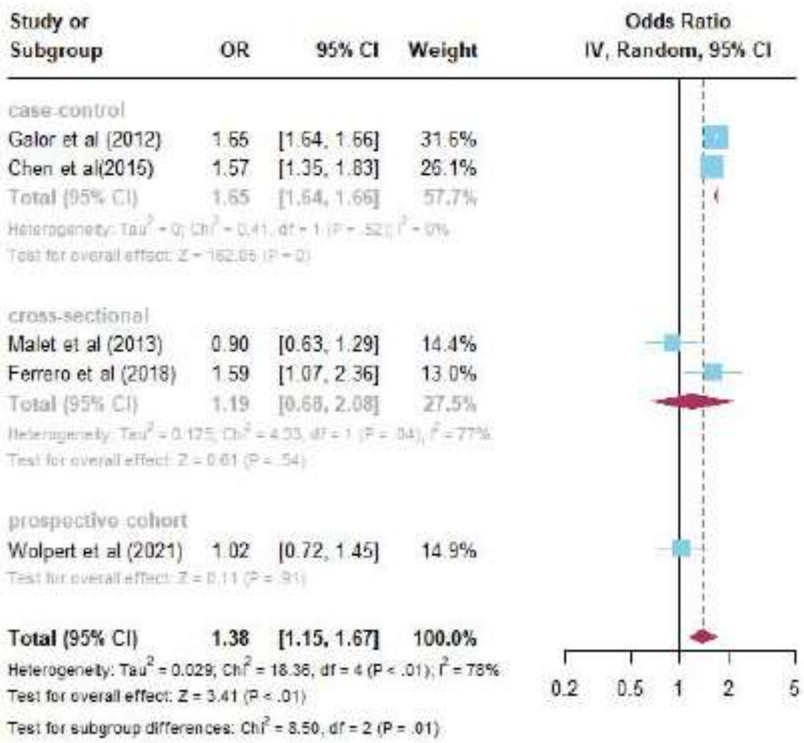

SUPPLEMENTAL DIGITAL CONTENT 17

Forest Plot of Diuretics

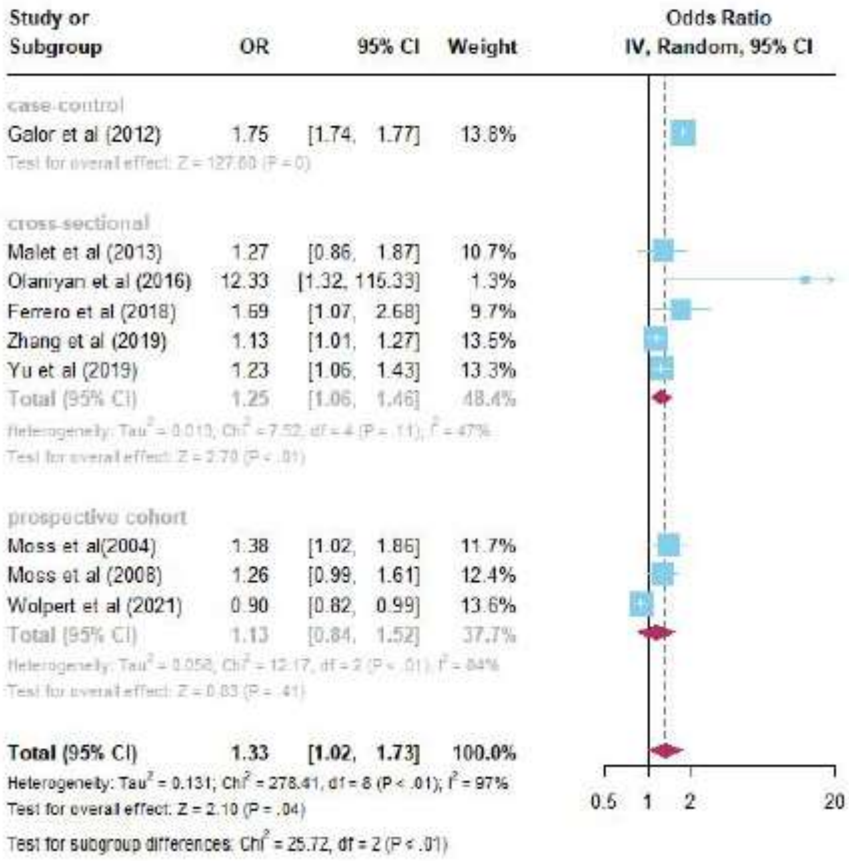

SUPPLEMENTAL DIGITAL CONTENT 18

Forest Plot of Multivitamins

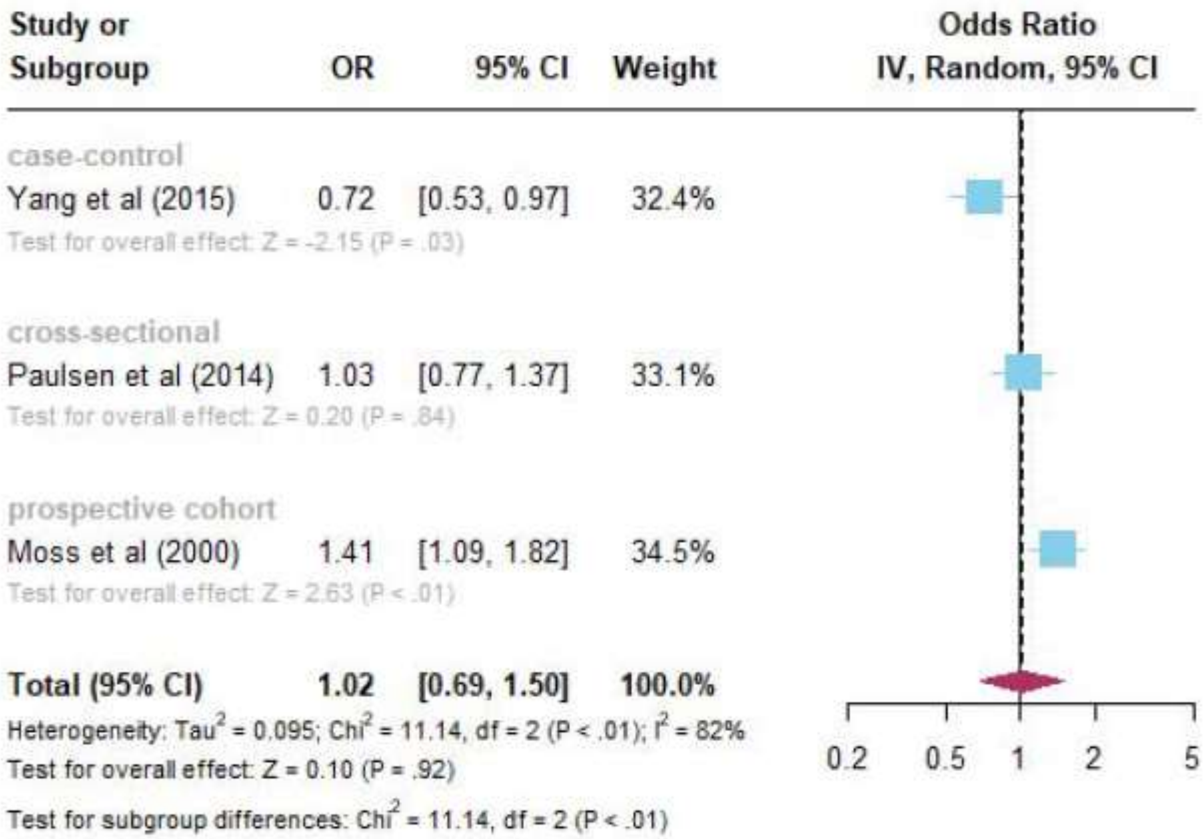

SUPPLEMENTAL DIGITAL CONTENT 19

Forest Plot of Oral Contraceptives

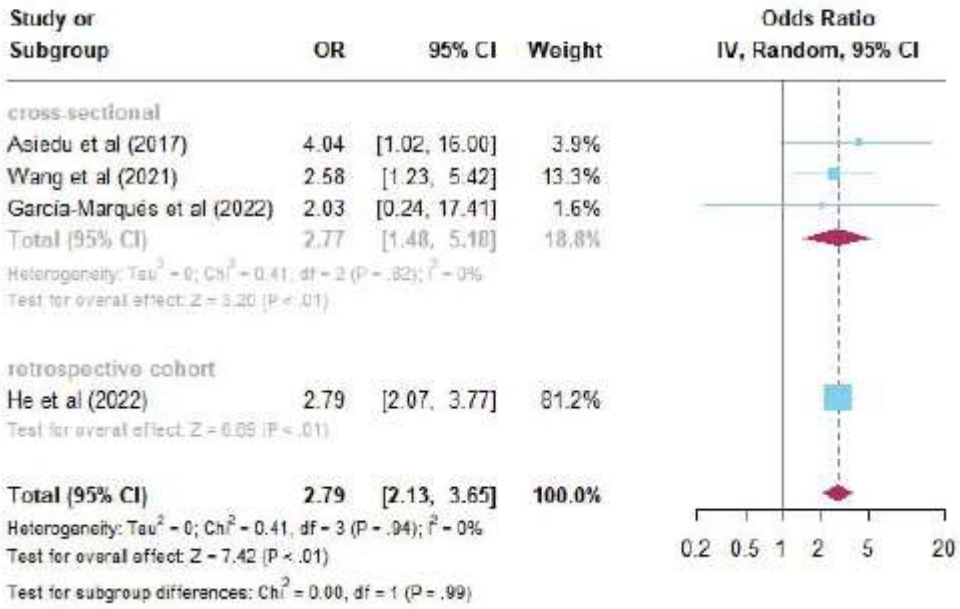

SUPPLEMENTAL DIGITAL CONTENT 20

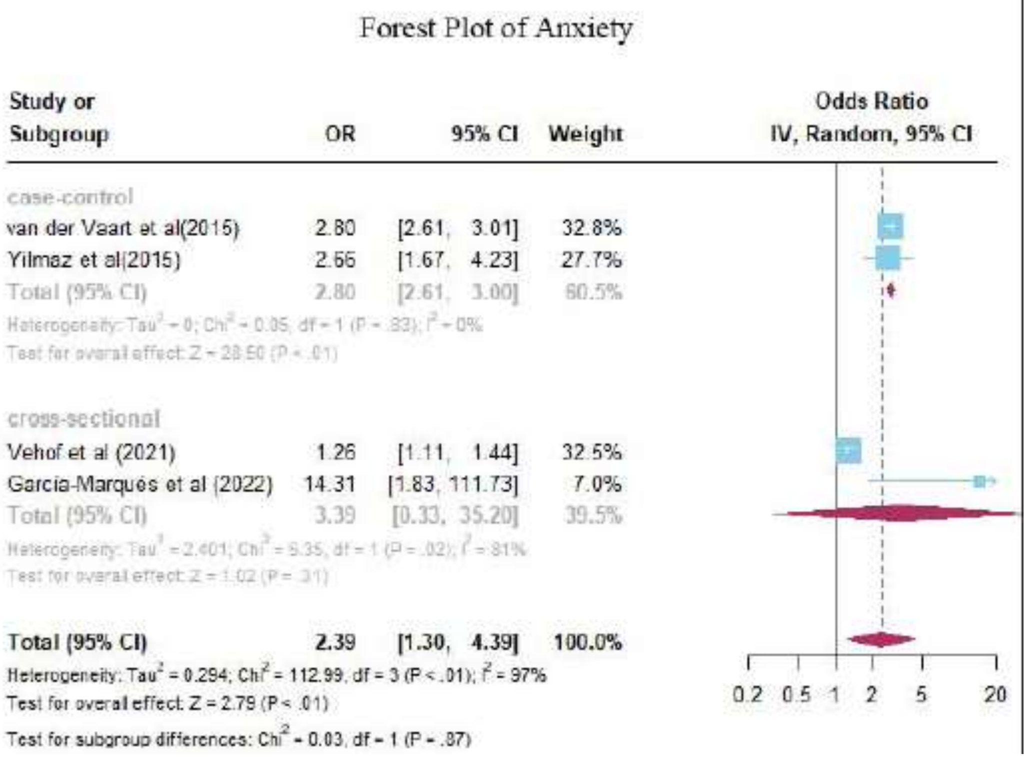

Forest Plot of Depression

Study or Subgroup	OR	95% CI	Weight
case-control			
Galor et al(2011)	1.91	[1.73, 2.10]	6.7%
Galor et al (2012)	1.34	[1.33, 1.35]	6.8%
Hallak et al(2015)	2.86	[1.04, 7.87]	2.3%
van der Vaart et al(2015)	2.90	[2.71, 3.11]	6.8%
Yilmaz et al(2015)	2.06	[1.30, 3.27]	4.8%
Total (95% CI)	2.02	[1.47, 2.78]	27.4%
Heterogeneity: $Tau^2 = 0.103$; $Chi^2 = 528.18$, df = 4 ($P < .01$); $I^2 = 99\%$			
Test for overall effect: $Z = 4.33$ ($P < .01$)			
cross-sectional			
Wang et al (2012)	2.11	[1.93, 2.31]	6.7%
Fernandez et al(2013)	1.16	[0.46, 2.91]	2.6%
Vehof et al(2014)	1.67	[1.27, 2.19]	6.0%
Yoon et al(2016)	1.07	[1.03, 1.12]	6.8%
Ferrero et al (2018)	1.40	[0.82, 2.40]	4.4%
Inomata et al (2019)	1.68	[1.23, 2.29]	5.8%
Kim et al (2019)	1.21	[0.97, 1.52]	6.2%
Inomata et al (2020)	7.39	[3.70, 14.78]	3.5%
Inomata et al(2020)	0.90	[0.62, 1.31]	5.4%
Vehof et al (2021)	1.52	[1.42, 1.62]	6.8%
Garcia-Marquès et al (2022)	1.88	[0.22, 15.82]	0.7%
An et al (2022)	1.37	[1.06, 1.77]	6.1%
Bikbov et al(2022)	1.04	[1.01, 1.07]	6.8%
Cartes et al(2022)	1.57	[0.99, 2.48]	4.8%
Total (95% CI)	1.48	[1.20, 1.84]	72.6%
Heterogeneity: $Tau^2 = 0.120$; $Chi^2 = 347.80$, df = 13 ($P < .01$); $I^2 = 96\%$			
Test for overall effect: $Z = 3.60$ ($P < .01$)			
**Total (95% CI)**	**1.62**	**[1.34, 1.95]**	**100.0%**
Heterogeneity: $Tau^2 = 0.134$; $Chi^2 = 1086.56$, df = 18 ($P < .01$); $I^2 = 98\%$			
Test for overall effect: $Z = 5.04$ ($P < .01$)			
Test for subgroup differences: $Chi^2 = 2.49$, df = 1 ($P = .11$)			

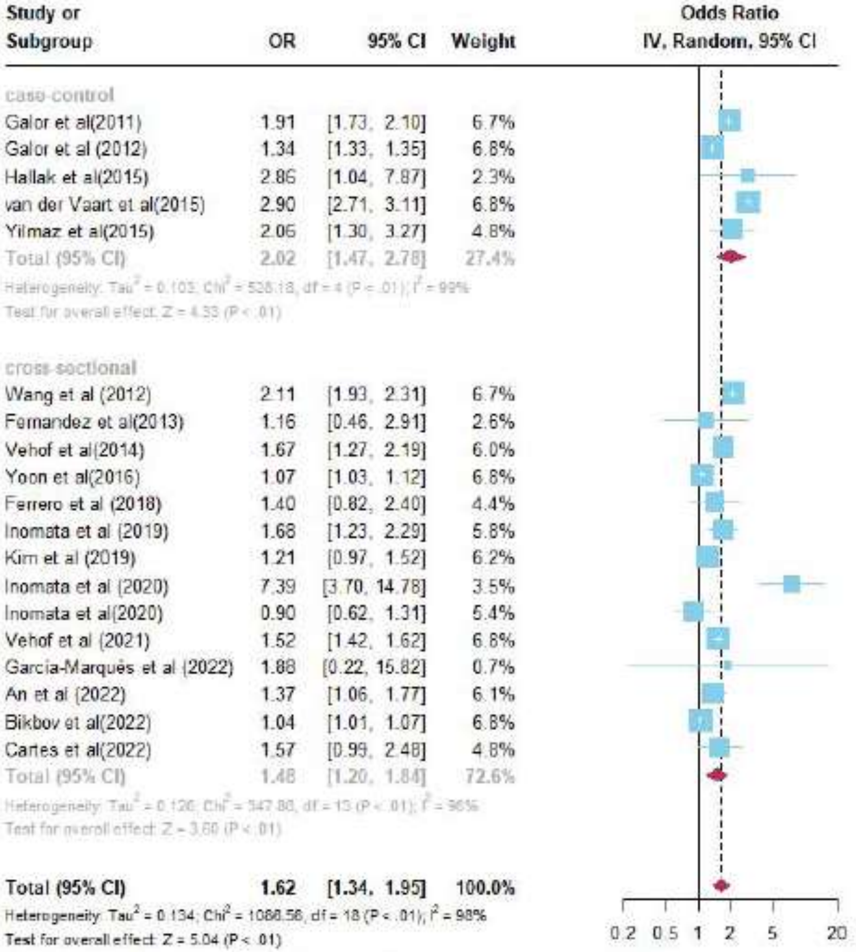

SUPPLEMENTAL DIGITAL CONTENT 21

SUPPLEMENTAL DIGITAL CONTENT 22

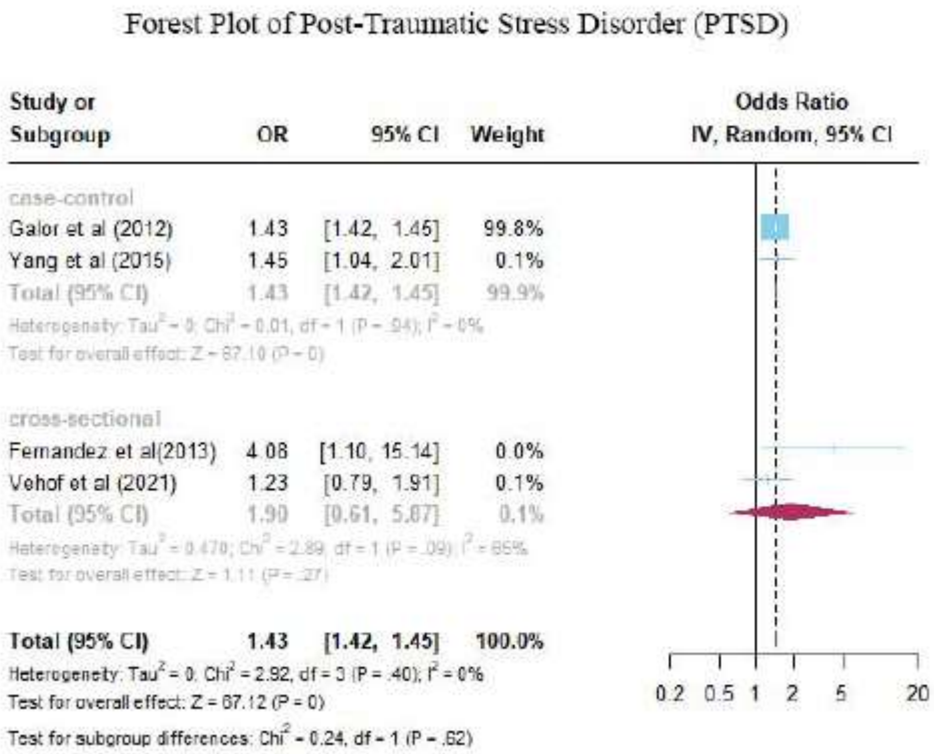

SUPPLEMENTAL DIGITAL CONTENT 23

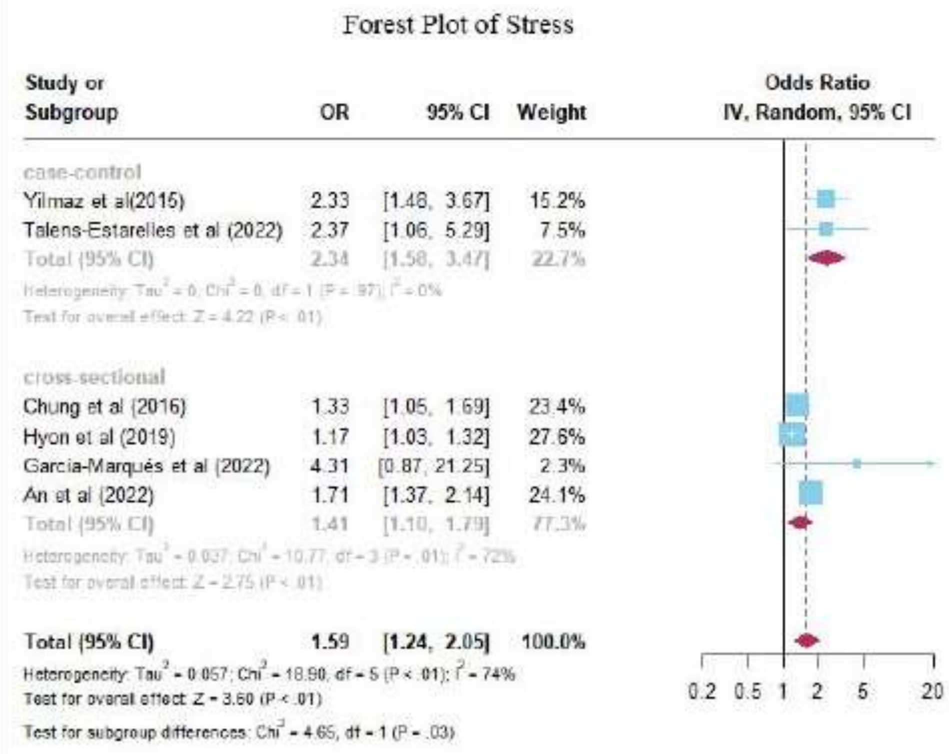

SUPPLEMENTAL DIGITAL CONTENT 24

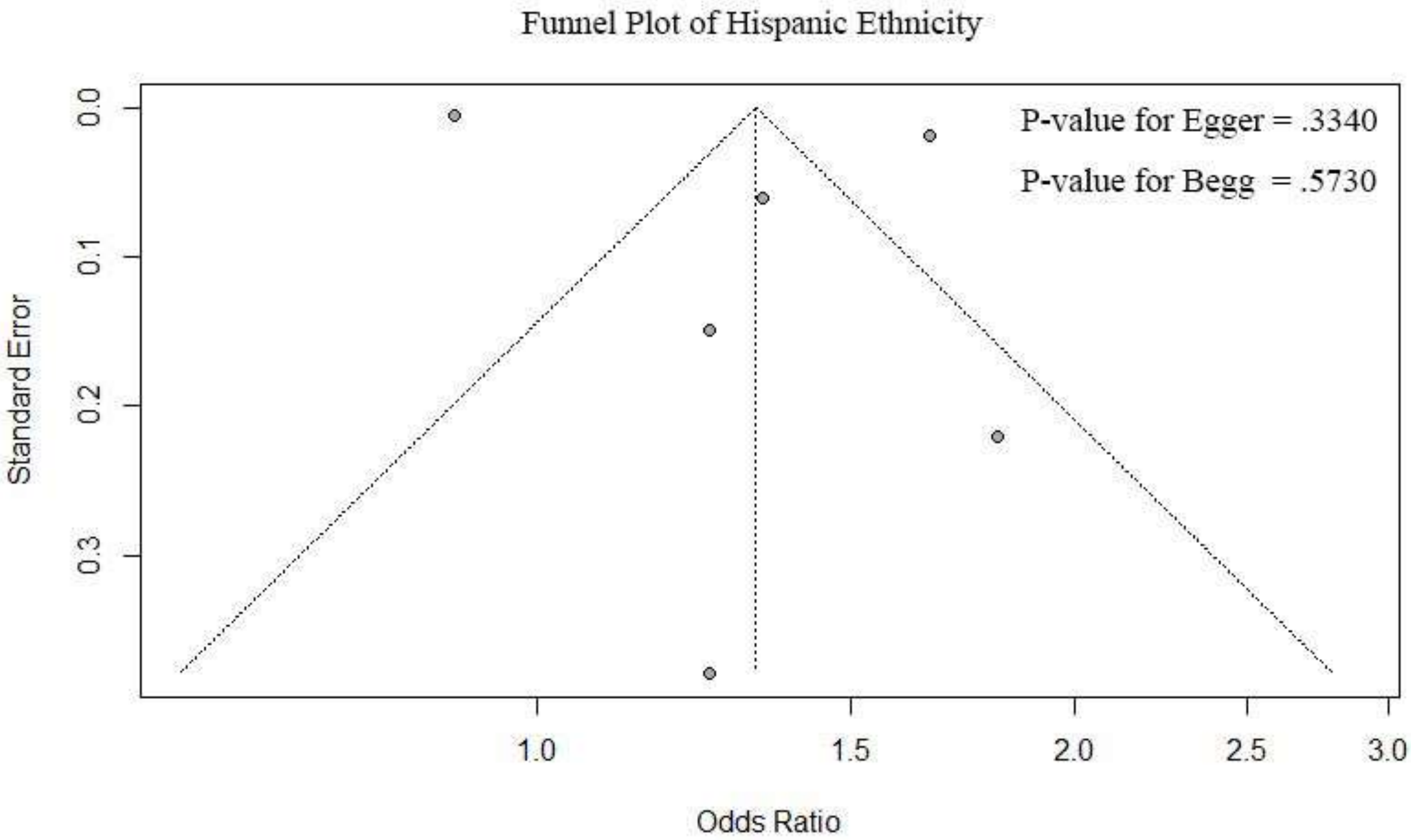

SUPPLEMENTAL DIGITAL CONTENT 25

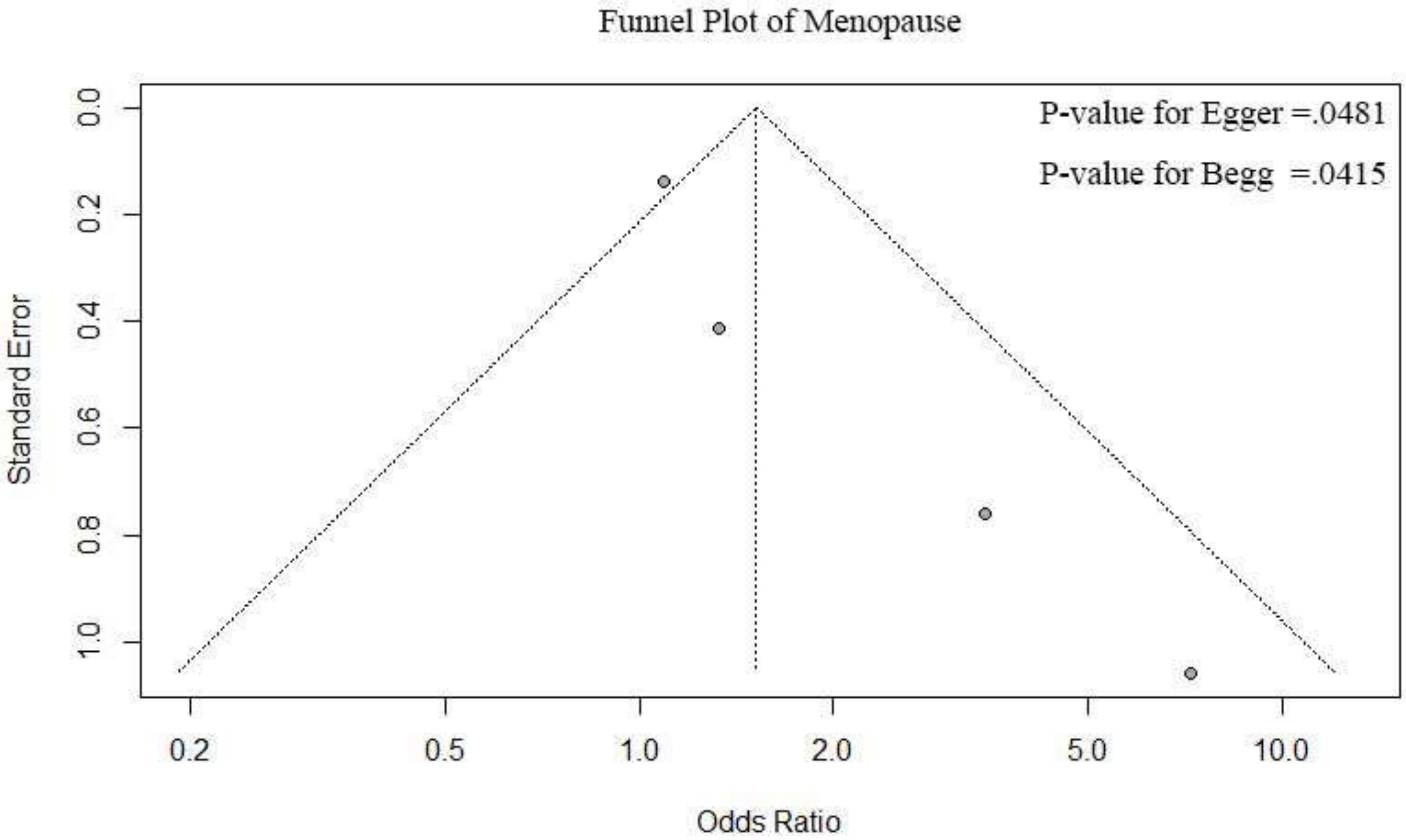

SUPPLEMENTAL DIGITAL CONTENT 26

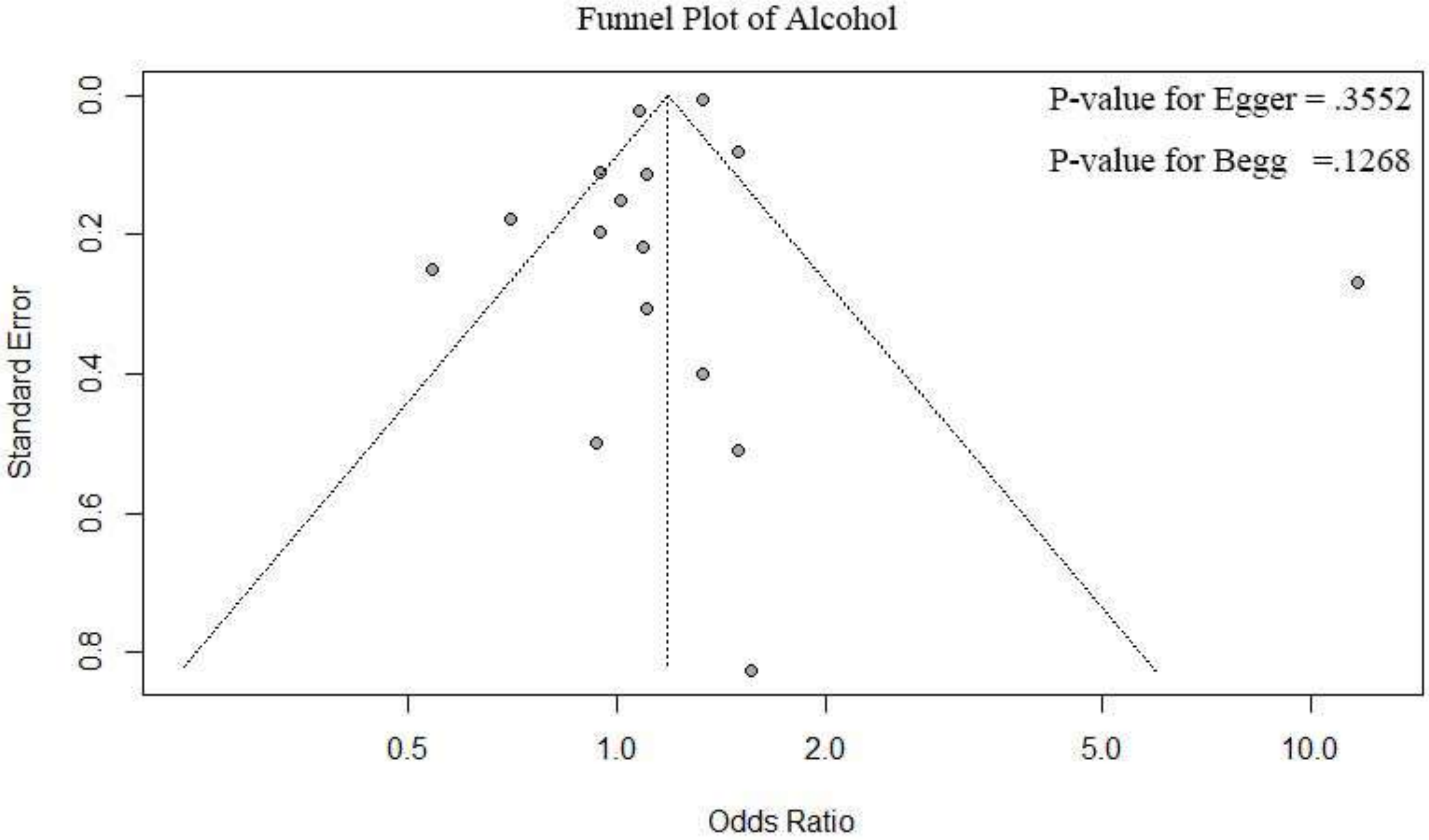

SUPPLEMENTAL DIGITAL CONTENT 27

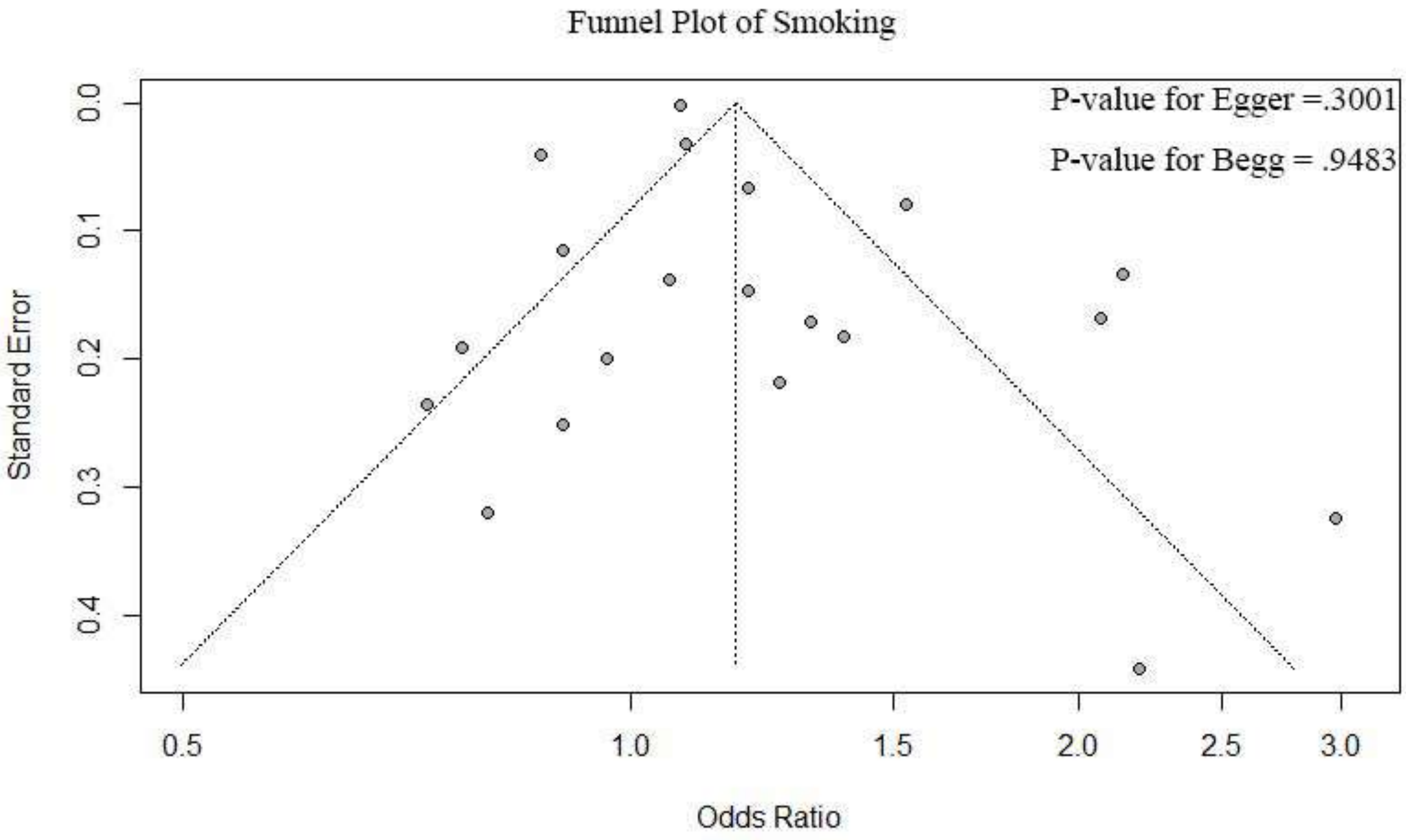

SUPPLEMTAL DIGITAL CONTENT 28

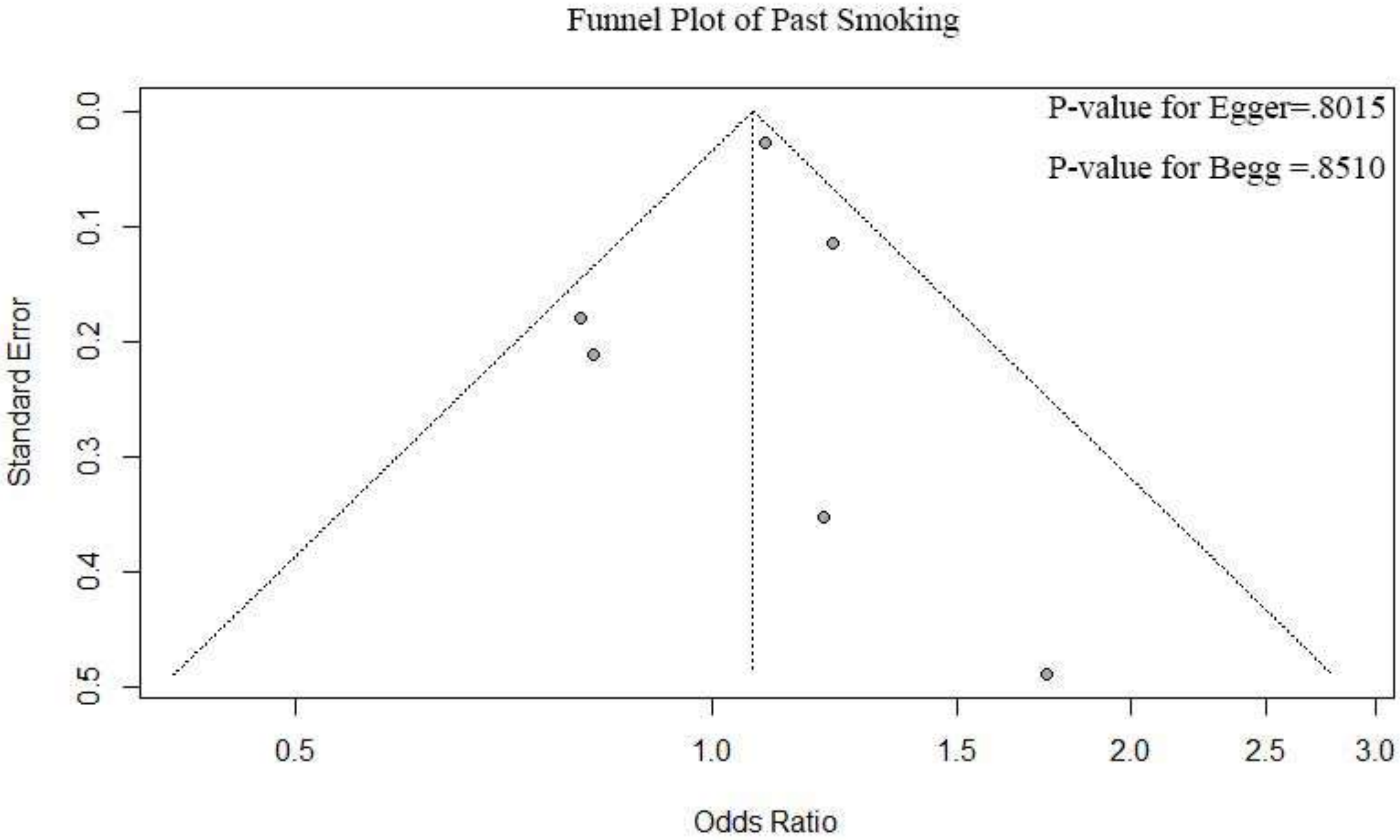

SUPPLEMENTAL DIGITAL CONTENT 29

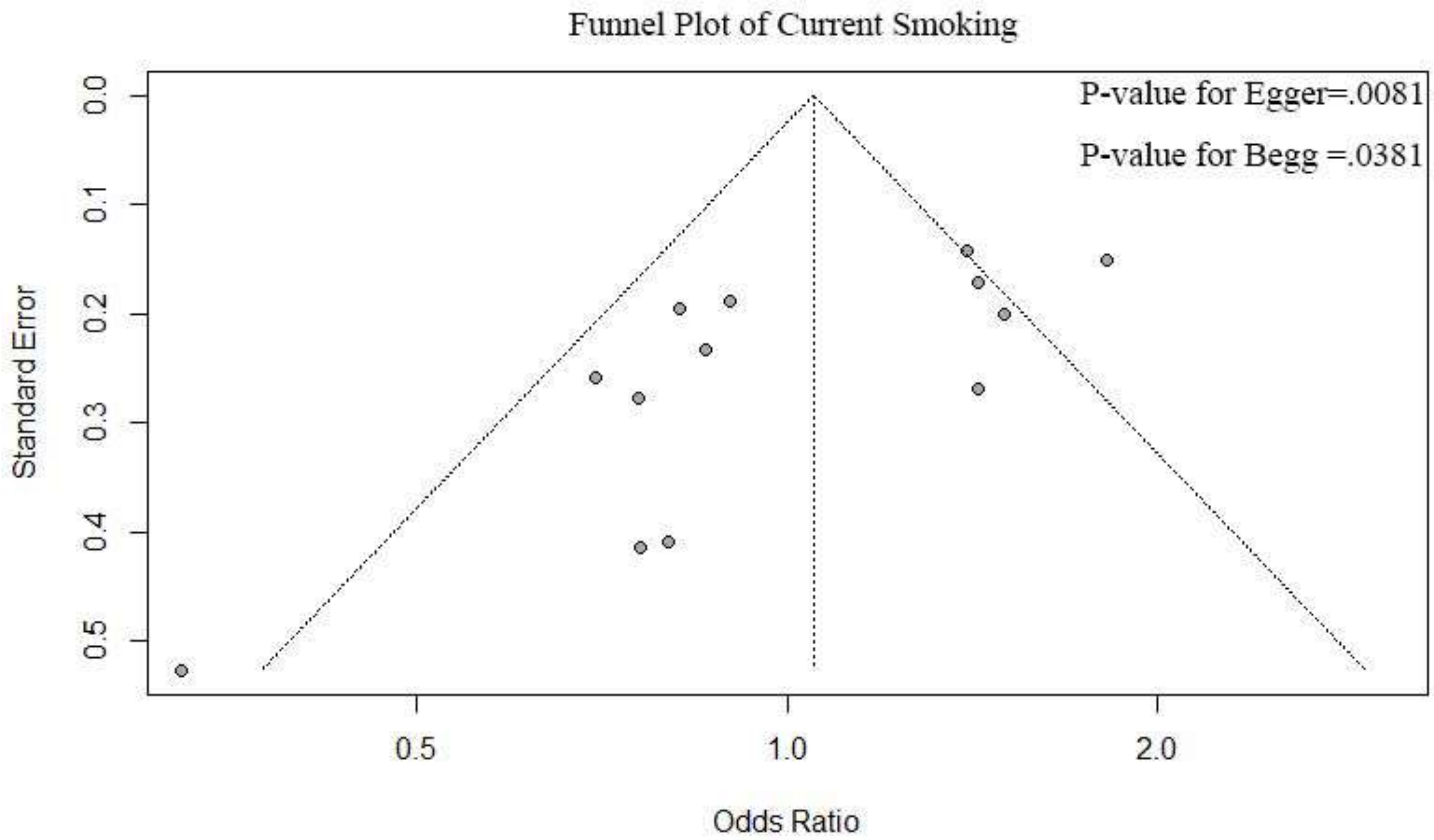

SUUPLEMENTAL DIGITAL CONTENT 30

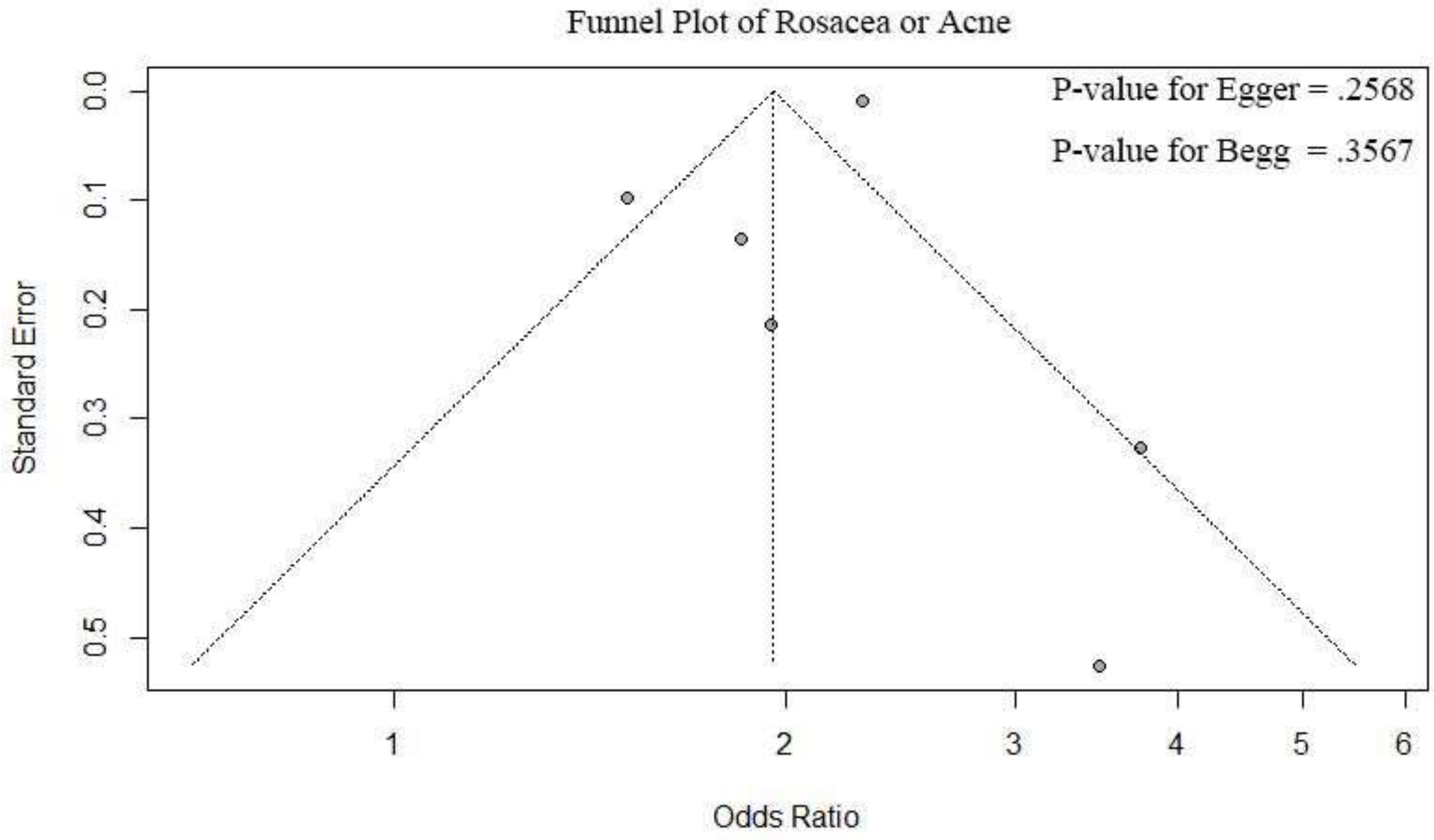

SUPPLEMENTAL DIGITAL CONTENT 31

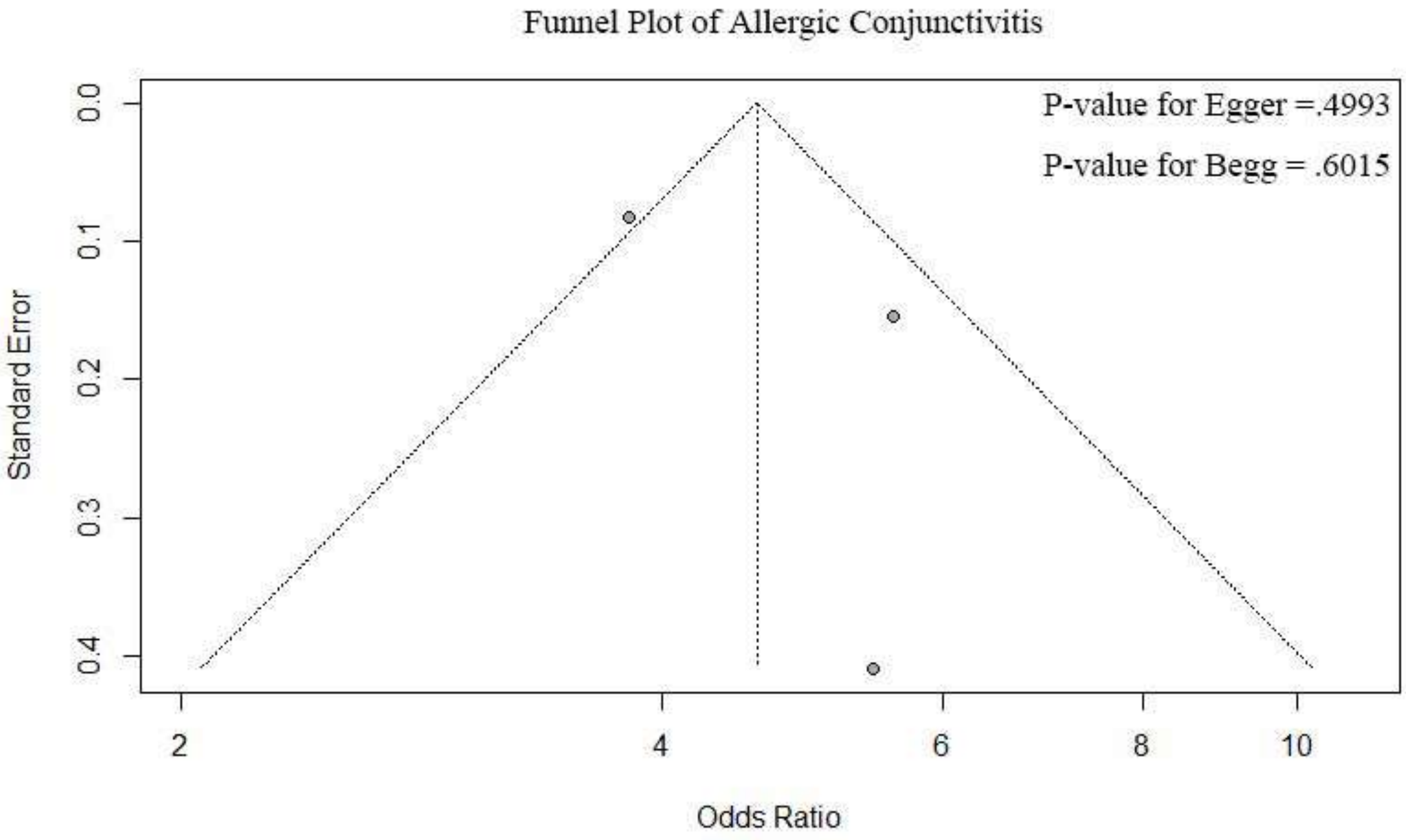

SUPPLEMENTAL DIGITAL CONTENT 32

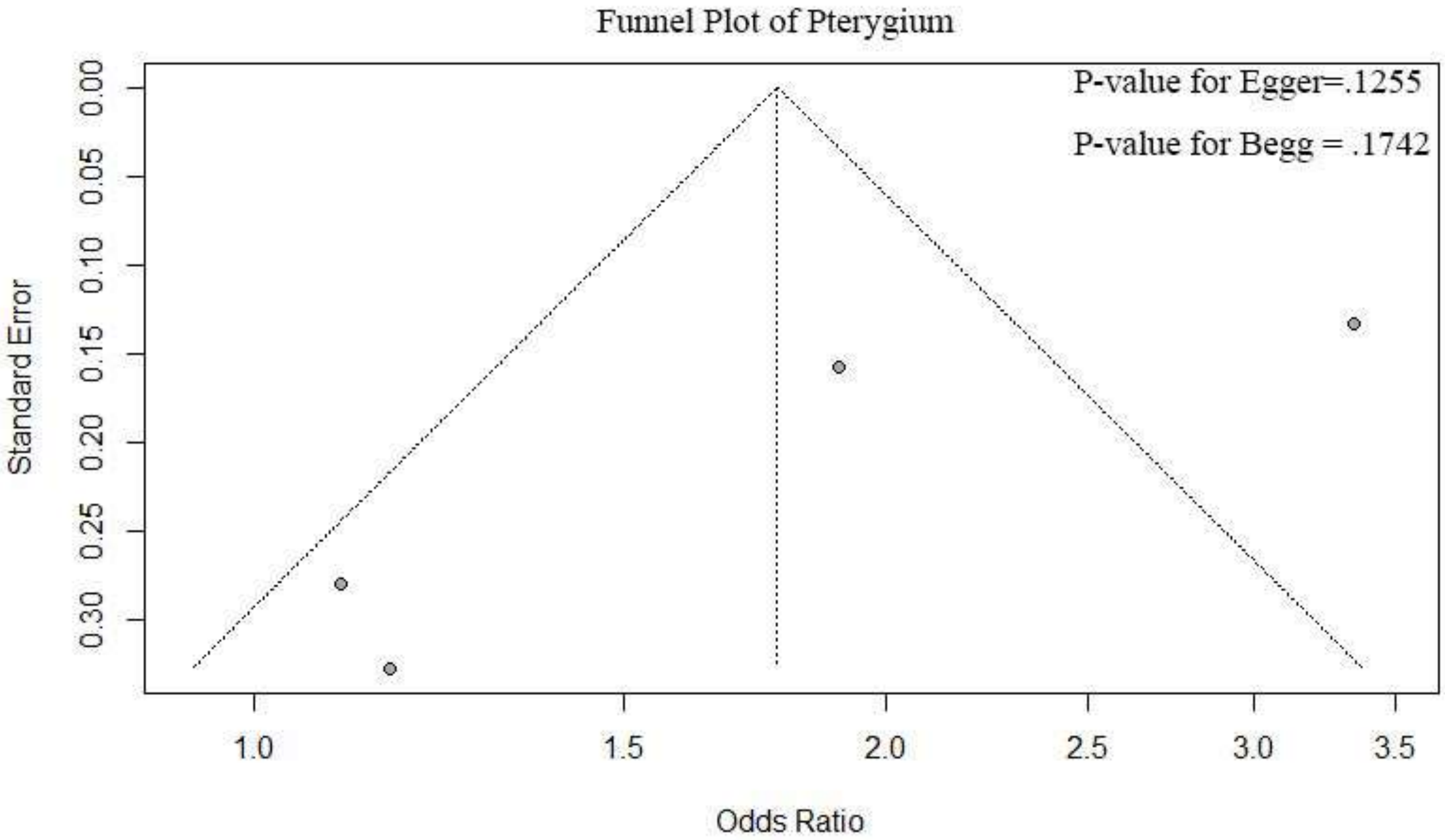

SUPPLEMENTAL DIGITAL CONTENT 33

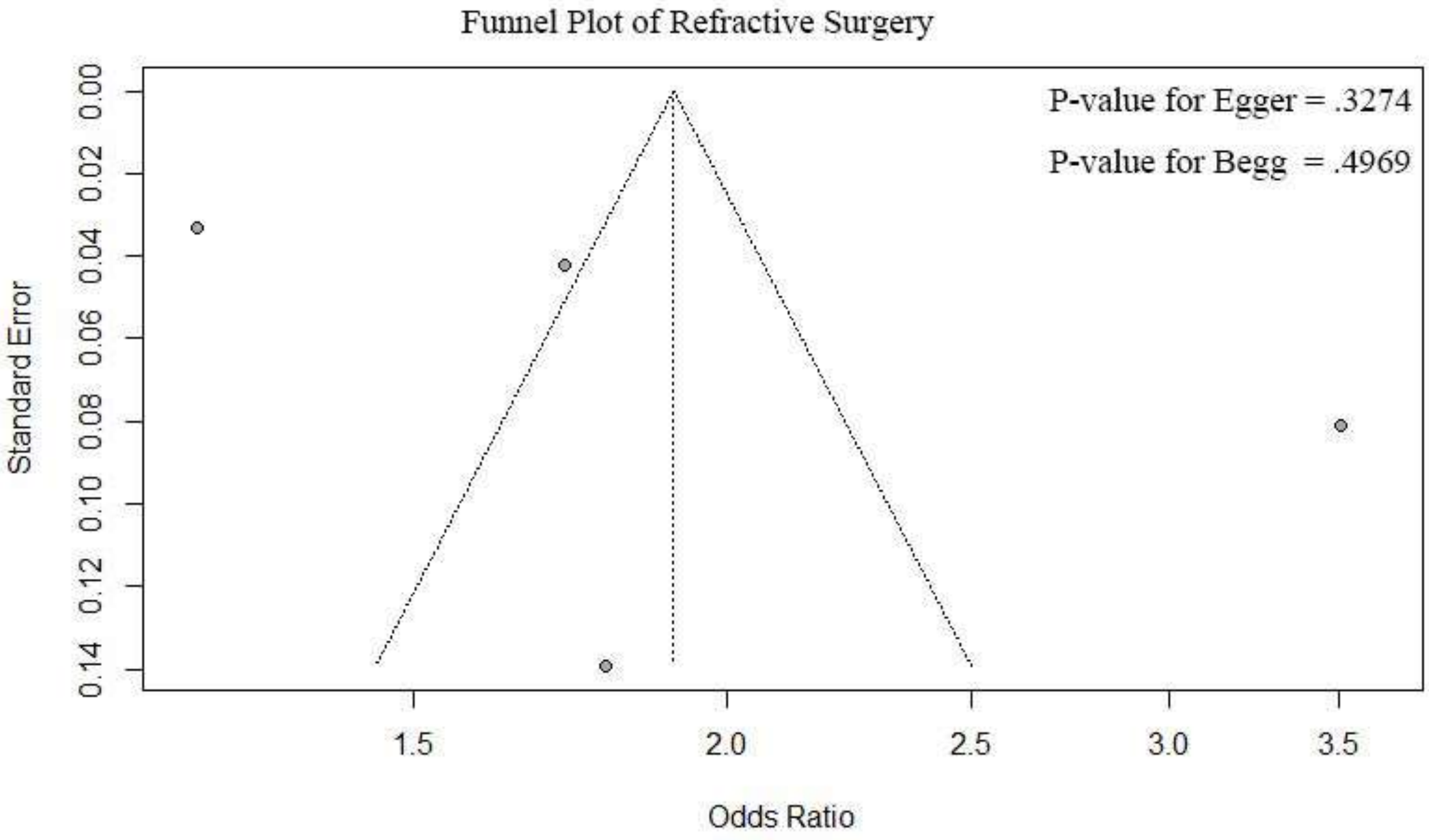

SUPPLEMENTAL DIGITAL CONTENT 34

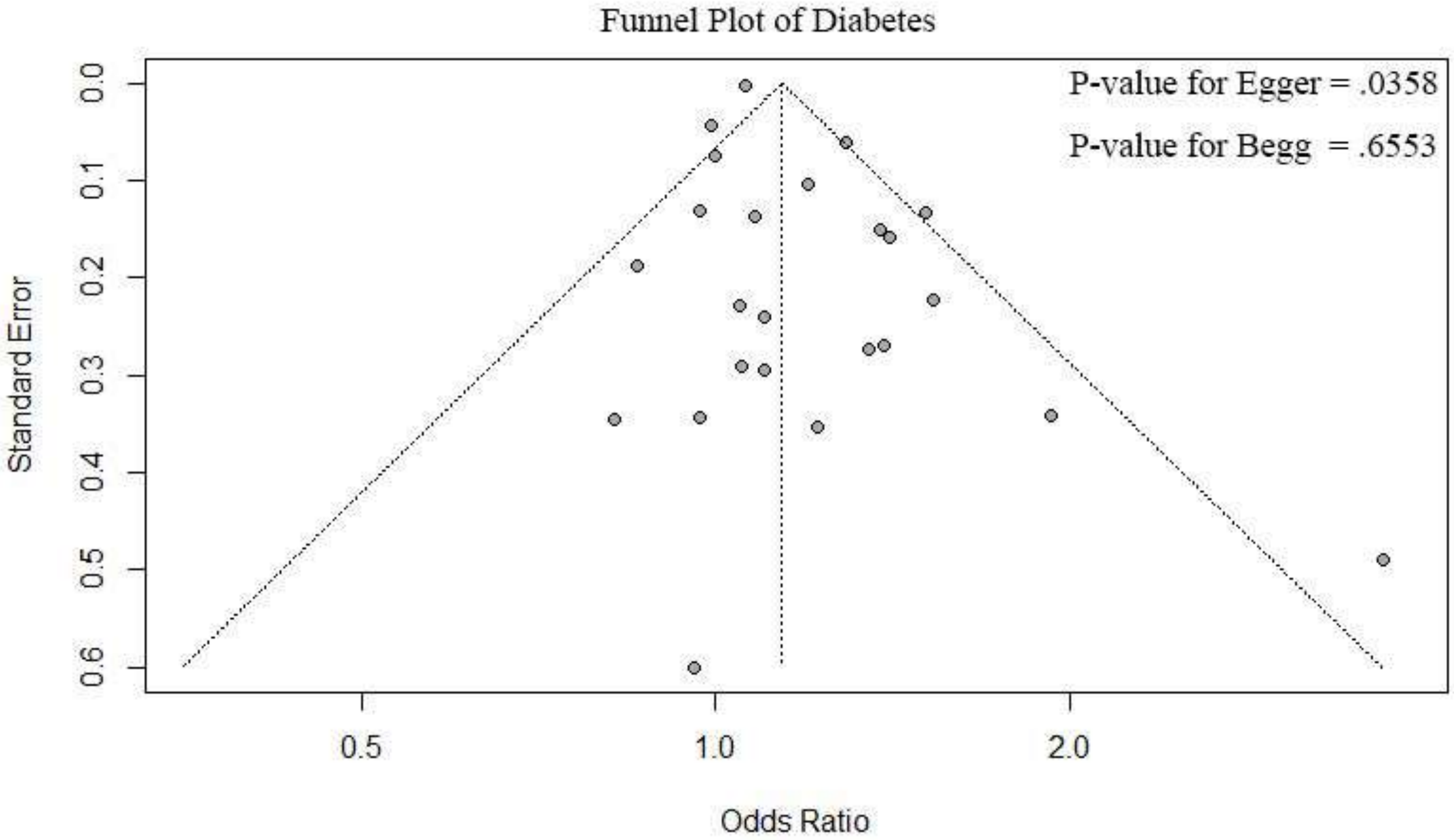

SUPPLEMENTAL DIGITAL CONTENT 35

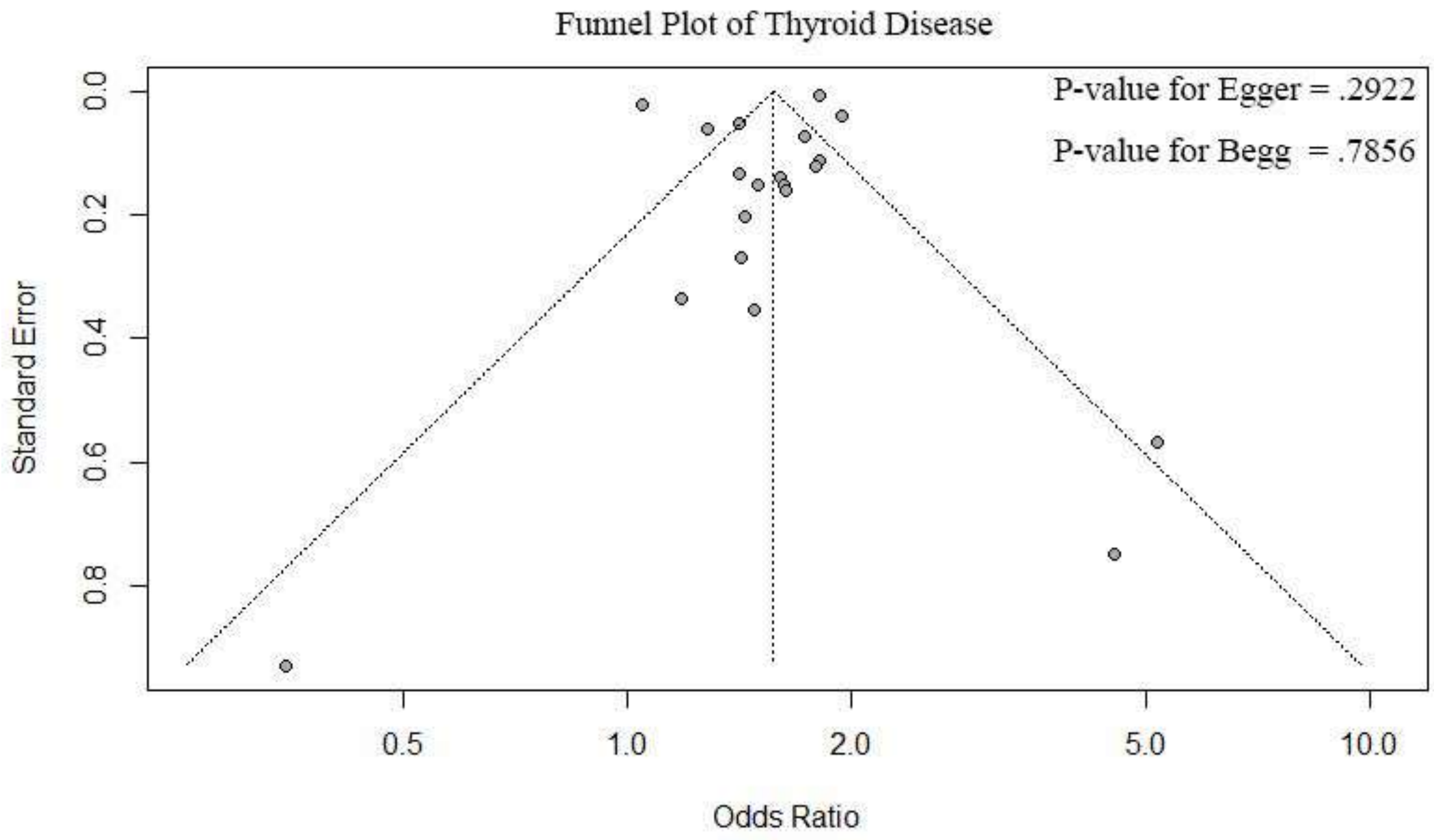

SUPPLEMENTAL DIGITAL CONTENT 36

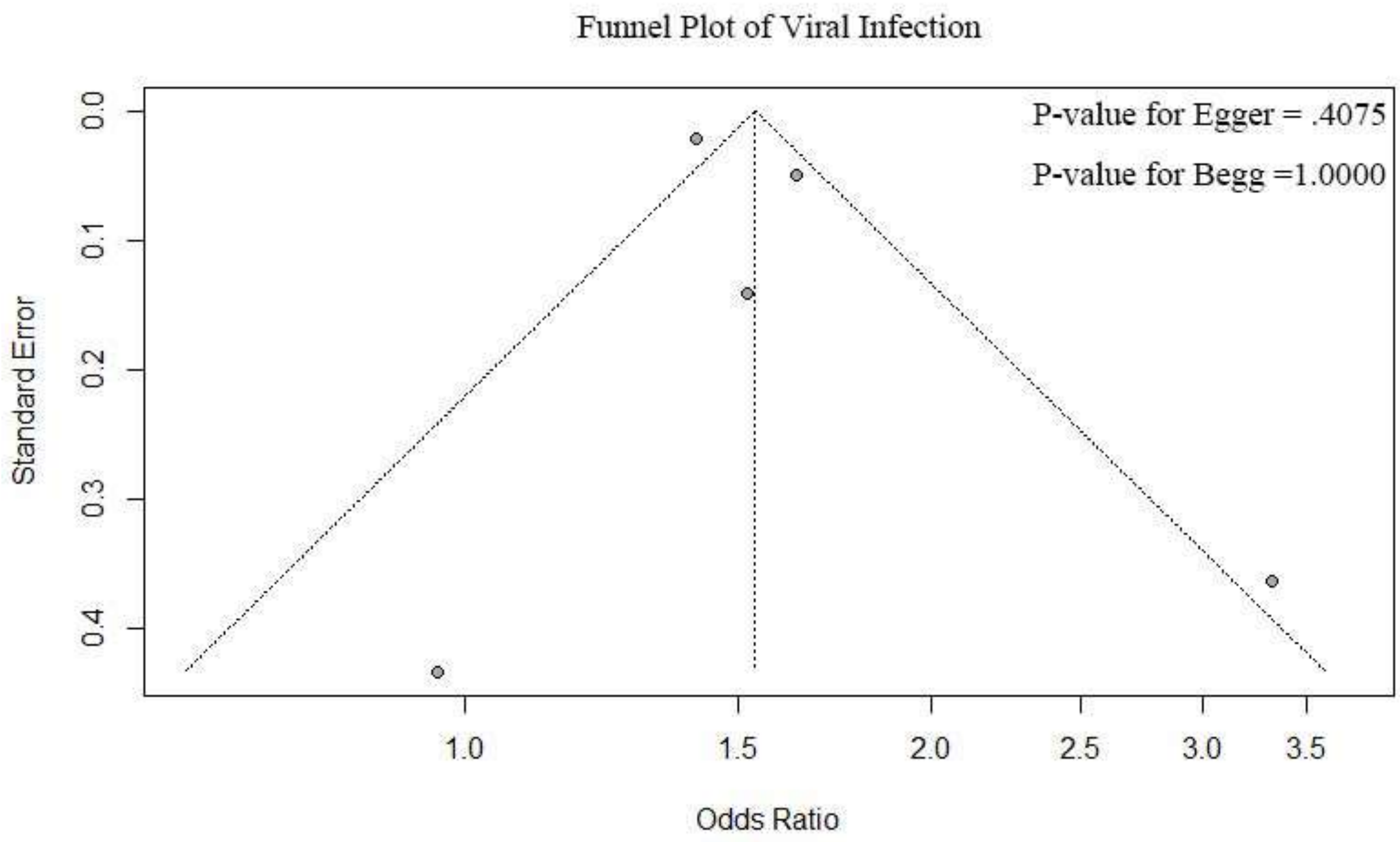

SUPPLEMENTAL DIGITAL CONTENT 37

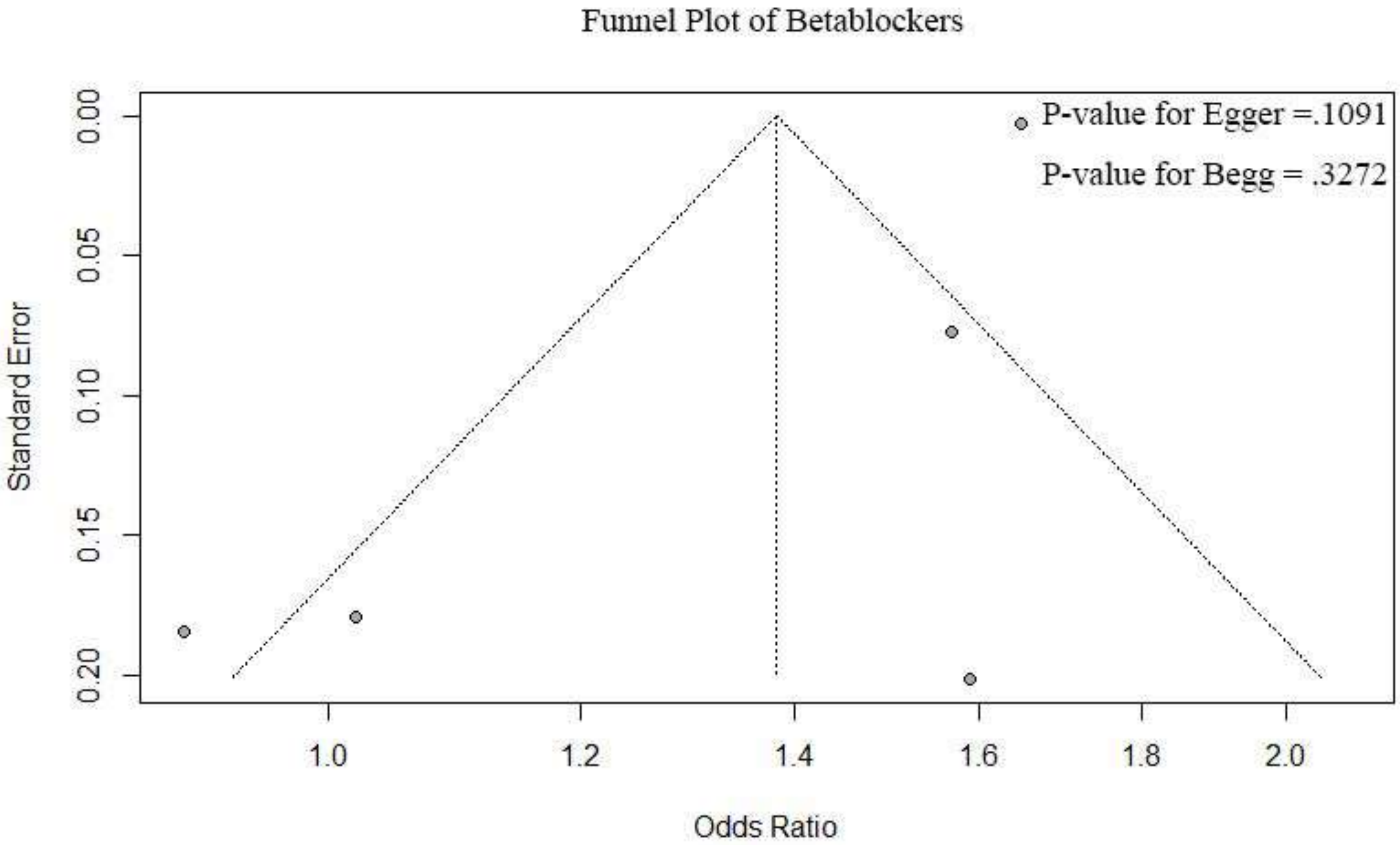

SUPPLEMENTAL DIGITAL CONTENT 38

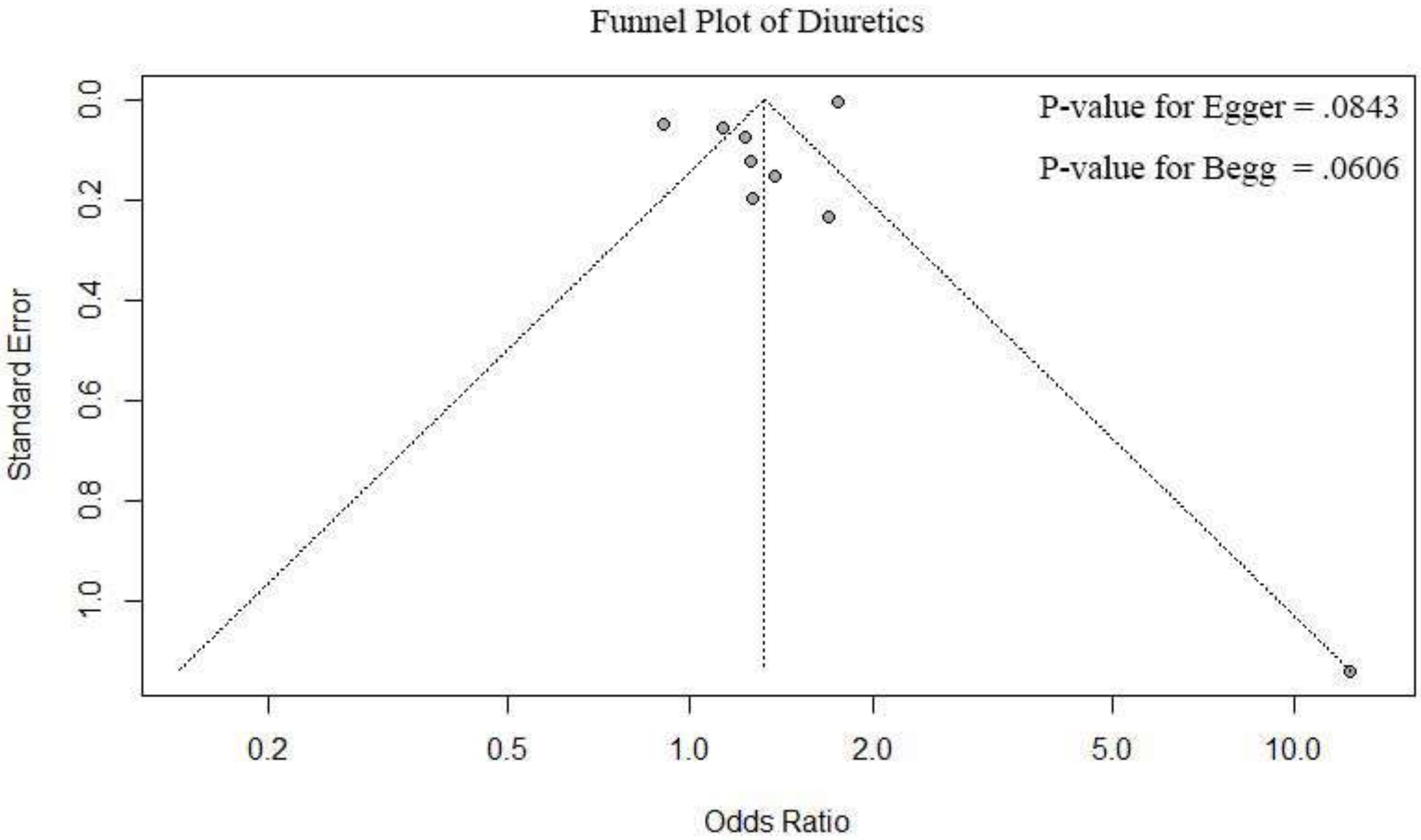

SUPPLEMENTAL DIGITAL CONTENT 39

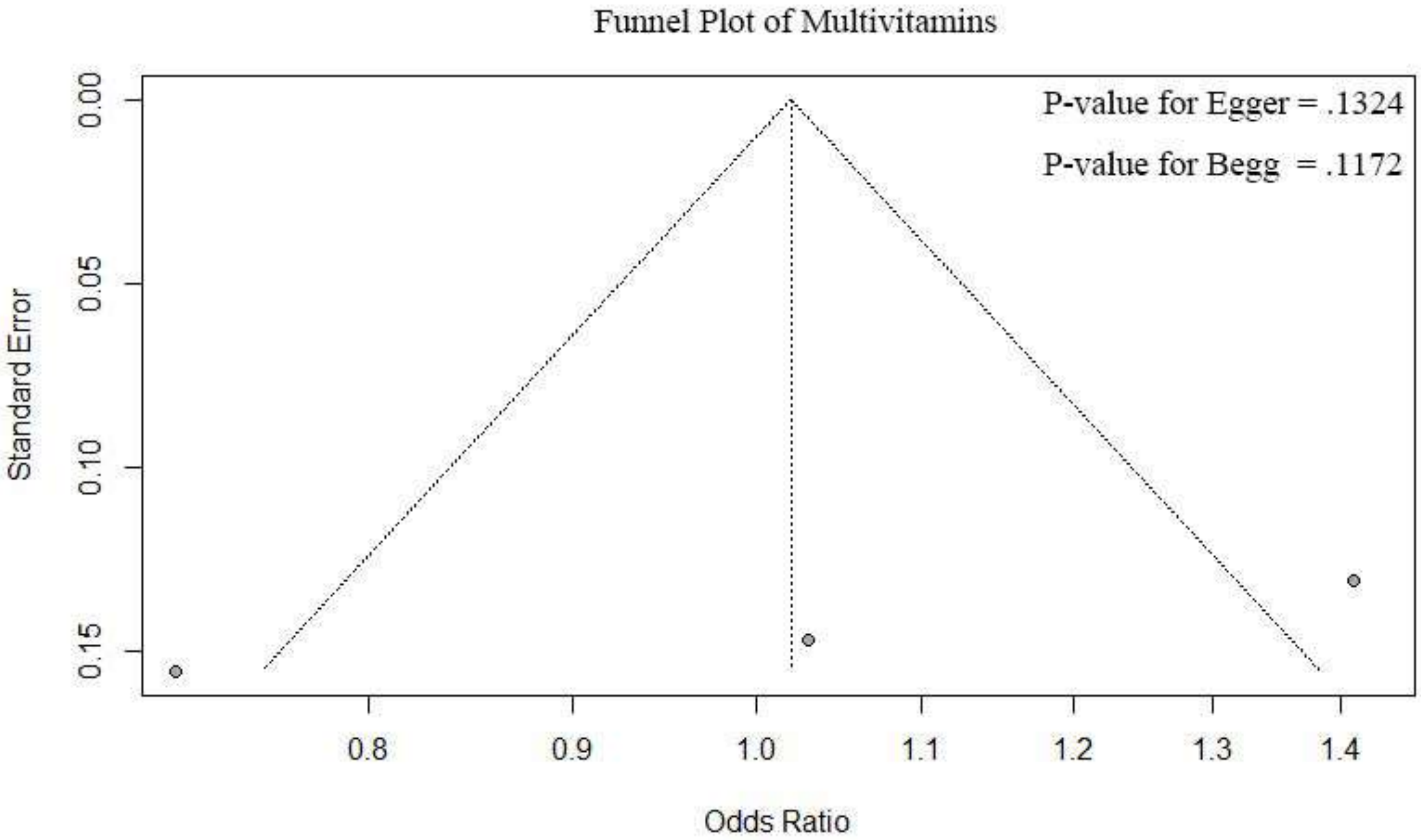

SUPPLEMENTAL DIGITAL CONTENT 40

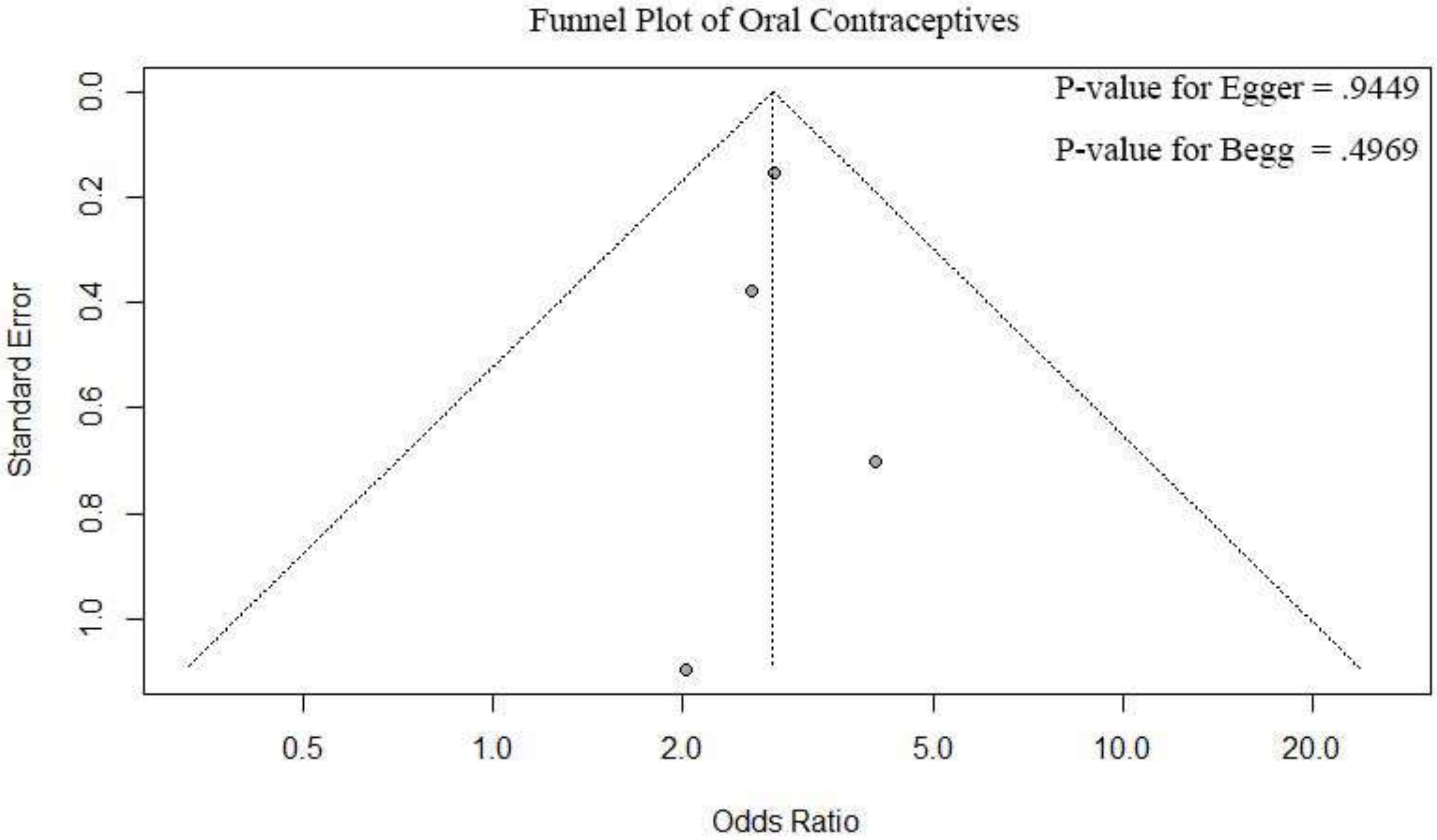

SUPPLEMENTAL DIGITAL CONTENT 41

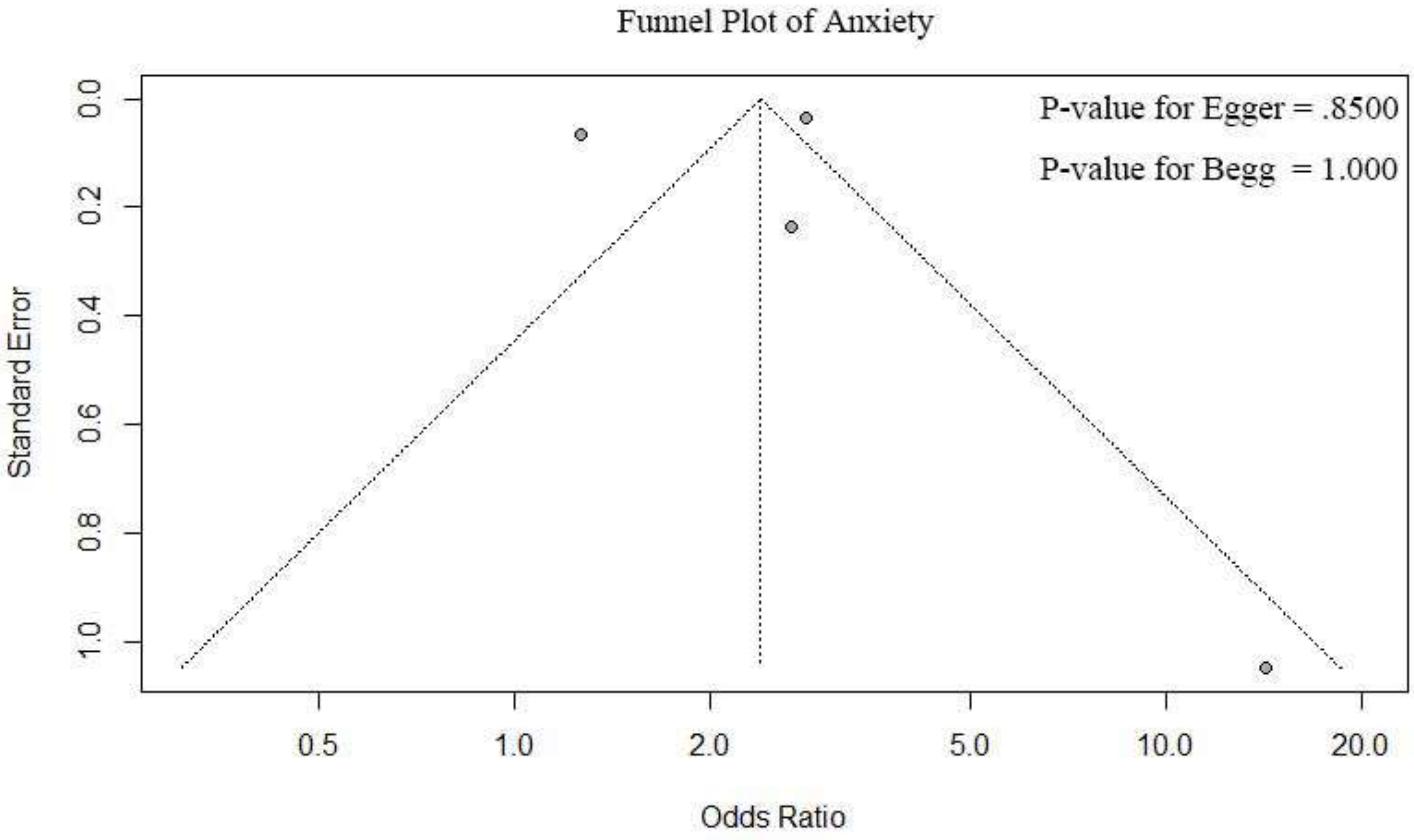

SUPPLEMENTAL DIGITAL CONTENT 42

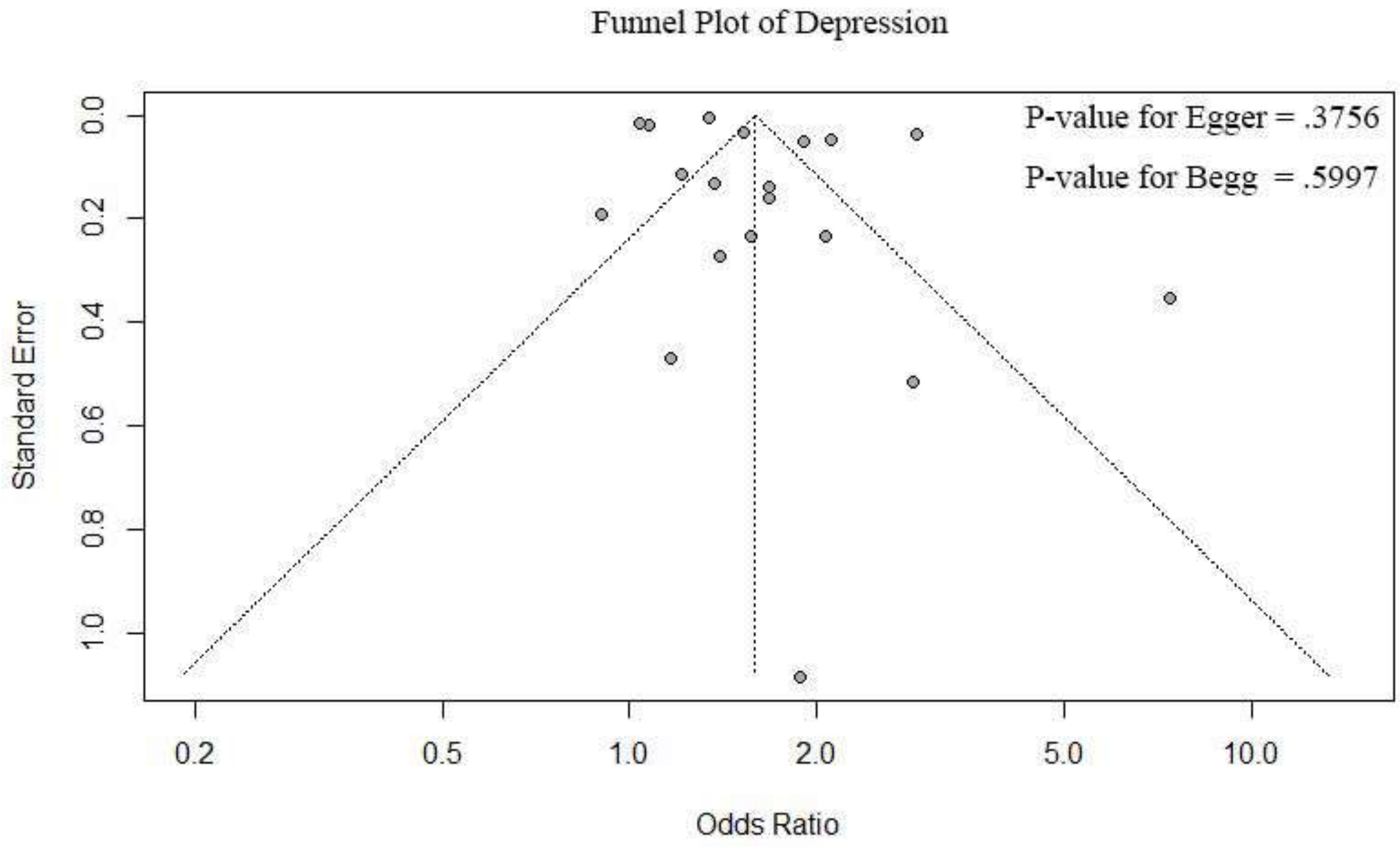

SUPPLEMENTAL DIGITAL CONTENT 43

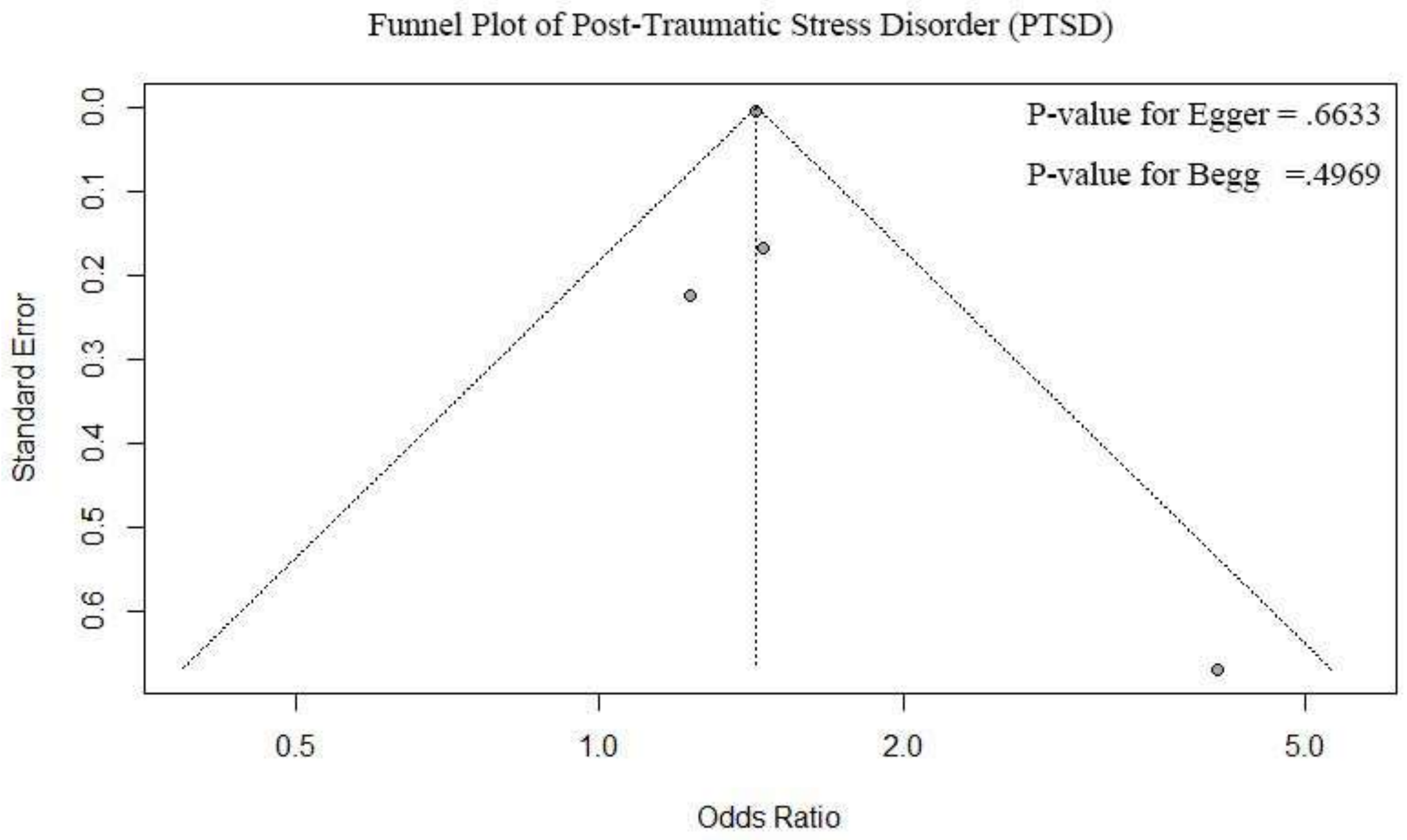

SUPPLEMENTAL DIGITAL CONTENT 44

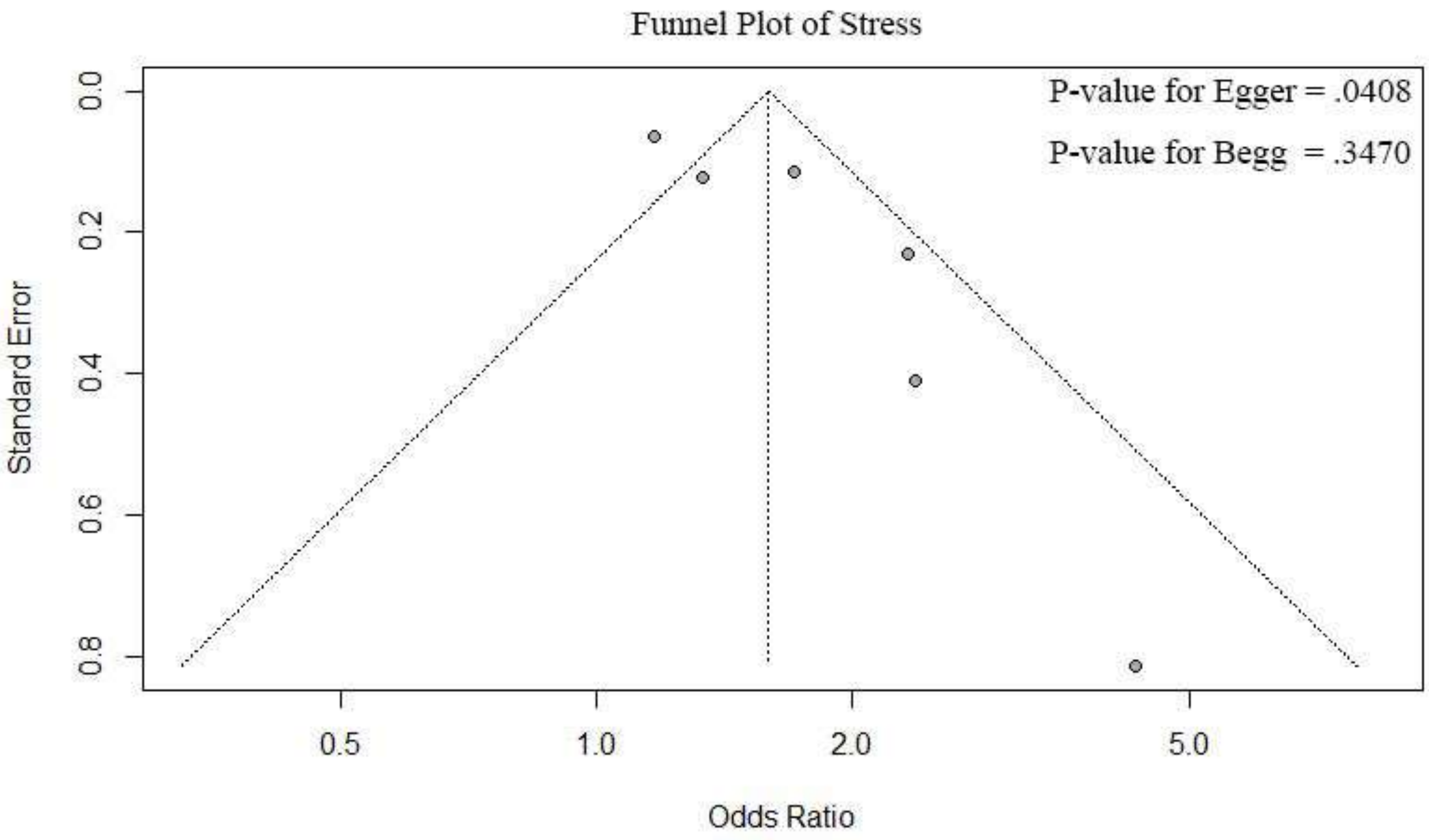

SUPPLEMENTAL DIGITAL CONTENT 45

Sensitivity Analysis of Hispanic Ethnicity

Study	Odds Ratio IV, Random, 95% CI
Omitting Galor et al (2012)	1.24 [0.93, 1.65]
Omitting Schaumberg et al (2003)	1.26 [0.85, 1.85]
Omitting Schaumberg et al (2009)	1.34 [0.90, 2.00]
Omitting Fernandez et al(2013)	1.34 [0.91, 1.96]
Omitting Farrand et al (2017)	1.33 [0.88, 1.99]
Omitting Lee et al(2017)	1.47 [1.25, 1.73]
**Total (95% CI)**	**1.33 [0.93, 1.90]**

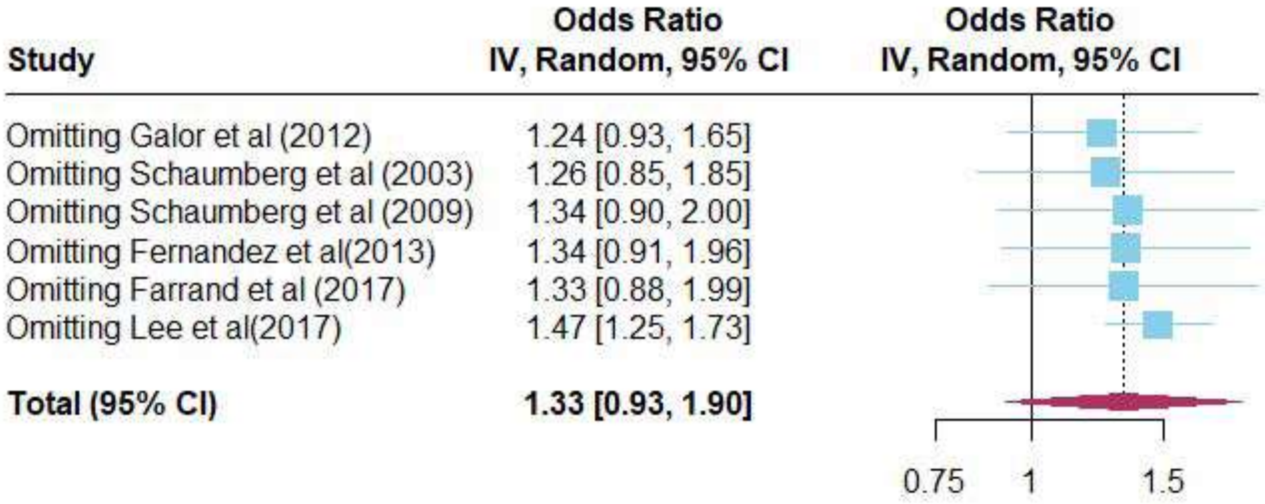

SUPPLEMENTAL DIGITAL CONTENT 46

Sensitivity Analysis of Menopause

Study	Odds Ratio IV, Random, 95% CI
Omitting Olaniyan et al (2016)	1.21 [0.84, 1.75]
Omitting Choi et al (2020)	2.30 [0.90, 5.84]
Omitting Wang et al (2021)	2.19 [0.69, 6.90]
Omitting García-Marqués et al (2022)	1.31 [0.75, 2.27]
**Total (95% CI)**	**1.52 [0.84, 2.74]**

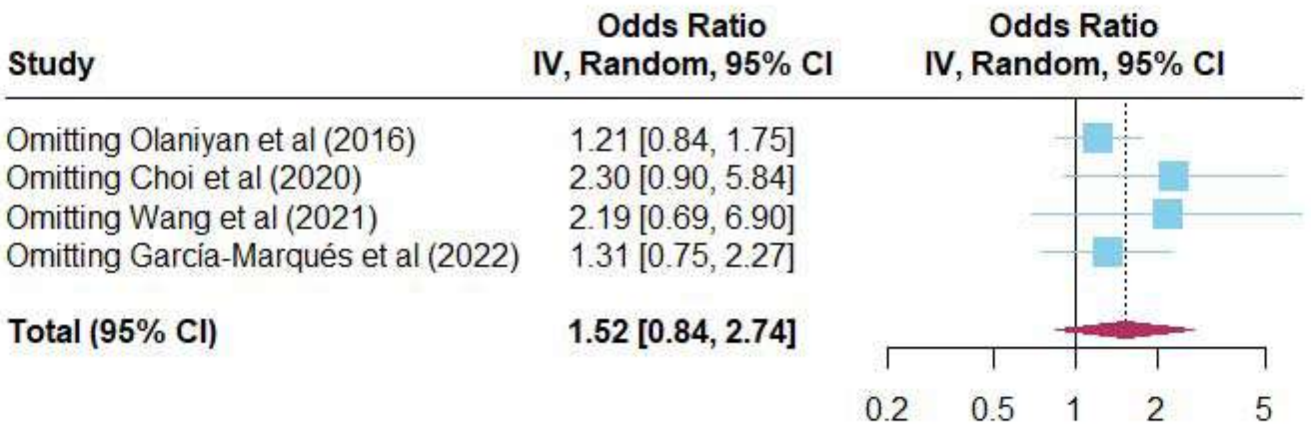

SUPPLEMENTAL DIGITAL CONTENT 47

Sensitivity Analysis of Alcohol

Study	Odds Ratio IV, Random, 95% CI
Omitting Galor et al(2011)	1.15 [1.00, 1.33]
Omitting Galor et al (2012)	1.19 [0.97, 1.46]
Omitting Yilmaz et al(2015)	1.18 [1.03, 1.35]
Omitting Ben-Eli et al (2019)	1.23 [1.07, 1.40]
Omitting Chia et al(2003)	1.23 [1.07, 1.41]
Omitting Guo et al (2010)	1.20 [1.04, 1.38]
Omitting Ahn et al (2014)	1.19 [1.03, 1.37]
Omitting Tan et al (2015)	1.18 [1.03, 1.35]
Omitting Chung et al (2016)	1.20 [1.05, 1.38]
Omitting Gong et al (2017)	1.19 [1.03, 1.36]
Omitting Asiedu et al (2017)	1.18 [1.03, 1.35]
Omitting Ferrero et al (2018)	1.19 [1.04, 1.36]
Omitting Kim et al (2019)	1.21 [1.05, 1.39]
Omitting Wu et al (2021)	1.19 [1.04, 1.36]
Omitting García-Marqués et al (2022)	1.18 [1.03, 1.35]
Omitting Garg et al(2022)	1.08 [0.96, 1.22]
Omitting Magno et al(2021)	1.22 [1.00, 1.48]
**Total (95% CI)**	**1.18 [1.03, 1.35]**

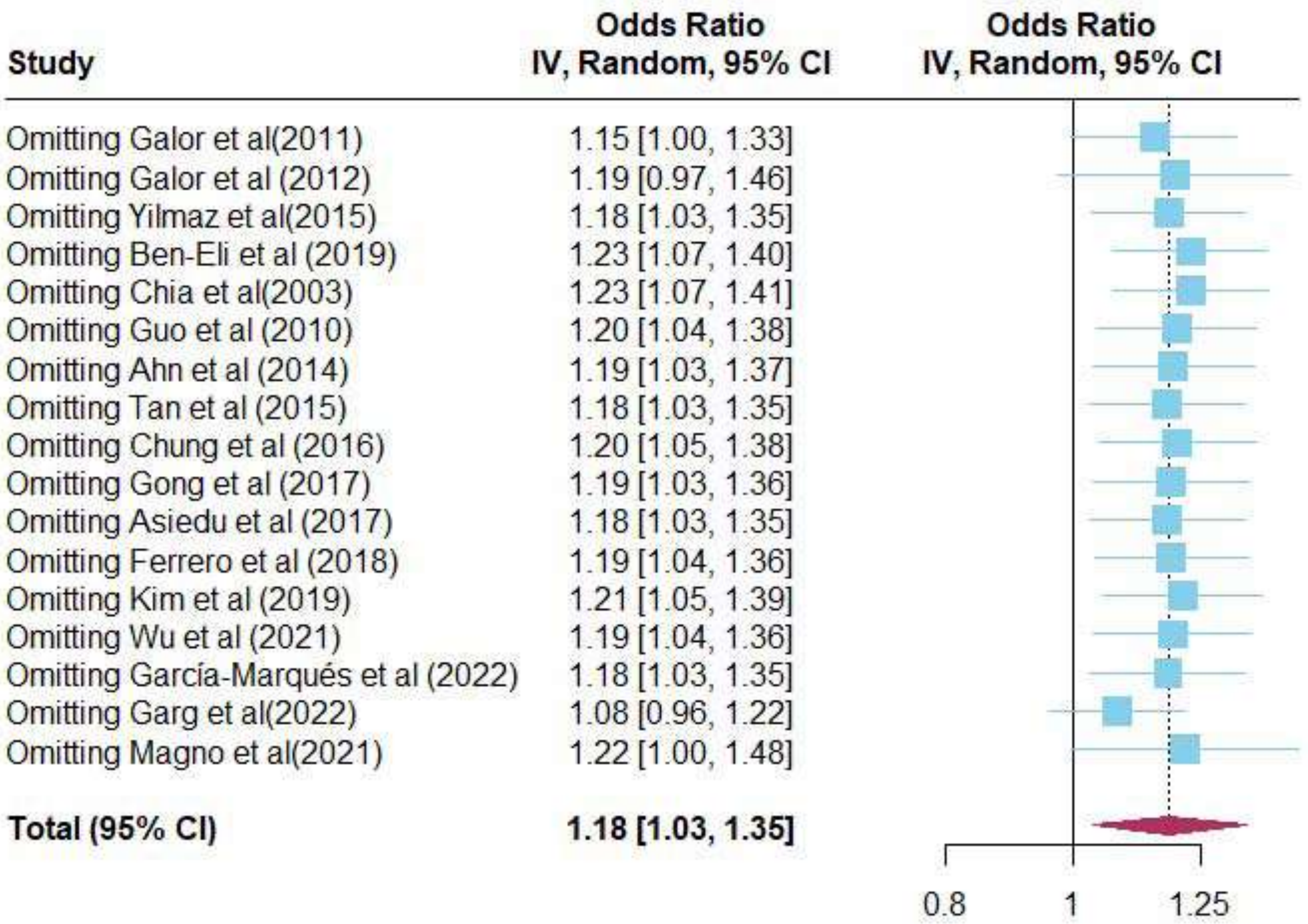

SUPPLEMENTAL DIGITAL CONTENT 48

Sensitivity Analysis of Smoking

Study	Odds Ratio IV, Random, 95% CI
Omitting Ben-Eli et al (2019)	1.18 [1.08, 1.30]
Omitting Uchino et al (2008)	1.20 [1.09, 1.31]
Omitting Guo et al (2010)	1.18 [1.08, 1.30]
Omitting Ahn et al (2014)	1.20 [1.09, 1.32]
Omitting Bakkar et al (2016)	1.18 [1.07, 1.29]
Omitting Gong et al (2017)	1.18 [1.08, 1.30]
Omitting Lee et al(2017)	1.20 [1.05, 1.37]
Omitting Titiyal et al (2018)	1.13 [1.04, 1.23]
Omitting Ferrero et al (2018)	1.19 [1.09, 1.31]
Omitting Inomata et al (2019)	1.15 [1.05, 1.26]
Omitting Inomata et al (2020)	1.14 [1.05, 1.25]
Omitting Shanti et al (2020)	1.19 [1.08, 1.30]
Omitting Tandon et al (2020)	1.18 [1.07, 1.30]
Omitting Vehof et al (2021)	1.21 [1.10, 1.33]
Omitting Chatterjee et al (2021)	1.19 [1.07, 1.33]
Omitting Yang et al (2021)	1.17 [1.07, 1.29]
Omitting Khorshed et al (2022)	1.16 [1.06, 1.26]
Omitting García-Marqués et al (2022)	1.17 [1.07, 1.28]
Omitting Dossari et al(2022)	1.17 [1.07, 1.28]
Omitting Cartes et al(2022)	1.17 [1.07, 1.28]
**Total (95% CI)**	**1.18 [1.07, 1.29]**

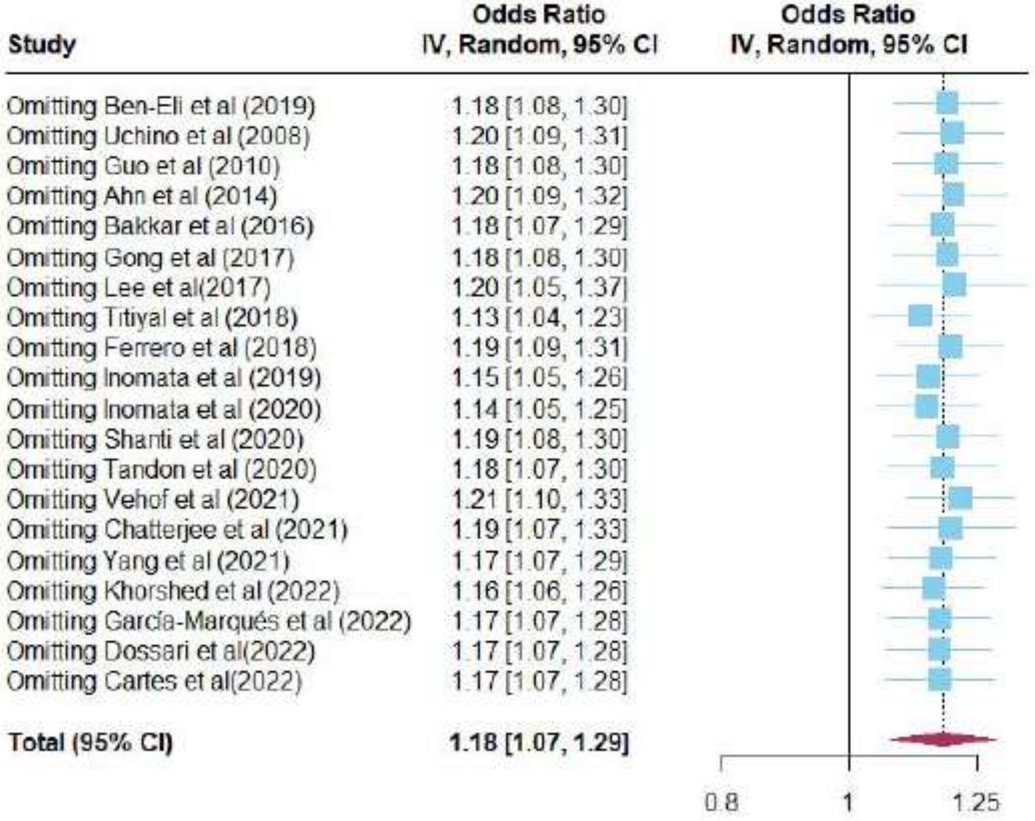

SUPPLEMENTAL DIGITAL CONTENT 49

Sensitivity Analysis of Past Smoking

Study	Odds Ratio IV, Random, 95% CI
Omitting Lee et al (2002)	1.06 [0.91, 1.22]
Omitting Malet et al (2013)	1.09 [0.98, 1.22]
Omitting Fernandez et al(2013)	1.06 [0.93, 1.20]
Omitting Kim et al (2019)	1.09 [1.04, 1.15]
Omitting Vehof et al (2021)	1.03 [0.81, 1.31]
Omitting Moss et al (2000)	1.01 [0.85, 1.21]
**Total (95% CI)**	**1.07 [0.94, 1.21]**

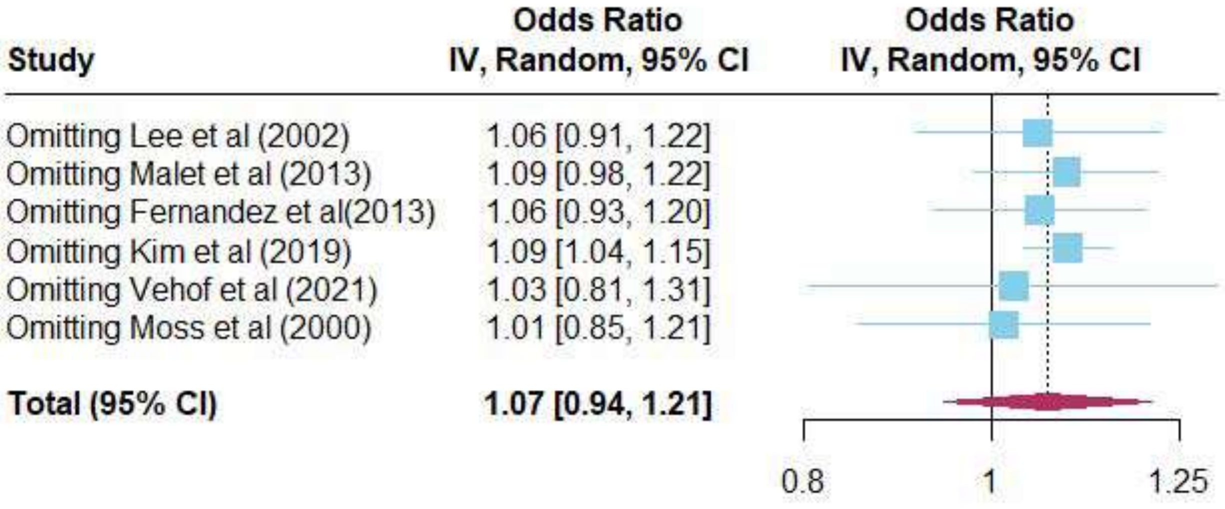

SUPPLEMENTAL DIGITAL CONTENT 50

Sensitivity Analysis of Current Smoking

Study	Odds Ratio IV, Random, 95% CI
Omitting Yilmaz et al(2015)	1.02 [0.81, 1.28]
Omitting Lee et al (2002)	1.01 [0.80, 1.27]
Omitting Chia et al(2003)	1.09 [0.88, 1.35]
Omitting Malet et al (2013)	1.06 [0.85, 1.32]
Omitting Uchino et al(2013)	1.07 [0.85, 1.34]
Omitting Fernandez et al(2013)	1.10 [0.90, 1.34]
Omitting Chung et al (2016)	1.06 [0.85, 1.34]
Omitting Alshamrani et al (2017)	1.01 [0.80, 1.28]
Omitting Kim et al (2019)	1.08 [0.86, 1.35]
Omitting Hu et al (2021)	1.08 [0.86, 1.34]
Omitting Choi et al (2021)	1.07 [0.86, 1.33]
Omitting Wu et al (2021)	1.01 [0.80, 1.28]
Omitting Moss et al (2000)	1.00 [0.82, 1.22]
**Total (95% CI)**	**1.05 [0.85, 1.30]**

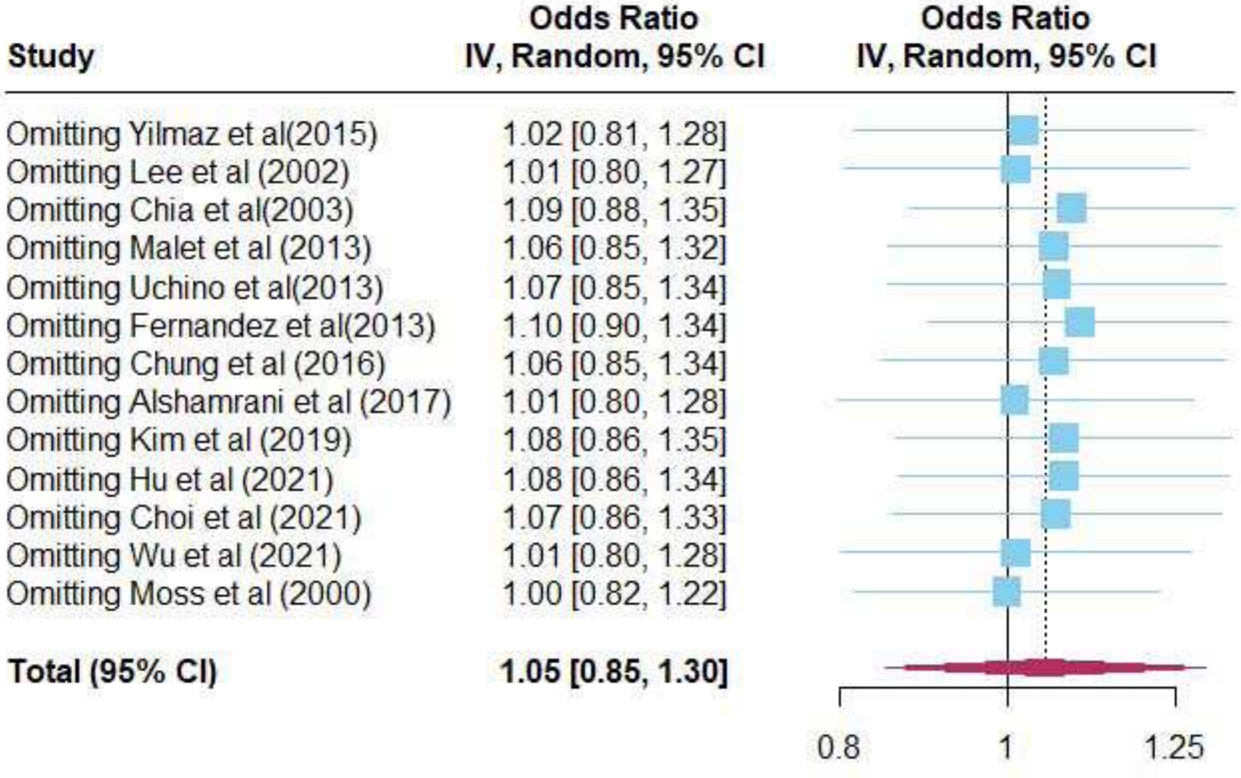

SUPPLEMENTAL DIGITAL CONTENT 51

Sensitivity Analysis of Rosacea or Acne

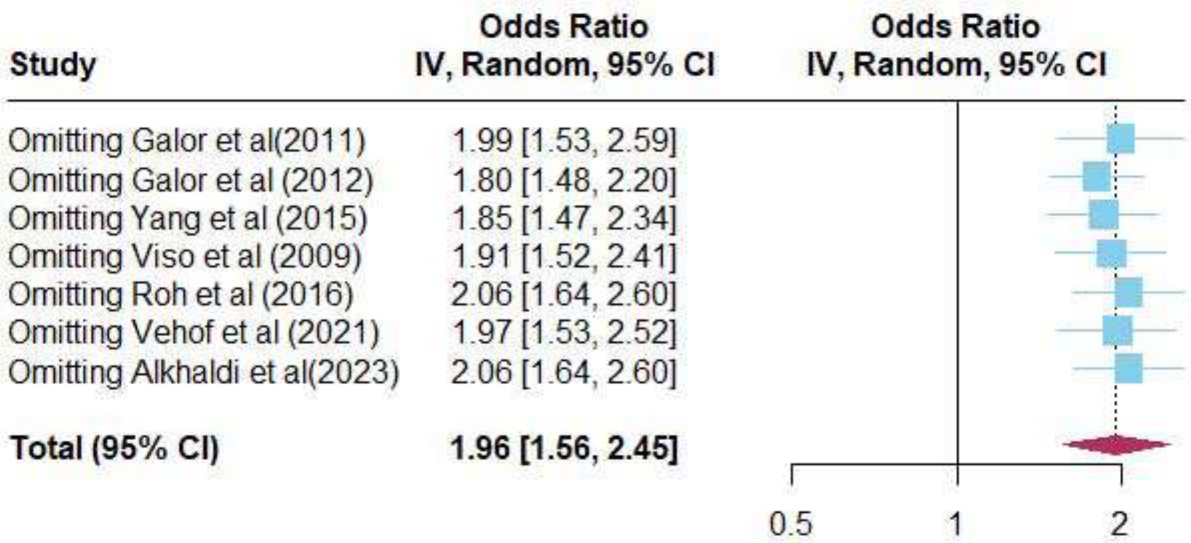

SUPPLEMENTAL DIGITAL CONTENT 52

Sensitivity Analysis of Allergic Conjunctivitis

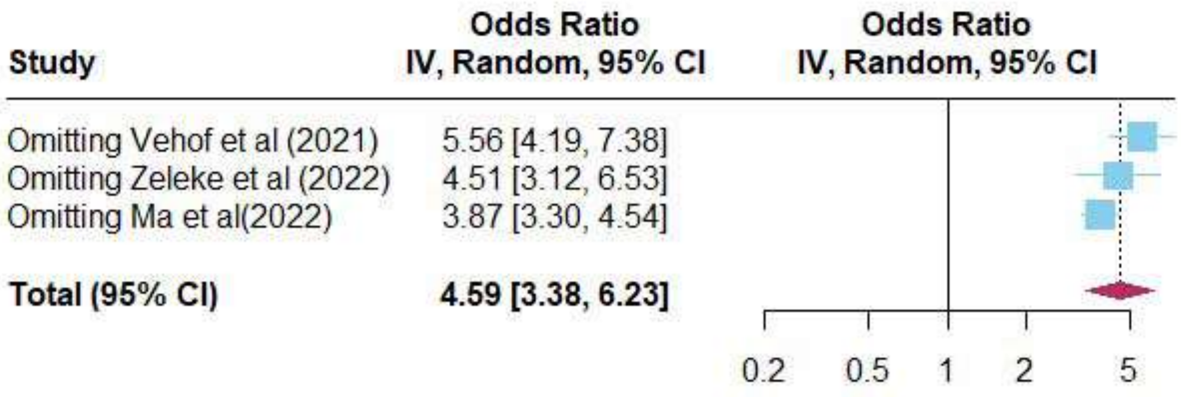

SUPPLEMENTAL DIGITAL CONTENT 53

Sensitivity Analysis of Pterygium

Study	Odds Ratio IV, Random, 95% CI
Omitting Lee et al (2002)	1.68 [0.72, 3.89]
Omitting Guo et al (2010)	1.44 [0.97, 2.13]
Omitting Viso et al(2011)	2.07 [1.20, 3.57]
Omitting Samuel et al(2012)	1.99 [1.12, 3.55]
**Total (95% CI)**	**1.78 [1.05, 3.00]**

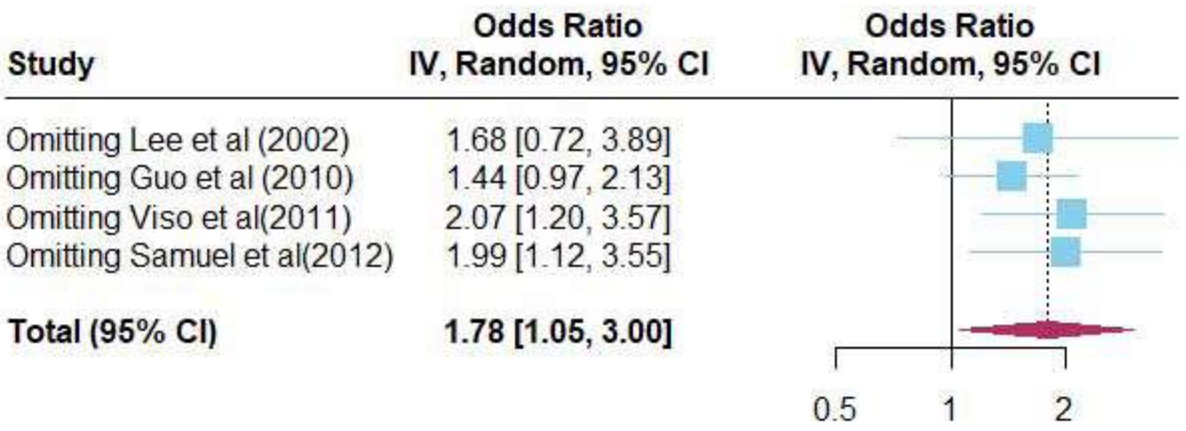

SUPPLEMENTAL DIGITAL CONTENT 54

Sensitivity Analysis of Refractive Surgery

Study	Odds Ratio IV, Random, 95% CI	Odds Ratio IV, Random, 95% CI
Omitting Zhang et al (2019)	2.22 [1.35, 3.65]	
Omitting Yu et al (2019)	1.97 [0.95, 4.12]	
Omitting Vehof et al (2021)	1.54 [1.17, 2.03]	
Omitting Alkhaldi et al(2023)	1.94 [1.21, 3.11]	
**Total (95% CI)**	**1.90 [1.28, 2.84]**	

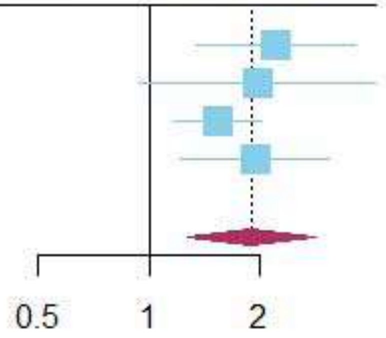

SUPPLEMENTAL DIGITAL CONTENT 55

Sensitivity Analysis of Diabetes

Study	Odds Ratio IV, Random, 95% CI
Omitting Galor et al (2012)	1.17 [1.07, 1.28]
Omitting Yang et al (2015)	1.13 [1.05, 1.21]
Omitting Chia et al(2003)	1.14 [1.06, 1.22]
Omitting Schaumberg et al (2009)	1.15 [1.07, 1.24]
Omitting Viso et al (2009)	1.14 [1.06, 1.22]
Omitting Vehof et al(2014)	1.13 [1.05, 1.21]
Omitting Yoon et al(2016)	1.16 [1.07, 1.25]
Omitting Alshamrani et al (2017)	1.12 [1.05, 1.19]
Omitting Gong et al (2017)	1.14 [1.06, 1.22]
Omitting Ferrero et al (2018)	1.14 [1.06, 1.22]
Omitting Zhang et al (2019)	1.17 [1.08, 1.27]
Omitting Inomata et al (2019)	1.14 [1.06, 1.22]
Omitting Kim et al (2019)	1.14 [1.06, 1.23]
Omitting Shanti et al (2020)	1.14 [1.06, 1.23]
Omitting Tandon et al (2020)	1.14 [1.06, 1.22]
Omitting Inomata et al(2020)	1.14 [1.07, 1.23]
Omitting Vehof et al (2021)	1.11 [1.04, 1.19]
Omitting Choi et al (2021)	1.14 [1.06, 1.22]
Omitting Yang et al (2021)	1.14 [1.06, 1.22]
Omitting Wu et al (2021)	1.15 [1.07, 1.23]
Omitting Khorshed et al (2022)	1.13 [1.06, 1.20]
Omitting Dossari et al(2022)	1.14 [1.06, 1.22]
Omitting Garg et al(2022)	1.13 [1.06, 1.21]
Omitting Moss et al (2000)	1.13 [1.05, 1.21]
**Total (95% CI)**	**1.14 [1.06, 1.22]**

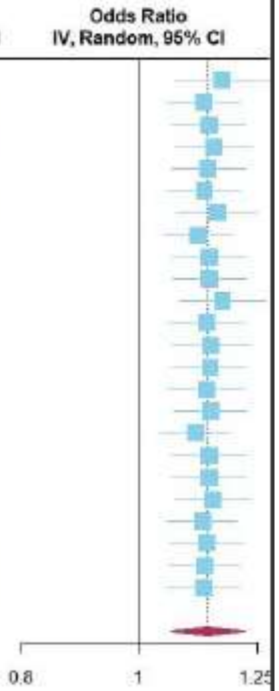

SUPPLEMENTAL DIGITAL CONTENT 56

Sensitivity Analysis of Thyroid Disease

Study	Odds Ratio IV, Random, 95% CI
Omitting Galor et al(2011)	1.58 [1.36, 1.83]
Omitting Galor et al (2012)	1.56 [1.34, 1.81]
Omitting Wang et al (2012)	1.55 [1.32, 1.82]
Omitting Malet et al (2013)	1.59 [1.37, 1.84]
Omitting Vehof et al(2014)	1.57 [1.35, 1.83]
Omitting Paulsen et al (2014)	1.57 [1.35, 1.83]
Omitting Ahn et al (2014)	1.56 [1.34, 1.82]
Omitting Yoon et al(2016)	1.63 [1.50, 1.77]
Omitting Roh et al (2016)	1.56 [1.34, 1.82]
Omitting Roh et al(2016)	1.56 [1.34, 1.82]
Omitting Alshamrani et al (2017)	1.58 [1.36, 1.84]
Omitting Zhang et al (2019)	1.59 [1.36, 1.85]
Omitting Yu et al (2019)	1.59 [1.37, 1.86]
Omitting Kim et al (2019)	1.58 [1.36, 1.84]
Omitting Wang et al (2021)	1.55 [1.33, 1.79]
Omitting Bikbov et al(2022)	1.57 [1.35, 1.83]
Omitting García-Marqués et al (2022)	1.59 [1.37, 1.84]
Omitting Dossari et al(2022)	1.56 [1.34, 1.81]
Omitting Garcia-Queiruga et al(2023)	1.58 [1.36, 1.83]
Omitting Alkhaldi et al(2023)	1.58 [1.36, 1.83]
Omitting Moss et al (2008)	1.56 [1.34, 1.82]
**Total (95% CI)**	**1.57 [1.36, 1.82]**

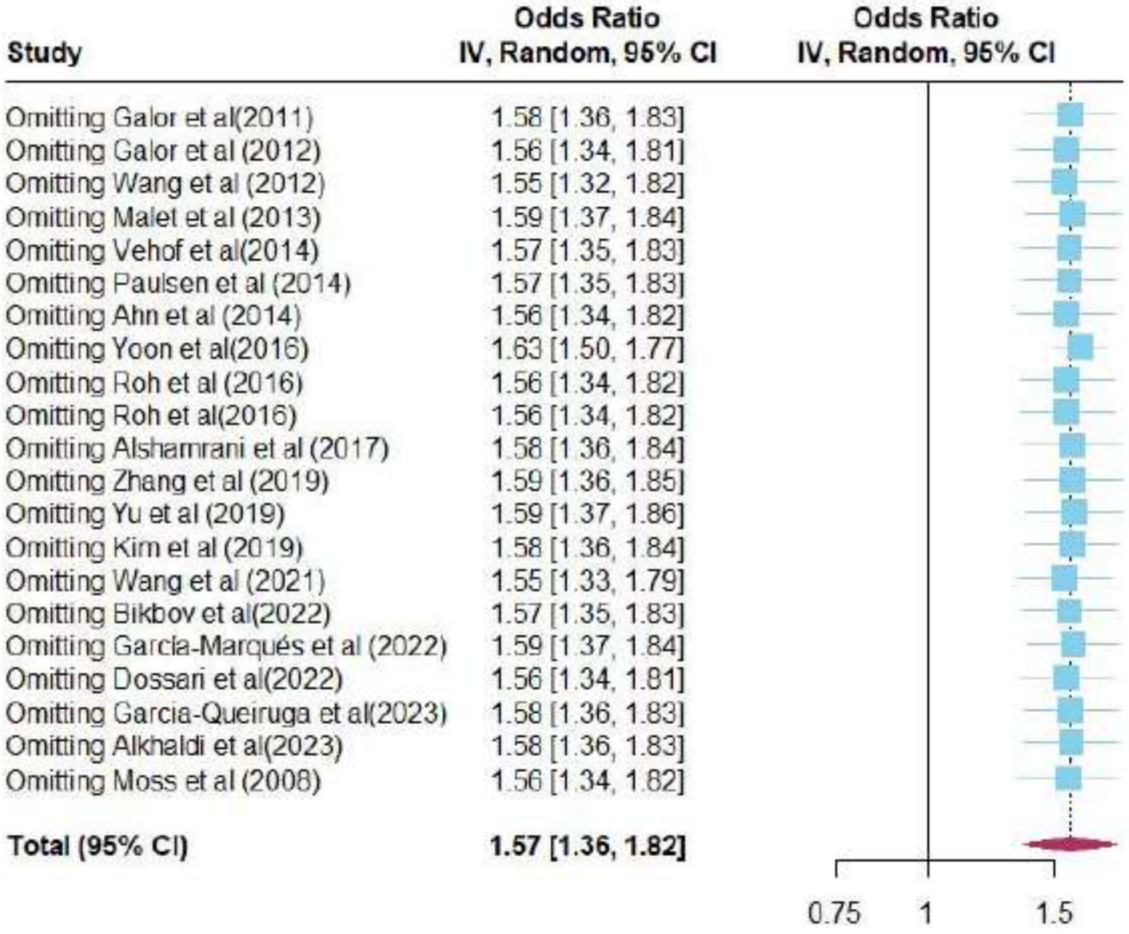

SUPPLEMENTAL DIGITAL CONTENT 57

Sensitivity Analysis of Thyroid Disease

Study	Odds Ratio IV, Random, 95% CI
Omitting Galor et al(2011)	1.58 [1.36, 1.83]
Omitting Galor et al (2012)	1.56 [1.34, 1.81]
Omitting Wang et al (2012)	1.55 [1.32, 1.82]
Omitting Malet et al (2013)	1.59 [1.37, 1.84]
Omitting Vehof et al(2014)	1.57 [1.35, 1.83]
Omitting Paulsen et al (2014)	1.57 [1.35, 1.83]
Omitting Ahn et al (2014)	1.56 [1.34, 1.82]
Omitting Yoon et al(2016)	1.63 [1.50, 1.77]
Omitting Roh et al (2016)	1.56 [1.34, 1.82]
Omitting Roh et al(2016)	1.56 [1.34, 1.82]
Omitting Alshamrani et al (2017)	1.58 [1.36, 1.84]
Omitting Zhang et al (2019)	1.59 [1.36, 1.85]
Omitting Yu et al (2019)	1.59 [1.37, 1.86]
Omitting Kim et al (2019)	1.58 [1.36, 1.84]
Omitting Wang et al (2021)	1.55 [1.33, 1.79]
Omitting Bikbov et al(2022)	1.57 [1.35, 1.83]
Omitting García-Marqués et al (2022)	1.59 [1.37, 1.84]
Omitting Dossari et al(2022)	1.56 [1.34, 1.81]
Omitting Garcia-Queiruga et al(2023)	1.58 [1.36, 1.83]
Omitting Alkhaldi et al(2023)	1.58 [1.36, 1.83]
Omitting Moss et al (2008)	1.56 [1.34, 1.82]
**Total (95% CI)**	**1.57 [1.36, 1.82]**

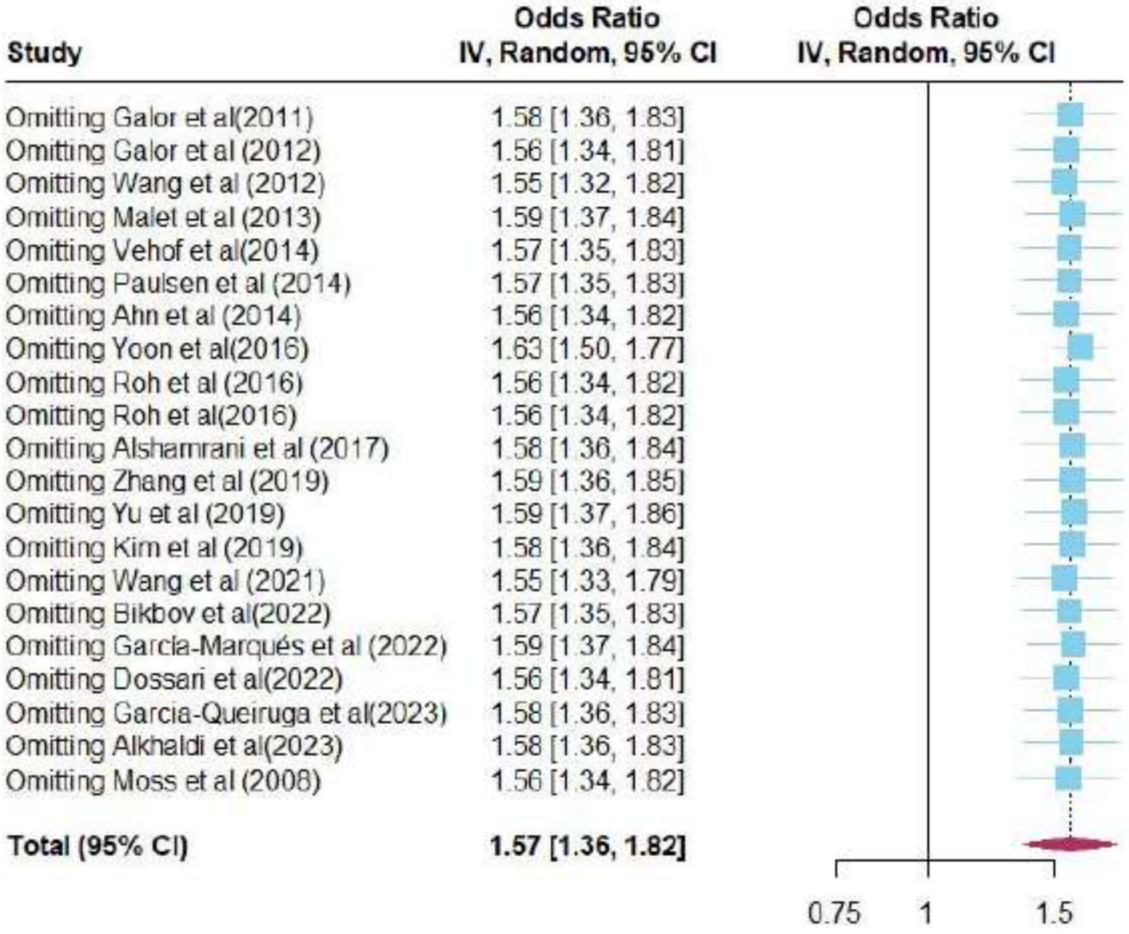

SUPPLEMENTAL DIGITAL CONTENT 58

Sensitivity Analysis of Betablockers

Study	Odds Ratio IV, Random, 95% CI
Omitting Galor et al (2012)	1.25 [0.93, 1.68]
Omitting Chen et al(2015)	1.27 [0.92, 1.77]
Omitting Malet et al (2013)	1.53 [1.33, 1.76]
Omitting Ferrero et al (2018)	1.34 [1.08, 1.66]
Omitting Wolpert et al (2021)	1.48 [1.25, 1.76]
**Total (95% CI)**	**1.38 [1.15, 1.67]**

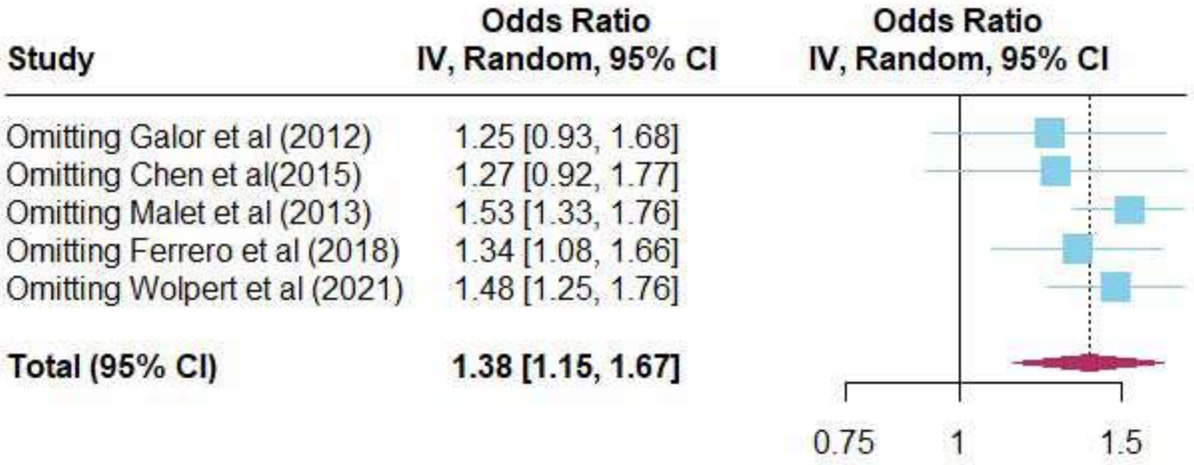

SUPPLEMENTAL DIGITAL CONTENT 59

Sensitivity Analysis of Diuretics

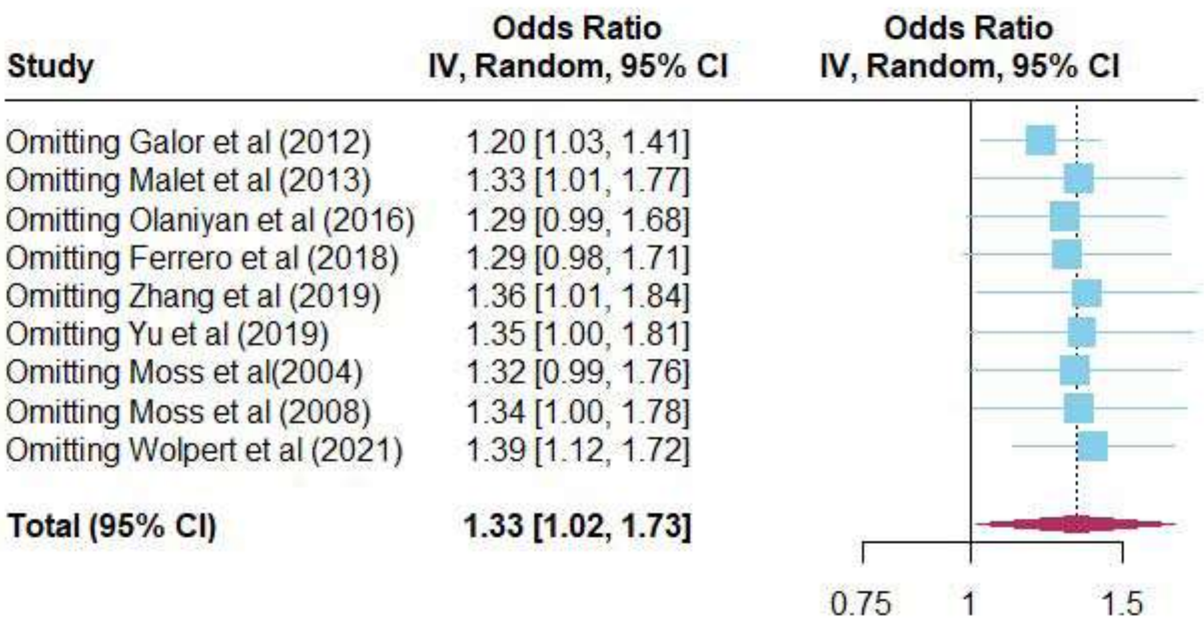

SUPPLEMENTAL DIGITAL CONTENT 60

Sensitivity Analysis of Multivitamins

Study	Odds Ratio IV, Random, 95% CI	Odds Ratio IV, Random, 95% CI
Omitting Yang et al (2015)	1.21 [0.89, 1.65]	
Omitting Paulsen et al (2014)	1.01 [0.52, 1.96]	
Omitting Moss et al (2000)	0.86 [0.60, 1.23]	
**Total (95% CI)**	**1.02 [0.69, 1.50]**	

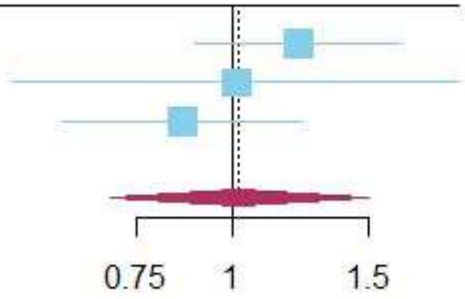

SUPPLEMENTAL DIGITAL CONTENT 61

Sensitivity Analysis of Oral Contraceptives

Study	Odds Ratio IV, Random, 95% CI
Omitting Asiedu et al (2017)	2.75 [2.08, 3.62]
Omitting Wang et al (2021)	2.82 [2.11, 3.77]
Omitting García-Marqués et al (2022)	2.80 [2.13, 3.68]
Omitting He et al (2022)	2.77 [1.48, 5.18]
**Total (95% CI)**	**2.79 [2.13, 3.65]**

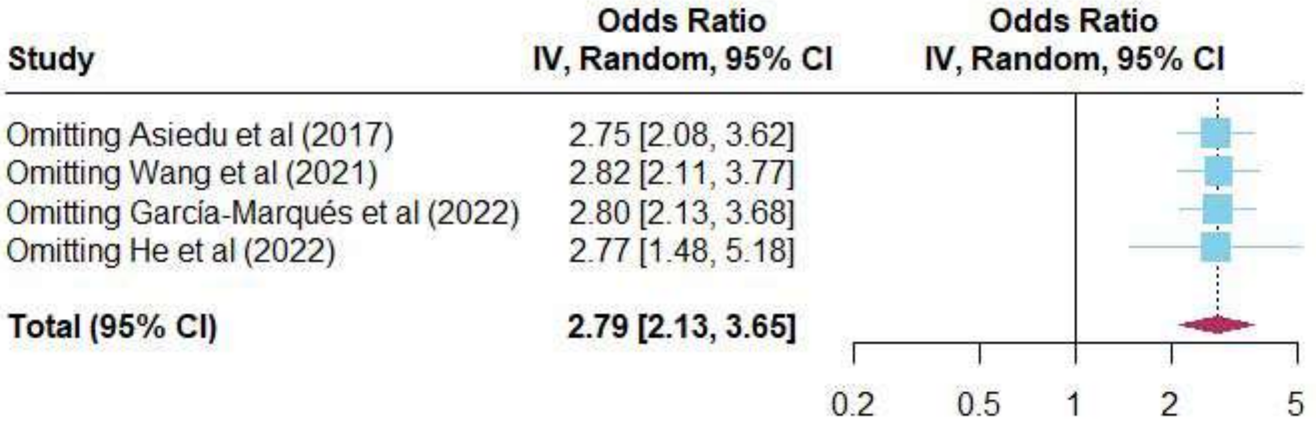

SUPPLEMENTAL DIGITAL CONTENT 62

Sensitivity Analysis of Anxiety

Study	Odds Ratio IV, Random, 95% CI
Omitting van der Vaart et al(2015)	2.28 [1.02, 5.10]
Omitting Yilmaz et al(2015)	2.32 [1.10, 4.89]
Omitting Vehof et al (2021)	2.82 [2.29, 3.49]
Omitting García-Marqués et al (2022)	2.09 [1.11, 3.91]
**Total (95% CI)**	**2.39 [1.30, 4.39]**

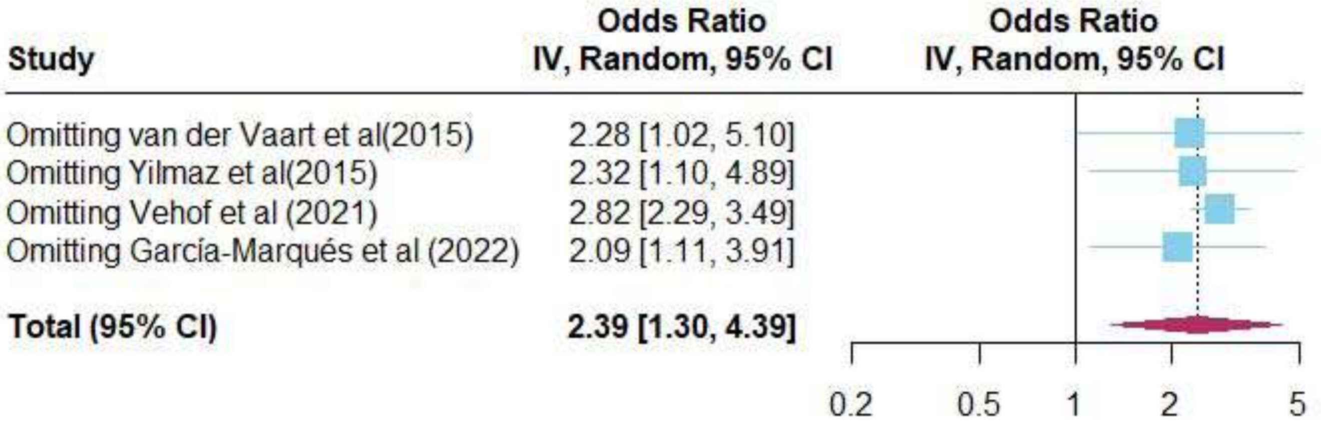

SUPPLEMENTAL DIGITAL CONTENT 63

Sensitivity Analysis of Depression

Study	Odds Ratio IV, Random, 95% CI
Omitting Galor et al(2011)	1.60 [1.31, 1.96]
Omitting Galor et al (2012)	1.64 [1.35, 2.01]
Omitting Hallak et al(2015)	1.60 [1.32, 1.93]
Omitting van der Vaart et al(2015)	1.54 [1.30, 1.83]
Omitting Yilmaz et al(2015)	1.60 [1.32, 1.95]
Omitting Wang et al (2012)	1.59 [1.31, 1.94]
Omitting Fernandez et al(2013)	1.64 [1.35, 1.98]
Omitting Vehof et al(2014)	1.62 [1.32, 1.98]
Omitting Yoon et al(2016)	1.67 [1.38, 2.02]
Omitting Ferrero et al (2018)	1.63 [1.34, 1.99]
Omitting Inomata et al (2019)	1.62 [1.32, 1.98]
Omitting Kim et al (2019)	1.65 [1.36, 2.01]
Omitting Inomata et al (2020)	1.53 [1.30, 1.79]
Omitting Inomata et al(2020)	1.67 [1.39, 2.01]
Omitting Vehof et al (2021)	1.63 [1.33, 2.00]
Omitting Garcia-Marqués et al (2022)	1.62 [1.34, 1.95]
Omitting An et al (2022)	1.64 [1.34, 2.00]
Omitting Bikbov et al(2022)	1.67 [1.38, 2.02]
Omitting Cartes et al(2022)	1.62 [1.33, 1.98]
**Total (95% CI)**	**1.62 [1.34, 1.95]**

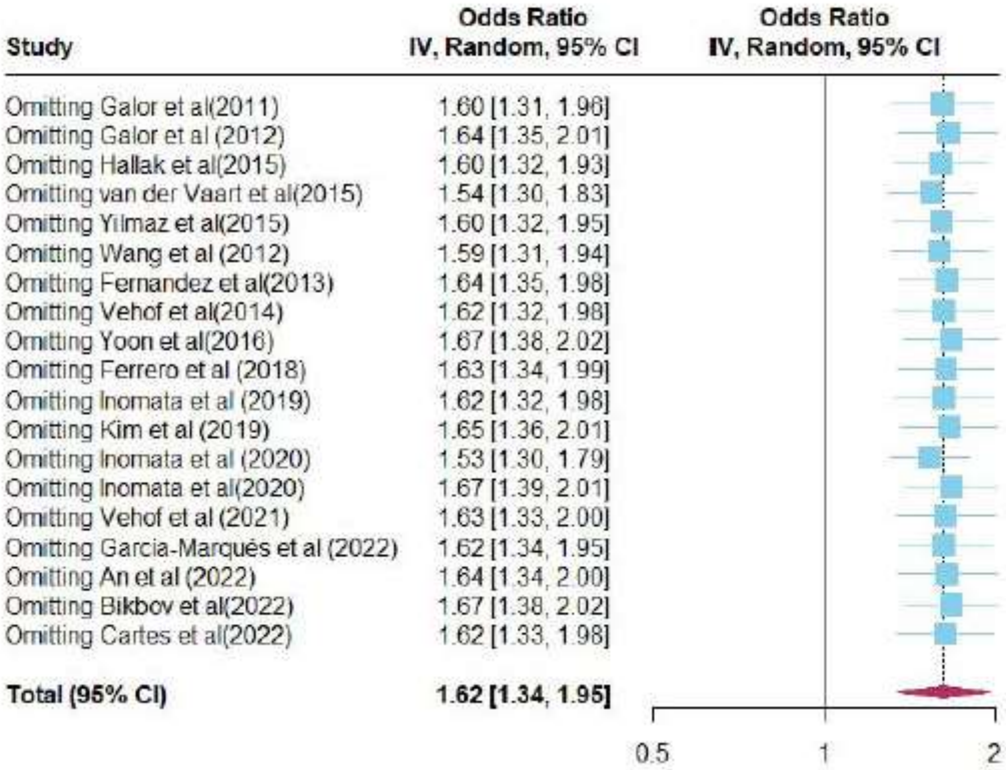

SUPPLEMENTAL DIGITAL CONTENT 64

Sensitivity Analysis of Post-Traumatic Stress Disorder (PTSD)

Study	Odds Ratio IV, Random, 95% CI
Omitting Galor et al (2012)	1.46 [1.02, 2.07]
Omitting Yang et al (2015)	1.43 [1.12, 1.83]
Omitting Fernandez et al(2013)	1.43 [1.42, 1.44]
Omitting Vehof et al (2021)	1.45 [1.26, 1.67]
**Total (95% CI)**	**1.43 [1.42, 1.45]**

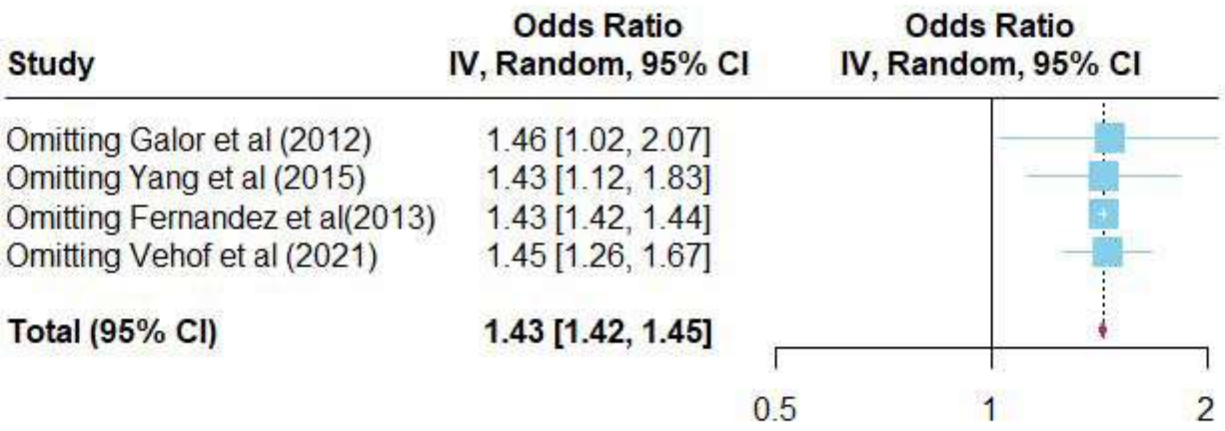

SUPPLEMENTAL DIGTIAL CONTENT 65

Sensitivity Analysis of Stress

Study	Odds Ratio IV, Random, 95% CI
Omitting Yilmaz et al(2015)	1.46 [1.15, 1.87]
Omitting Talens-Estarelles et al (2022)	1.54 [1.19, 2.00]
Omitting Chung et al (2016)	1.75 [1.23, 2.48]
Omitting Hyon et al (2019)	1.75 [1.36, 2.25]
Omitting García-Marqués et al (2022)	1.55 [1.21, 1.99]
Omitting An et al (2022)	1.57 [1.16, 2.13]
**Total (95% CI)**	**1.59 [1.24, 2.05]**

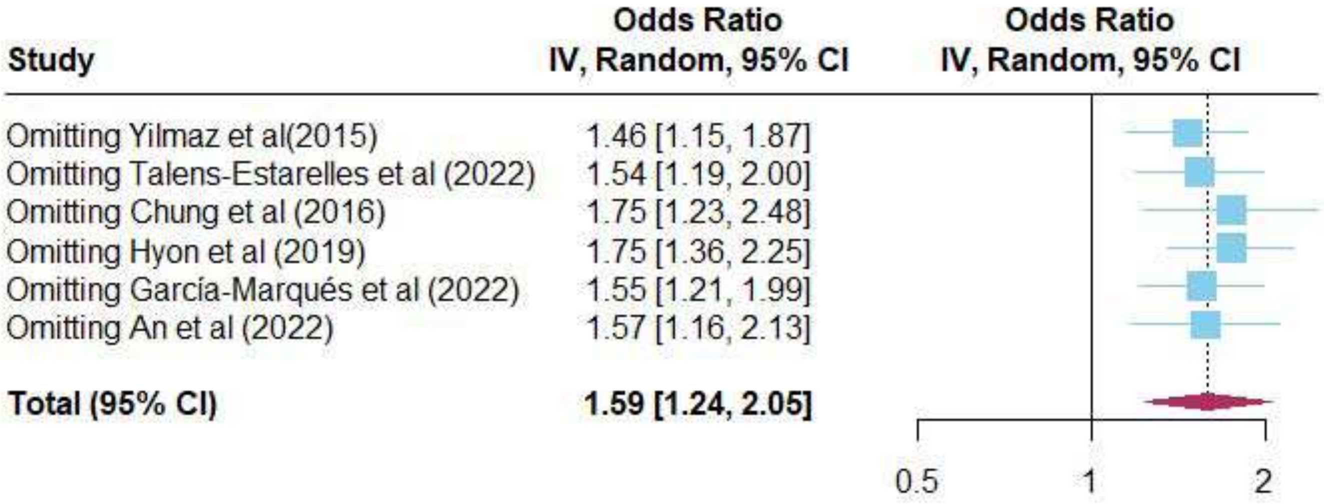

Supplement: Supplementary file 1 [file medi-103-e41019-s001.pdf]
